# Deciphering the benefits and intensity levels of primary metabolites from *Allium macrostemon* Bunge and *Allium chinense* G. Don

**DOI:** 10.1186/s13020-024-00957-3

**Published:** 2024-07-15

**Authors:** Zifei Qin, Yuan Li, Dongmei Liu, Yuzhuo Hua, Yuandong Lv, Xiaojian Zhang, Cailian Fan, Jing Yang

**Affiliations:** 1 Department of Pharmacy, The First Affiliated Hospital of Zhengzhou University, Zhengzhou, 450052 China; 2 College of Medicine, Henan Engineering Research Center of Funiu Mountain’s Medicinal Resources Utilization and Molecular Medicine, Pingdingshan University, Pingdingshan, 467000 China; 3 Hangzhou EXPECLIN Medical Technology Co., Ltd., Hangzhou, 311305 China; 4 Henan Engineering Research Center of Application & Translation of Precision Clinical Pharmacy, Zhengzhou, 450052 China

**Keywords:** *Allium* herbs, Apoptosis, H9c2 cell, Active components, Mass spectrometry imaging, Quantification

## Abstract

**Background:**

Allii Macrostemonis Bulbus is also named Xiebai in China. It is an edible vegetable, and also a famous herb for treating coronary heart disease. *Allium chinense* G. Don (ACGD) and *Allium macrostemon* Bunge (AMB) are it botanical sources. The aim of this study was to explore the cardioprotective effects, and decipher the visual spatial distribution and absolute content of primary metabolites derived from these two herbs.

**Methods:**

H9c2 cells were used to perform the hypoxia-reoxygenation (H/R)-induced myocardial injury model. Their protective effects were evaluated by apoptosis levels. Furthermore, matrix-assisted laser desorption/ionization time-of-flight tandem mass spectrometry imaging approach (MALDI-TOF MSI) was carried out to present the spatial location of primary metabolites including fatty acids, amino acids, carotenoids, and vitamins in these two *Allium* herbs. Multiple analytical methods were applied to perform quantitative analysis of these primary metabolites in AMB and ACGD bulbs by liquid chromatography tandem mass spectrometry (LC–MS).

**Results:**

First, AMB and ACGD extracts both could increase the cell viability in H9c2 cells, and attenuate H/R-induced injury. They markedly decreased apoptosis, accompanied by activating the BCL-2/BAX pathway. Further, MALDI-TOF MSI-based relative quantification results showed several amino acids, fatty acids, carotenoids, and vitamins were largely rich in the tunics and outside scales of fresh bulbs, while some primary metabolites were abundant in their developing flower buds. Absolute quantification results displayed total contents of amino acids in ACGD bulbs were higher than those in AMB, while total contents of fatty acids and vitamins provides opposite trends in these two *Allium* herbs. The total contents of carotenoids and trace elements showed no significant differences between AMB and ACGD samples.

**Conclusions:**

This study would be helpful to understand the myocardial injury protection effects of these two *Allium* herbs, and the spatial accumulation and quantitative content levels of their main nutrients.

**Graphical Abstract:**

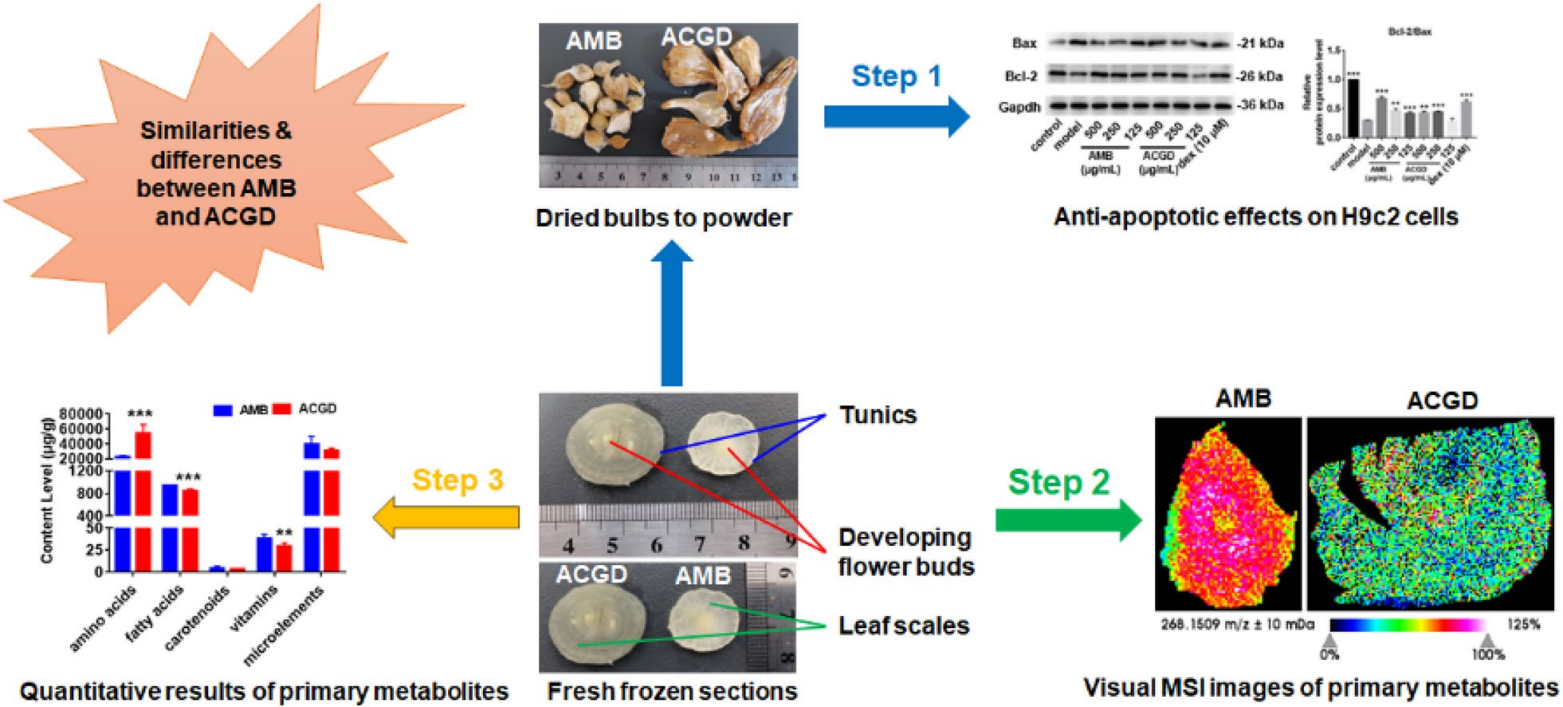

**Supplementary Information:**

The online version contains supplementary material available at 10.1186/s13020-024-00957-3.

## Highlights

1. Myocardial injury protection effects of two *Allium* extracts were characterized.
2. Visual spatial distribution of main primary metabolites was performed.
3. Absolute quantification was achieved by multiple conventional approaches.
4. Similarities and differences of these two *Allium* extracts were summarized.

**Supplementary Information:**

The online version contains supplementary material available at 10.1186/s13020-024-00957-3.

## Background

Allii Macrostemonis Bulbus (Xiebai in Chinese) was first recorded in Shennong Bencao Jing (Divine Farmer's Classic of Materia Medica) [1]. It has high nutritional and medical values, and is a homology of medicine and food crop [2]. As a vegetable, Xiebai is usually used as a popular flavoring due to high healthy values [3]. Further, as a famous herb, Xiebai is often used to improve and treat cardiovascular diseases. During the treatment, *Trichosanthes kirilowii* (Gualou in China) is the most common combination herb with Xiebai. Gualou-Xiebai Baijiu decoction, as a famous classical traditional Chinese medicine formula, is widely used as a basic recipe and modified according to symptoms in modern clinical practice in East Asia [1]. Sulfur-containing volatile organic compounds, nitrogenous compounds, and steroidal saponins are considered as its active components for anti-atherosclerosis effect, platelet aggregation inhibitory activity, and lipid-lowering activity [4–6].

**Fig. 1:**
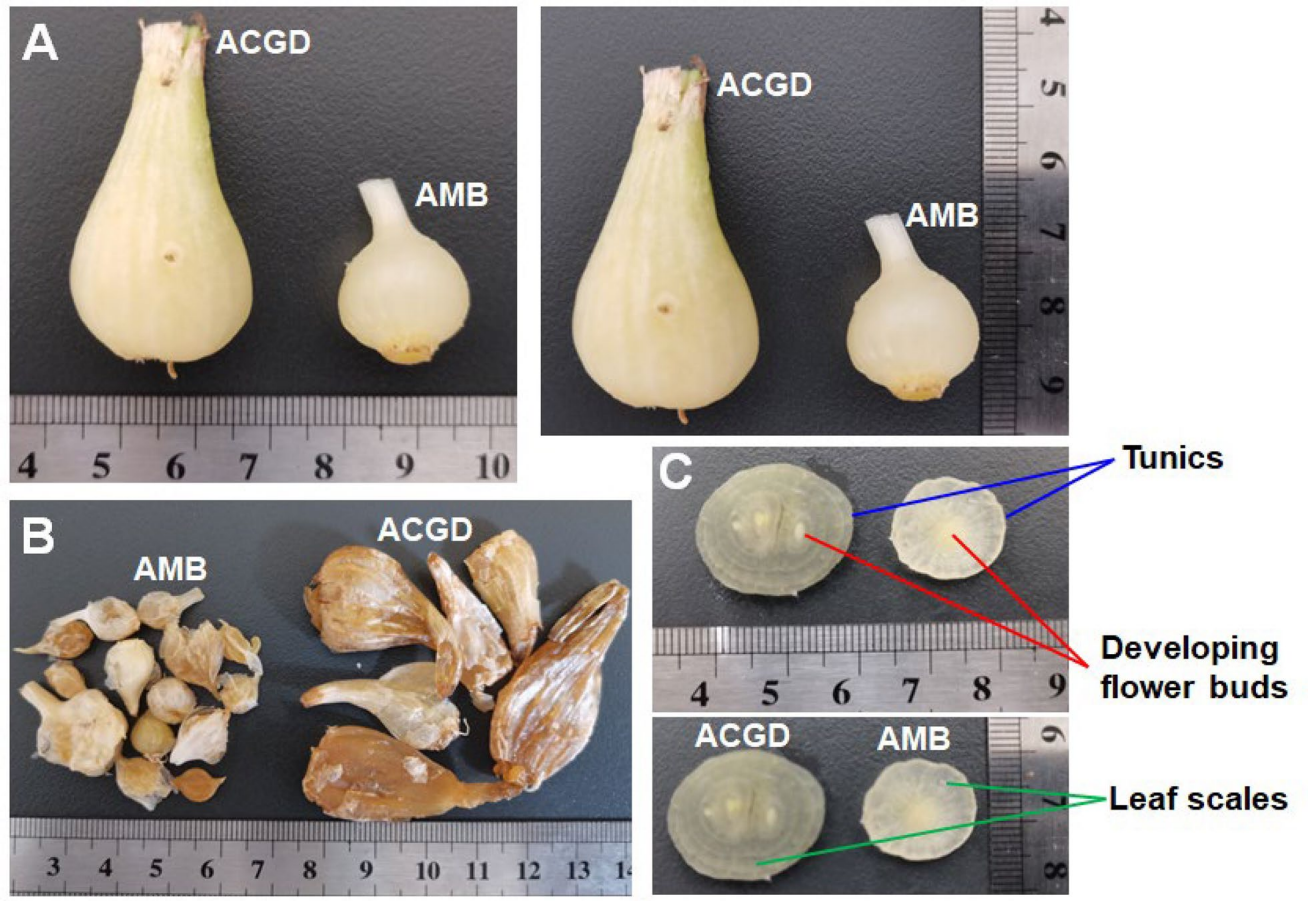
The morphology of two *Allium* varieties. **A** the appearance of fresh AMB and ACGD bulbs; **B** the appearance of dried AMB and ACGD bulbs; **C** the cross sections of two fresh *Allium* bulbs

Traditionally, there are two botanical origins of Xiebai including *Allium macrostemon* Bunge (AMB) and *Allium chinense* G. Don (ACGD) (Fig. 1). At present, about 600 *Allium* species distributed worldwide. Most of them are either important economic crops (onion, garlic, etc.) in market, or medicinal herbs widely used in pharmaceutical factory [7, 8]. There are fewer *Allium* species which are homology of medicine and food. But AMB and ACGD are an exception as well as garlic [4, 5, 7]. AMB is extensively located in China, Korea, Japan, and the Russian Far East [9]. Currently, AMB is usually processed into medicinal materials for Xiebai with the consumption of about 5000 tons per year [3]. In addition, ACGD is mainly produced in China, and it is cultivated around the world [10, 11]. Traditionally, ACGD is processed into canned food in markets, or exported to various parts of the world. Its annual production are approximately 300,000 tones [3]. Their medicinal and nutritional benefits had attracted increasing attention on their active components and pharmacological effects.

Previously, extensive studies have reported the secondary metabolites in these two *Allium* species [4, 5, 12–16]. For example, some volatile sulfur-containing components including methyl propyl disulfide, dimethyl disulfide, methyl allyl trisulfide, are regarded as the common active compounds in AMB and ACGD [15, 16]. In addition, they share a small portion of same saponins and great majority of different steroidal saponins [4, 5]. A wide variety of sapogenins (sarsasapogenin, smilagenin, tigogenin, etc.) were observed in AMB, while laxogenin in ACGD was the representative sapogenin [4–6, 14]. Besides, there are significant differences about the content levels and anti-platelet aggregation activities of these saponins [4–6, 13, 17]. In the contrast, except some free amino acids [3–5], chemical profiles and content evaluation of other primary metabolites in AMB and ACGD still remain unknown. Traditionally, the composition and content levels of fatty acids, amino acids, carotenoids, vitamins, and micronutrients are the reliable prediction indicators of food nutrition, aroma, and taste [18]. Therefore, we aimed to perform the holistic content comparison of five categories of nutrients and their derivatives in AMB and ACGD.

Recently, matrix-assisted laser desorption/ionization time-of-flight tandem mass spectrometry imaging analysis (MALDI-TOF MSI) has been applied to visualize the spatial location of target components in different tissue positions [6, 19]. As described previously, steroidal saponins are extensively detected in outside leaf scale and tunics of fresh AMB bulbs. Steroidal saponins are mostly observed in the whole leaf scale and tunics of ACGD bulbs, and partly in its developing flower buds [6, 20]. This detection pattern benefits from the MALDI-TOF MSI-guided in situ detection [6, 19]. These secondary metabolites were biosynthesized by a series of enzymatic reactions of primary metabolites. However, the visual spatial location information of primary metabolites including fatty acids, amino acids, carotenoids, and vitamins remain clear so far. It is necessary to obtain the spatial location information of primary metabolites in these two *Allium* species.

For these goals, first, this study evaluated the protective effect of myocardial injury of AMB and ACGD extracts on H9c2 cells. Further, the spatial distribution images of some main amino acids, fatty acids, carotenoids, and vitamins in AMB and ACGD samples was presented by MALDI-TOF MSI. Moreover, these metabolites were simultaneously quantified by multiple conventional LC–MS and ICP-MS analysis. Taken together, the results would be helpful to visualize the spatial accumulation of nutrients in these two species. It also proved that MALDI-TOF MSI analysis is a practical and viable tool to provide microscopic distribution analysis of biosynthesis, transportation, and accumulation of metabolites.

## Materials and methods

### Materials and chemicals

*Allium macrostemon* Bunge (AMB) fresh bulbs were collected from Shenyang (Liaoning, China), while *Allium chinense* G. Don (ACGD) fresh bulbs were obtained from Xinjian (Jiangxi, China). These two *Allium* species were identified by Prof. Xiaojian Zhang. In addition, the surfaces of these two *Allium* herbs were cleaned thoroughly, and kept at − 80 °C before use.

All reference standards including amino acids (l-cystine, *N*-propionylglycine, glycine, l-alanine, l-valine, etc.), fatty acids (hexanoic acid, eicosapentaenoic acid, linoleic acid, linolenic acid, etc.), carotenoids (α-carotene, etc.), vitamins (vitamin A, C, D, E, K1, K2, etc.), formic acid, dexrazoxane (Dex), methyl-thiazolyldiphenyl-tetrazolium bromide (MTT), lithium trifluoroacetic acid (LiTFA), 2,5-dihydroxybenzoic acid (DHB) with purities over 98.0% were obtained from Sigma-Aldrich (St. Louis, Missouri, USA). Butylated hydroxytoluene (BHT) was obtained from Aladdin (Shanghai, China). HPLC-grade *n*-hexane and methyl tert-butyl ether (MTBE) were purchased from Merck (Darmstadt, Germany). LC–MS grade acetonitrile, methanol, and water were provided from Fisher Scientific (Fair Lawn, NJ).

### Methyl thiazolyl tetrazolium assays

MTT experiments were referred to previous report [21]. In brief, the cells were seeded into 96-well plates. They were incubated for 24 h. After AMB extracts (or ACGD extracts) at different concentrations (31.25, 62.5, 125.0, 250.0, 500.0, 1000.0, and 2000.0 µg/mL) were incubated with the cells, MTT solution at 5 mg/mL (20 μL) was supplemented to each well and co-incubation for 4 h. Then, the supernatant was removed. DMSO solution (150 μL) was supplied into each well. The cell viability was measured at 490 nm by a microplate reader (BIO-RAD680, Bio-Rad, USA).

### Cell culture

DMEM containing 10% fetal bovine serum, 0.1% (v/v) penicillin, and 0.1% (v/v) streptomycin was used to culture H9c2 cells in a conventional incubator with 5% CO_2_ and 37 ºC. The recognized H/R model in vitro was established and validated to simulate the cardiac I/R injury in vivo [21]. When the cells were at a density of 7 × 10^4^/mL, they were seeded into 96-well plates and incubated for 24 h. When the cells arrived at the density of 80%, the DMEM medium was removed. Subsequently, the freshly prepared DMEM medium containing AMB extracts (or ACGD extracts) at concentrations of 500, 250, 125, 62.5, 31.25, 15.6 and 7.8 μg/mL was supplemented for 3 h. Then, these cells were put into a tri-gas incubator with N_2_/CO_2_ ratios of 95%:5% for 12 h. Finally, they were returned to standard incubator for 4 h.

### Biomarkers levels of myocardial injury

Traditionally, lactate dehydrogenase (LDH) and creatine kinase muscle-brain fraction (CK-MB) activities were measured to evaluate the levels of myocardial injury. After the cells were treated with a series of different concentrations of AMB extracts (or ACGD extracts), the collected supernatant (100 μL) was used to obtain the LDH and CK-MB levels with a COBAS INTEGRA 400 plus automatic biochemical analyzer (Roche, Switzerland).

### Quantification of cellular apoptosis levels

The percents of apoptotic cells in H9c2 cells were determined by the annexinV-FLUOS Kit [22]. Briefly, when the density of H9c2 cells was about 2 × 10^5^, they were seeded into a 6-well plate. Subsequently, these cells were incubated with serial concentrations of AMB extracts (or ACGD extracts) as described above. Then, the treated cells above were transferred into new EP tubes. They were slightly washed with PBS solution twice. Propidium iodide (PI) and annexin V were added and incubated with H9c2 cells for 15 min in the dark. Finally, the number of H9c2 cells which undergo the apoptosis process were read and quantified using a CytoFLEX flow cytometer (Beckman Coulter, USA).

### Western blotting method

Western blotting analysis were carried out as described previously [23]. In brief, the collected H9c2 cell lysates were pretreated with a bovine serum albumin kit for protein concentrations. The lysates were then kept in a metal bath at 90 °C for 10 min. The denaturized proteins (30 μg) were loaded on SDS gel electrophoresis on 12% gels. After efficient separation, the proteins were collected from the gel to membrane. The membrane was blocked for 1 h in a mixture including PBS solution, 0.1% Tween 20, and 5% skim milk. The membranes were further co-incubated with primary antibodies against BCL-2 (1:1000), and BAX (1:1000). The treated membranes were washed, and further co-incubated with corresponding secondary antibodies (1:5000) at room temperature. The target protein bands in membranes were captured and quantified using Image J software program.

### Sample preparation for quantification

First, freeze-dried AMB and ACGD samples were crushed to powder. For the quantification of free amino acids, the sample (50 mg) was dissolved with pre-cooled (− 20 °C) 70% methanol solution (0.5 mL). The mixture was first vortexed for 3 min, and centrifugated at 12,000 rpm for 10 min. The supernatant (300 μL) was followed a centrifugation (12,000 rpm, 10 min, 4 °C). The supernatant (200 μL) was treated with protein precipitation plate (Thermo Scientific Pierce) for UHPLC-MS/MS analysis.

As for free fatty acids, AMB or ACGD samples (50 mg) were treated with a mixed solution containing methanol (150 µL), MTBE (200 μL), and 36% precooled phosphoric acid (50 μL). The mixtures were vortexed (2500 rpm, 3 min), and centrifuged with 12,000 rpm for 5 min at 4 °C. Free fatty acids were dissolved in the upper organic solvents. The supernatant (200 μL) was taken and concentrated to dry under nitrogen. The residue was dissolved in 300 μL of 15% boron trifluoride-methanol solution (v/v), vortexed for 3 min, and kept in the oven at 60 °C for 0.5 h. The samples were treated with n-hexane (500 μL) and saturated sodium chloride solution (200 μL). After vertexing (2500 rpm, 3 min) and centrifugation (12,000 rpm, 5 min, 4 °C), the n-hexane layer supernatant (100 μL) was transferred for GC–MS analysis.

As for carotenoids, AMB or ACGD powder (50 mg) was dissolved and treated with a mixed solution. The solution mainly includes acetone, ethanol, and n-hexane with the ratio of 1:1:1 (v/v/v). It also contains 0.01% BHT. The extracts were vortexed (20 min), and centrifuged at 4 °C with 12,000 rpm for 5 min. The supernatant was concentrated to dry under nitrogen. The residue was reconstituted in 100 μL of MTBE-methanol solution (1:1, v/v). Then, the solution was injected for UHPLC-MS/MS analysis.

As for fat-soluble vitamins, AMB or ACGD samples (50 mg) was dissolved in 0.5 mL of mixed solution containing isopropanol: dichloromethane: methanol (1:1:8, v/v/v) and treated by ultrasonic dissolution for 20 min. After centrifugation, the supernatant (200 μL) was taken and mixed with n-hexane (800 μL). After vertexing at 2500 rpm for 3 min, the n-hexane layer was concentrated to dry under nitrogen. The dried residue was reconstituted in MTBE-methanol solution (100 μL) for further UHPLC-MS/MS analysis. For water-soluble vitamins, AMB or ACGD powder (50 mg) was dissolved and extracted in 0.1 mol/L HCl solution (0.5 mL). After centrifugation, the supernatant was collected for further UHPLC-MS/MS analysis.

As for micronutrients, AMB or ACGD samples (50 mg) was placed in PTFE digestion tube. A mixed solution (0.5 mL) including concentrated nitric acid and perchloric acid with the ratio of 4:1 (v/v) was added to the tube. The tube was heated from 25 to 100 °C within 10 min. It kept at 100 °C for 20 min. Then, the tube was cooled from 100 to 25 °C. The extracts were diluted to 5 mL with ultrapure water. The solution was centrifuged, and the collected filtrate was injected to ICP-MS analysis.

### Analytical conditions for the quantification of amino acids and their derivatives

The samples were separated using an UHPLC-ESI-MS/MS system (Exion LC AD, QTRAP 6500+ system). Waters ACQUITY BEH Amide chromatographic column (2.1 × 100 mm, 1.7 μm); column temperature was 40 °C; Water (A) and acetonitrile (B) both containing ammonium acetate (2 mM) and 0.04% formic acid; flow rate was 0.4 mL/min; The gradient program started at 90% B (0–1.2 min), decreased to 60% B (1.2–9.0 min), 40% B (9.0–11.0 min), finally ramped back to 90% B (11.0–15.0 min); injection volume was 2 μL.

AB 6500+ QTRAP LC–MS/MS system was equipped with an ESI ion source in both positive and negative ion modes. All collected data were processed by Analyst 1.6 software (AB SCIEX). Ion source: turbo spray; source temperature: 550 °C; ion spray voltage: 5.5 kV (positive) and − 4.5 kV (negative); curtain gas: 35.0 psi. De-clustering potentials (DP) and collision energies (CE) for individual multiple reaction monitoring (MRM) transitions was done with further DP and CE optimization.

### Analytical conditions for the quantification of carotenoids and their derivatives

The sample were carried out using an UHPLC-APCI-MS/MS system (Exion LC AD, Applied Biosystems 6500 Triple Quadrupole). YMC C30 column (100 mm × 2.0 mm, 3 μm); column temperature was 28 °C; solvent A, acetonitrile-methanol (3:1, v/v) containing 0.1% formic acid and 0.01% BHT; solvent B, methyl tert-butyl ether containing 0.01% BHT; gradient program started at 0% B from 0 to 3.0 min, 0–70% B from 3.0 to 5.0 min, 70%- 95% B from 5.0 to 9.0 min, finally ramped back to 0% B from 10.0 to 11.0 min; flow rate, 0.8 mL/min; injection volume, 2 μL.

QTRAP 6500+ LC–MS/MS system was performed in positive ion mode. All data was controlled by Analyst 1.6.3 software (AB SCIEX). The APCI source was equipped. Source temperature was 350 °C; curtain gas was 25.0 psi. DP and CE parameters were optimized. A series of optimum MRM transitions were monitored for each carotenoid and its derivatives.

### Analytical conditions for quantification of fat-soluble vitamins and their derivatives

The samples were separated on a waters HSS T3 column (2.1 mm × 50 mm, 1.8 µm) using Xevo TQ-XS/MS system equipped with electrospray ionization (ESI) mode (Waters, Manchester, UK). The column temperature was 50 °C. Mobile phase A and B were water and acetonitrile, respectively. They both contained 0.1% formic acid. Gradient program was as follows. 0–2.0 min, 70%-90%B; 2.0–2.5 min, 90%–100%B; 2.5–4.4 min, 100%B; 4.4–4.5 min, 100%–70%B; 4.5–6.0 min, 70%B. Flow rate, 0.4 mL/min. Injection volume, 10.0 μL. Source capillary voltage, 3.5 kV; cone voltage, 50 V. Desolvation temperature, 350 °C. Source desolvation flow, 650 L/h; cone gas flow, 50 L/h. The optimized cone and collision energy of each analyte were set to ensure the appropriate MRM transitions for the quantification of fat-soluble vitamins and their derivatives.

Similarly, Waters Xevo TQ-XS/MS system was used to perform the separation and quantification of water-soluble vitamins and their derivatives. Solvent A, water; Solvent B, acetonitrile; They both included 0.1% formic acid; Wates BEH C18 column (2.1 mm × 50 mm, 1.7 µm); 0–0.5 min, 95%B; 0.5–2.0 min, 95%–70%B; 2.0–3.0 min, 70%–40%B; 3.0–3.5 min, 40%–30%B; 3.5–3.7 min, 30%B; 3.7–4.0 min, 30%–95%B; 4.0–5.5 min, 95%B. flow rate: 0.5 mL/min; column temperature: 30 °C; injection volume: 5.0 μL; Source capillary voltage: 0.5 kV; cone voltage: 30 V; source desolvation flow were 800 L/h; cone gas flow: 100 L/h; desolvation temperature: 500 °C. Appropriate cone and collision energy of each MRM transition were set to quantify the water-soluble vitamins and their derivatives.

### Analytical conditions for quantification of fatty acids and their derivatives

The quantification of free fatty acids and their derivatives were performed using an GC-EI-MS system (Agilent 8890, 5977B system). Agilent DB-5MS capillary column (30 m × 0.25 mm × 0.25 μm); Carrier gas: high purity helium with purity over 99.999%; the heating procedure was started at 40 °C from 0 to 2.0 min, 30 °C/min increased to 200 °C from 2.0 to 3.0 min, 10 °C/min increased to 240 °C from 3.0 to 4.0 min, 5 °C/min increased to 285 °C from 4.0 to 7.0 min; traffic column flow: 1.0 mL/min; inlet temperature: 230 °C; injection volume: 1.0 μL.

The operation parameters for EI-MS were as follows. Ion source temperature was set at 230 °C; ionization voltage was 70 eV; transmission line temperature was at 240 °C; four-stage rod temperature was at 150 °C; solvent delay was set as 4 min; scanning mode was selective ion monitoring mode.

### Analytical conditions for quantification of trace elements

The analysis was performed in Hangzhou EXPECLIN PreMed 7000-ICP-MS equipped with Scott atomizing chamber (Hangzhou, China). Nebulizer gas flow, 0.6 L/min; collision gas flow, 0.88 L/min; auxiliary gas flow, 1.0 L/min; diluent gas flow, 0.73 L/min; atomizer flow, 0.1 mL/min; RF power, 1500 W; hexapole voltage, − 3.5 V; quadrupole voltage, − 0.61 V; counting voltage, 1720 V; extraction lens voltage, − 130 V; focusing lens voltage, 14.2 V; analog voltage, − 1550 V; Longitudinal, 0.6 mm; transverse, − 1.1 mm; depth, 5.5 mm; pump pipe diameter, 0.25 mm. The data was acquired in collision mode.

### Sample preparation for visual spatial distribution

First, 0.5% sodium carboxymethyl cellulose (CMC-Na) solutions were used as an embedding agent to fix AMB or ACGD fresh bulbs. The samples were crosscut at 20 ºC using Leica CM1950 cryostat to prepare 10 μm thickness section. Then, the section was transferred on electrically conductive slides. It was kept in a vacuum desiccator for 0.5 h. In addition, methanol and water was prepared a solution with the ratio of 9:1 (v/v). Besides, 0.1% LiTFA was added into the methanol–water solution. DHB was dissolved in this mixed solution at 15 mg/mL. Then, DHB solution was sprayed evenly on the slide. Flow rate was 0.1 mL/min; temperature was 75 ºC; pressure was set at 8 psi; 24 sprayed cycles; drying time was 10 s. The sprayed slides were used for MALDI-TOF/MSI analysis.

### Analytical conditions for visual imaging

MALDI-TOF MSI measurements was performed by tims TOF flex MS system (Bruker Daltonics, Bremen, Germany). A smart beam 3D laser was equipped at 10 kHz. In positive ion mode, the detection mass ranges for each analyte were set at *m/z* 200–1300 Da. Laser power was set as 80% in the whole experiment. The MSI spectrum of each ion contained about 500 laser shots. The resolution was about 100 μm. The MS/MS spectra by timsTOF flex MS system was obtained for identification and visual distribution comparison of metabolites in AMB and ACGD.

### Statistical analysis

The data were expressed as mean ± SD. The *t*-test was used to compare the mean values of AMB and ACGD groups. The values of *p* < 0.05 (*) were regarded significantly different.

## Results

### Morphology of the two *Allium* bulbs

The fresh bulbs of Xiebai have the best nutritional and medicinal values in the autumn based on the descriptions of ancient Chinese medical books. Therefore, AMB and ACGD bulbs were collected in September 2022 from Shenyang (Liaoning, China) and Xinjian (Jiangxi, China), respectively. As shown in Fig. 1A, B, there were obvious differences in appearances and the morphologies of fresh AMB and ACGD bulbs. On the whole, the size of AMB bulbs is 0.5–2.0 cm in diameter and 1.0–2.0 cm in length, whereas ACGD bulbs are with 1.0–3.0 cm in diameter and 2.0–4.0 cm in length. Further, AMB bulbs are ovoid or irregularly circular with pale yellowish-brown or externally yellowish-white, while ACGD bulbs are lightly compressed long-ovate with pale yellowish-brown or externally brown (Fig. 1A, B). From the perspective of slicing, they both consisted of inside developing flower buds, multiple leaf scales, and outside tunics (Fig. 1C).

### Myocardial injury protection effects of these two *Allium* extracts

**Fig. 2:**
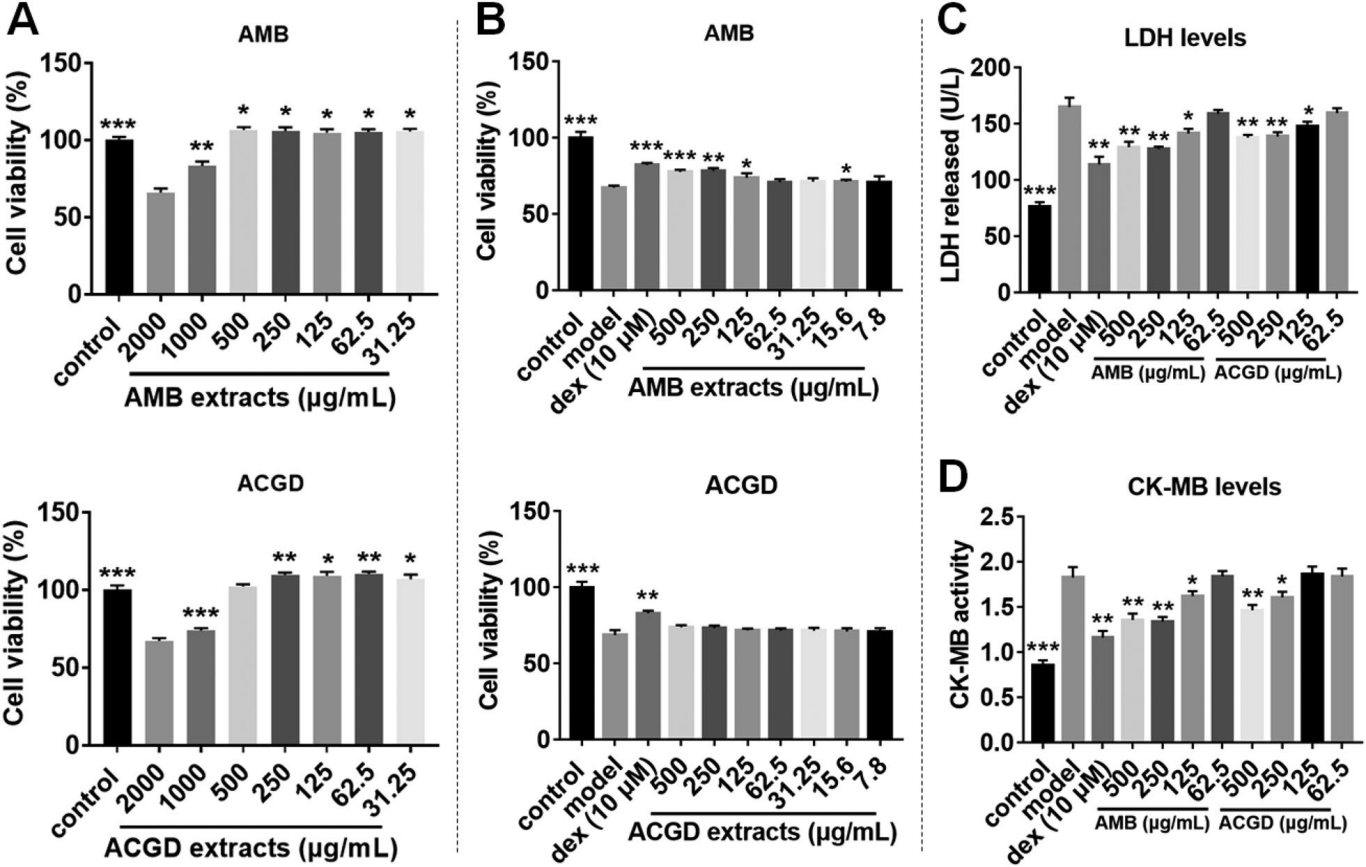
AMB and ACGD extracts affect the cell viability and attenuate H/R-induced H9c2 cell injury. **A** Cell viability measured using the MTT assay following treatment with serial concentrations of AMB and ACGD extracts; **B** effects of AMB and ACGD extracts on H/R-induced H9c2 cell viability; **C** effects of these two *Allium* extracts on the release of LDH levels; **D** effects of AMB and ACGD samples on the release of CK-MB levels. Data are presented as mean ± SD (n = 3). Dex is short for dexrazoxane. (* compared with model group, *p < 0.05, **p < 0.01, ***p < 0.001)

To determine the optimal concentrations of AMB and ACGD extracts for myocardial protection activity, MTT assays was used. As shown in Fig. 2A, the results demonstrated that treatment with these two extracts (31.25–500 μg/mL) both increased the cell viability on normal H9c2 cells (*p* < 0.05). After treatment with 2000 and 1000 μg/mL for AMB or ACGD extract, the cell viabilities both decreased, which suggested that AMB or ACGD extracts with over 1000 μg/mL are cytotoxic on H9c2 cells. Further, AMB extracts obviously elevated the cell viability compared with that in the H/R group (*p* < 0.05, Fig. 2B), while treatment with ACGD extracts did not bring obvious alterations of cell viability (*p* > 0.05, Fig. 2B). In addition, H/R modeling strongly induced LDH and CK-MB release, and these two species both could notably down-regulated the LDH and CK-MB levels. AMB extracts exhibited better down-regulation effects than those of ACGD samples (Fig. 2C, D).

**Fig. 3:**
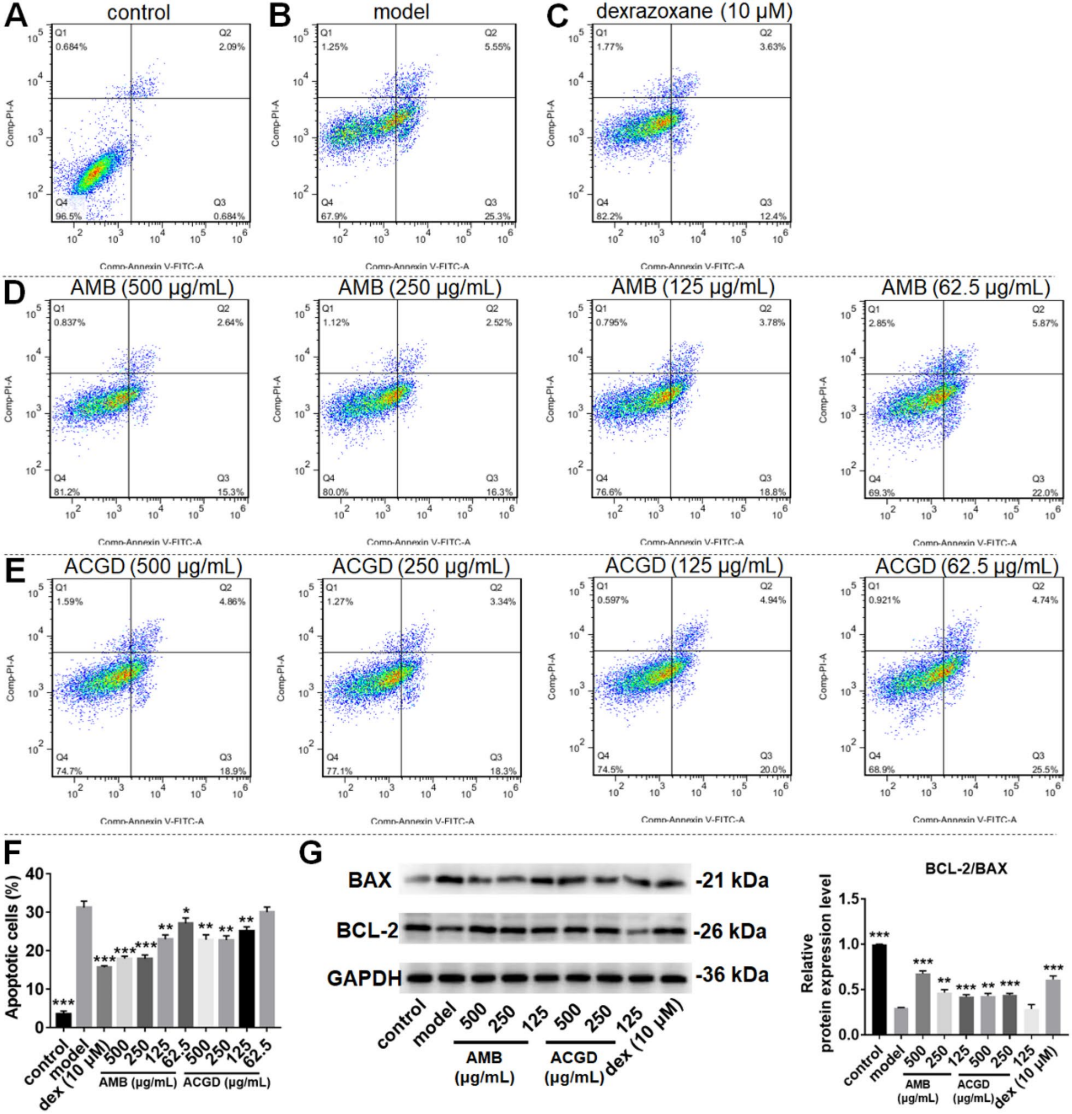
AMB and ACGD extracts protect H9c2 cells against H/R-induced apoptosis. Representative images of the total apoptotic cells stained by annexin V-FITC/PI in control group (**A**), model group (**B**), dexrazoxane group (**C**), AMB extracts group (**D**), and ACGD extracts group (**E**); **F** effects of 62.5, 125, 250, and 500 μg/mL AMB extracts and ACGD extracts on the apoptosis ratio of H9c2 cells; Q2 and Q3 represent late apoptosis and early apoptosis, respectively; **G** western blotting bands of Bcl-2, Bax and Gapdh, and ratios of Bcl-2/Bax from densitometric analyses of immunoblot images. Data are expressed as mean ± standard deviation (n = 3). Dex is short for dexrazoxane. (* compared with model group, *p < 0.05, **p < 0.01, ***p < 0.001)

To evaluate their anti-apoptotic effects, flow cytometry and protein expression assays were performed. In vitro H/R modeling (Fig. 3B) resulted in an about tenfold enhancement in apoptotic cells compared to control group (Fig. 3A). The apoptotic cells consisted of late and early apoptosis. Pre-treatment with dexrazoxane (Fig. 3C) significantly lowered the apoptotic ratio (*p* < 0.001). Similarly, the two *Allium* extracts both decreased the apoptotic ratio (Fig. 3D, E), especially AMB extracts (Fig. 3F). Moreover, the apoptotic proteins including BAX and BCL-2 were detected in Fig. 3G. H/R modeling decreased the BCL-2/BAX ratio. AMB and ACGD extracts both significantly improved the primary measured outcome (*p* < 0.05). These results suggested that these two *Allium* species can decline the cellular apoptosis levels induced by in vitro H/R modeling.

### Chemical characterization of primary metabolites

Chemical profiles of these two *Allium* species including 693 primary and secondary metabolites were characterized by UHPLC-TOF/MS and UHPLC/MS–MS as reported previously [6, 20]. The major differential metabolites are amino acids, fatty acids, carbohydrates, nucleotides, vitamins, and their derivatives [20]. Further, MALDI-TOF MSI-oriented visual spatial distribution for main secondary metabolites were displayed [6, 20].

In this study, MALDI-TOF MSI approach was carried out to perform visual spatial location characteristics of amino acids, fatty acids, carotenoids, and vitamins in these two *Allium* herbs (Table 1). As shown in Fig. 1C, many leaf scale orderly and alternately arranged between the developing flower buds and tunics. All leaf scales occupied over 90% of their whole transverse section.

**Table 1:** The detailed information of several main primary metabolites in fresh AMB and ACGD samples by MALDI timsTOF/MS and UHPLC-MS/MS analysis

No	Compounds	Formula	RT (min)	MALDI tims TOF MSI		UHPLC/MS–MS or GC–MS		Origin
				Ionization	Adduct ions	Q1 (Da)	Q3 (Da)	
*Amino acids*								
1	l-Alanine	C_3_H_7_NO_2_	8.29	268.1509	[3M+H]^+^	90.1	44.1	AMB/ACGD
2	l-Tyrosine	C_9_H_11_NO_3_	7.27	385.1376	[2M+Na]^+^	182.1	136.1	AMB/ACGD
3	l-Aspartate	C_4_H_7_NO_4_	9.97	289.0648	[2M+Na]^+^	134.0	74.0	AMB/ACGD
4	l-Histidine	C_6_H_9_N_3_O_2_	10.05	311.1468	[2M+H]^+^	156.1	110.1	AMB/ACGD
5	l-Lysine	C_6_H_14_N_2_O_2_	10.40	315.2008	[2M+Na]^+^	147.1	84.0	AMB/ACGD
6	l-Serine	C_3_H_7_NO_3_	9.10	338.1175	[2M+Na]^+^	106.0	60.0	AMB/ACGD
7	l-Tryptophan	C_11_H_12_N_2_O_2_	5.28	447.1435	[2M+K]^+^	205.1	118.1	AMB/ACGD
8	l-Arginine	C_6_H_14_N_4_O_2_	10.27	349.2312	[2M+H]^+^	175.1	70.1	AMB/ACGD
9	l-Threonine	C_4_H_9_NO_3_	8.54	277.0802	[2M+K]^+^	120.1	74.0	AMB/ACGD
10	l-Valine	C_5_H_11_NO_2_	7.00	273.1217	[2M+K]^+^	118.1	72.1	AMB/ACGD
11	l-Ornithine	C_5_H_12_N_2_O_2_	10.44	303.1435	[2M+K]^+^	133.1	70.0	AMB/ACGD
12	l-Citrulline	C_6_H_13_N_3_O_3_	9.40	373.1812	[2M+Na]^+^	176.1	113.0	AMB/ACGD
13	Argininosuccinic acid	C_10_H_18_N_4_O_6_	10.84	291.1305	[M+H]^+^	291.5	176.1	AMB/ACGD
14	Glutathione oxidized	C_20_H_32_N_6_O_12_S_2_	11.09	651.1157	[M+K]^+^	613.2	484.0	AMB/ACGD
15	γ-Glutamate-cysteine	C_8_H_14_N_2_O_5_S	9.86	273.0521	[M+Na]^+^	251.1	130.1	AMB/ACGD
16	l-Tryptophyl-l-glutamic acid	C_16_H_19_N_3_O_5_	8.39	372.0962	[M+K]^+^	334.0	159.0	AMB/ACGD
17	*N*ʹ-Formylkynurenine	C_11_H_12_N_2_O_4_	7.27	259.0695	[M+Na]^+^	237.1	146.1	AMB/ACGD
18	*N*-Acetylneuraminic acid	C_11_H_19_NO_9_	9.61	332.0958	[M+Na]^+^	310.1	274.1	AMB/ACGD
19	l-Asparagine anhydrous	C_4_H_8_N_2_O_3_	9.19	303.0707	[2M+K]^+^	133.1	74.0	AMB/ACGD
20	l-Glutamine	C_5_H_10_N_2_O_3_	9.05	331.1020	[2M+K]^+^	147.1	84.0	AMB/ACGD
21	l-Glutamic acid	C_5_H_9_NO_4_	9.48	295.1141	[2M+H]^+^	148.1	84.0	AMB/ACGD
22	Succinic acid	C_4_H_6_O_4_	1.71	237.0610	[2M+H]^+^	117.0	99.0	AMB/ACGD
23	γ-Aminobutyric acid	C_4_H_9_NO_2_	7.45	348.1537	[3M+K]^+^	104.1	68.8	AMB/ACGD
24	(5-l-Glutamyl)-l-amino acid	C_8_H_14_N_2_O_5_	9.89	475.1443	[2M+K]^+^	219.1	202.0	AMB/ACGD
*Fatty acids*								
25	α-Linolenic acid (α-LA)	C_18_H_30_O_2_	11.79	317.1883	[M+Na]^+^	292.2		AMB/ACGD
26	Cis-5,8,11,14,17-eicosa- -pentaenoic acid (EPA)	C_20_H_30_O_2_	13.20	341.1883	[M+K]^+^	316.3		AMB/ACGD
27	Cis-4,7,10,13,16,19-docosa- -hexaenoic acid (DHA)	C_22_H_32_O_2_	15.16	367.2039	[M+K]^+^	79.6		AMB/ACGD
28	Cis-7,10,13,16,19-docosa- pentaenoic acid (DPA)	C_22_H_34_O_2_	15.33	369.2196	[M+K]^+^	344.0		AMB/ACGD
29	Linoleic acid (LA)	C_18_H_32_O_2_	11.73	319.2039	[M+K]^+^	294.8		AMB/ACGD
30	Arachidonic acid (AHA)	C_20_H_32_O_2_	13.14	343.2039	[M+K]^+^	318.4		AMB/ACGD
31	Palmitic acid	C_16_H_32_O_2_	10.39	295.2039	[M+K]^+^	270.5		AMB/ACGD
32	Stearic acid	C_18_H_36_O_2_	11.99	323.2352	[M+K]^+^	298.3		AMB/ACGD
33	Arachidic acid (AA)	C_20_H_40_O_2_	13.82	351.2665	[M+K]^+^	326.9		AMB/ACGD
34	Behenic acid	C_22_H_44_O_2_	16.06	379.2978	[M+K]^+^	354.2		AMB/ACGD
35	Lignoceric acid	C_24_H_48_O_2_	18.50	407.3291	[M+K]^+^	382.5		AMB/ACGD
36	Cis-9-octadecenoic acid	C_18_H_34_O_2_	11.79	283.2637	[M+H]^+^	296.6		AMB/ACGD
*Carotenoids*								
37	β-carotene	C_40_H_56_	6.28	575.4019	[M+K]^+^	537.6	177.1	AMB/ACGD
38	Zeaxanthin	C_40_H_56_O_2_	4.65	607.3917	[M+K]^+^	569.4	477.5	AMB
39	lutein	C_40_H_56_O_2_	4.07			551.5	175.4	AMB/ACGD
40	Lutein dilaurate	C_64_H_101_O_4_	7.18	972.7337	[M+K]^+^	733.5	533.3	AMB/ACGD
41	Violaxanthin	C_40_H_56_O_4_	1.56	639.3816	[M+K]^+^	601.4	221.0	AMB/ACGD
42	Neoxanthin	C_40_H_56_O_4_	1.94			601.4	565.5	AMB/ACGD
43	Violaxanthin dilaurate	C_64_H_101_O_6_	6.62	966.7676	[M+H]^+^	966.7	948.8	AMB/ACGD
44	Violaxanthin–myristate–laurate	C_66_H_104_O_6_	6.85	1031.7470	[M+K]^+^	993.8	975.7	AMB/ACGD
45	Violaxanthin–myristate–caprate	C_64_H_100_O_6_	6.84	965.7598	[M+H]^+^	965.7	947.8	AMB/ACGD
46	Zeaxanthin dimyristate	C_68_H_108_O_4_	7.62	1011.8145	[M+Na]^+^	990.0	761.8	AMB
*Vitamins*								
47	Vitamin A	C_20_H_30_O	1.83	325.1934	[M+K]^+^	269.3	93.0	AMB/ACGD
48	Vitamin D2	C_28_H_44_O_2_	1.98	451.2978	[M+K]^+^	413.4	112.9	AMB/ACGD
49	Vitamin D3	C_27_H_44_O_2_	1.94	439.2978	[M+K]^+^	401.4	365.2	AMB/ACGD
50	Vitamin E	C_29_H_50_O_2_	3.02	431.3889	[M+H]^+^	431.3	165.2	AMB/ACGD
51	Vitamin B1 (thiamine)	C_12_H_17_ClN_4_OS	0.75	339.0449	[M+K]^+^	265.1	122.1	AMB/ACGD
52	Vitamin B2 (riboflavin)	C_17_H_20_N_4_O_6_	2.96	377.1461	[M+H]^+^	377.3	243.1	AMB/ACGD
53	Vitamin B3 (nicotinamide)	C_6_H_5_NO_2_	1.72	269.0538	[2M+Na]^+^	123.2	80.2	AMB/ACGD
54	Vitamin B5 (pantothenic acid)	C_9_H_17_NO_5_	1.96	477.1851	[2M+K]^+^	220.1	90.1	AMB/ACGD
55	Vitamin B6 (pyridoxic acid)	C_8_H_9_NO_4_	1.95	367.1141	[2M+H]^+^	183.9	147.9	AMB/ACGD
56	BDC (pyridoxine)	C_8_H_11_O_3_N	1.40	377.1115	[2M+K]^+^	184.0	148.0	AMB/ACGD
57	Vitamin B7 (biotin)	C_10_H_16_N_2_O_3_S	2.78	283.0519	[M+K]^+^	244.9	227.0	AMB/ACGD
58	Vitamin B9	C_19_H_19_N_7_O_6_	2.75	464.1295	[M+Na]^+^	442.0	295.1	AMB/ACGD
59	Vitamin B12 (cobalamin)	C_63_H_88_CoN_14_O_14_P	2.32	1355.5752	[M+H]^+^	117.0	73.0	AMB/ACGD
60	5-Methyltetrahydrofolate (5-MTHFA)	C_20_H_25_N_7_O_6_	2.25	498.1503	[M+K]^+^	460.2	313.1	AMB/ACGD
61	Vitamin C	C_6_H_8_O_6_	0.61	353.0720	[2M+H]^+^	175.0		AMB/ACGD

In addition, a total of 76 amino acids, 32 fatty acids, 12 carotenoids, 15 vitamins and 21 trace elements were simultaneously quantified by several conventional approaches including UHPLC/MS–MS, GC–MS and ICP-MS. Their multiple reaction monitoring (MRM) chromatograms were shown in Additional file 1: Fig. S1-S4. Herein, the visual spatial locations and absolute quantification of some primary metabolites from AMB and ACGD samples were deciphered for the first time.

### Visual spatial distribution and absolute quantification of amino acids

**Fig. 4:**
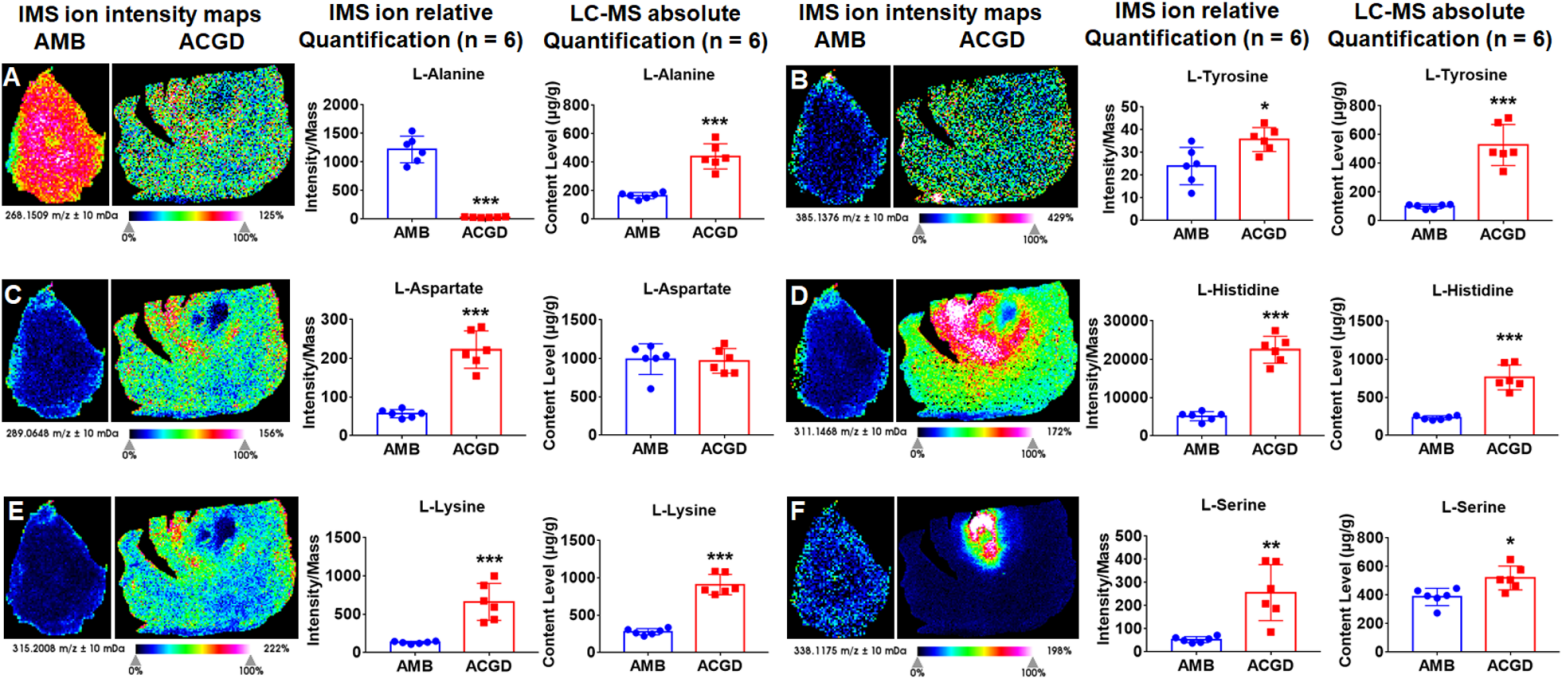
MALDI-TOF IMS and quantitative LC–MS analysis of six main amino acids in fresh AMB and ACGD bulbs. **A** l-Alanine at *m/z* 268.1509 ([3M+H]^+^); **B** l-Tyrosine at *m/z* 385.1376 ([2M+Na]^+^); **C** l-Aspartate at *m/z* 289.0648 ([2M+Na]^+^); **D** l-Histidine at *m/z* 311.1468 ([2M+H]^+^); **E** l-Lysine at *m/z* 315.2008 ([2M+Na]^+^); **F** l-Serine at *m/z* 338.1175 ([2M+Na]^+^); Each row presents the respective ion images, as well as relative quantification (intensity) extracted from the IMS measurements and absolute quantification (μg/g) determined by LC–MS approaches. The scale is 1.0 mm. (* compared with AMB group, *p < 0.05, **p < 0.01, ***p < 0.001)

Amino acids and their derivatives are one important category of primary metabolites in AMB and ACGD herbs. Their quantitative analysis by UHPLC/MS–MS was displayed in Additional file 1: Tables S1-S2. In addition, they were usually observed with [M+H]^+^, [M+Na]^+^, [M+K]^+^ species, and their dimer or polymer ions. For example, l-tyrosine, l-aspartate, l-lysine and l-serine were detected with [2M+Na]^+^ ions at *m/z* 385.1376, 289.0648, 315.2008 and 338.1175, respectively (Fig. 4). l-histidine was observed at *m/z* 311.1468 ([2M+H]^+^), while l-alanine was visualized at *m/z* 268.1509 ([3M+H]^+^) (Fig. 4). For AMB, l-alanine was distributed in whole bulbs, especially developing flower buds and inside leaf scales (Fig. 4A). For ACGD, l-serine was almost located in the developing flower buds (Fig. 4F), while the inside leaf scale was the most abundant area for l-histidine (Fig. 4D). Except l-aspartate, other amino acids exhibited similar distribution trends as well as their absolute quantitative results (Fig. 4).

Besides, oxidized glutathione, l-citrulline, l-tryptophan, l-glutamic acid, and l-glutamine were top5 abundant amino acids (over 2000 μg/g). They accounted for about 63.4% and 77.9% of total amino acids in AMB and ACGD bulbs, respectively (Additional file 1: Table S2). l-tryptophan was observed with [2M+K]^+^ ions at *m/z* 447.1435. It was visualized at AMB outside tunics, and ACGD inside leaf scales (Additional file 1: Fig. S5A). Similarly, l-glutamine gave the [2M+K]^+^ ions at *m/z* 331.1020 (Additional file 1: Fig. S7B). Oxidized glutathione were presented based on its [M+K]^+^ ion at *m/z* 651.1157. It was mainly distributed in AMB outside tunics and whole ACGD bulbs (Additional file 1: Fig. S6B). l-citrulline ([2M+Na]^+^, *m/z* 373.1812, Additional file 1: Fig. S5F) was rich in whole AMB and ACGD bulbs except developing flower buds. l-glutamic acid ([2M+H]^+^, *m/z* 295.1141) was abundant in ACGD inside leaf scales and sporadically in AMB outside tunics (Additional file 1: Fig. S7C). The content levels of l-tryptophan, l-glutamine, oxidized glutathione, and l-glutamic acid kept in line with their MSI intensity, whereas that of l-citrulline showed an opposite trend.

Except these five main components, other amino acids and their derivatives including l-alanine, l-lysine, l-tyrosine, γ-glutamate-cysteine, (5-l-glutamyl)-l-amino acid, 3-aminoisobutanoic acid, and γ-aminobutyric acid, were over 100 μg/g. They were also the important nutrients in these two *Allium* vegetables. For instance, argininosuccinic acid ([M+H]^+^, *m/z* 291.1305, Additional file 1: Fig. S6A) and *N*ʹ-formylkynurenine ([M+Na]^+^, *m/z* 259.0695, Additional file 1: Fig. S6E) were both abundant in ACGD tunics and whole leaf scales, and AMB tunics. Visual MSI images showed that γ-aminobutyric acid was more abundant in AMB than ACGD, its absolute content in ACGD were actually higher than that in AMB (Additional file 1: Fig. S7E). For most primary metabolites, the relative quantitative results demonstrated a consistent trend with the absolute content levels. The inconsistent results may be attributed to the disadvantages of MALDI-TOF MSI-based in-situ detections.

### Spatial analysis and simultaneous quantification of fatty acids

Based on the quantitative results (Additional file 1: Tables S3, S4), saturated fatty acids shared 60.3% and 63.7% of total fatty acids in AMB and ACGD bulbs, respectively. Among them, the content levels of stearic acid and palmitic acid were both over 196.7 μg/g in these two herbs. They both exhibited the visual MSI images with [M+K]^+^ ion at *m/z* 295.2039 and 323.2352, respectively (Additional file 1: Fig. S8A, B). The images showed these two ions were sporadically distributed in AMB tunics and whole ACGD bulbs. Quantitative results displayed that the content level of palmitic acid was close between these two herbs, and stearic acid level was more abundant in AMB than ACGD. Further, other saturated fatty acids shared similar spatial distribution patterns with palmitic acid and stearic acid (Additional file 1: Fig. S8).

**Fig. 5:**
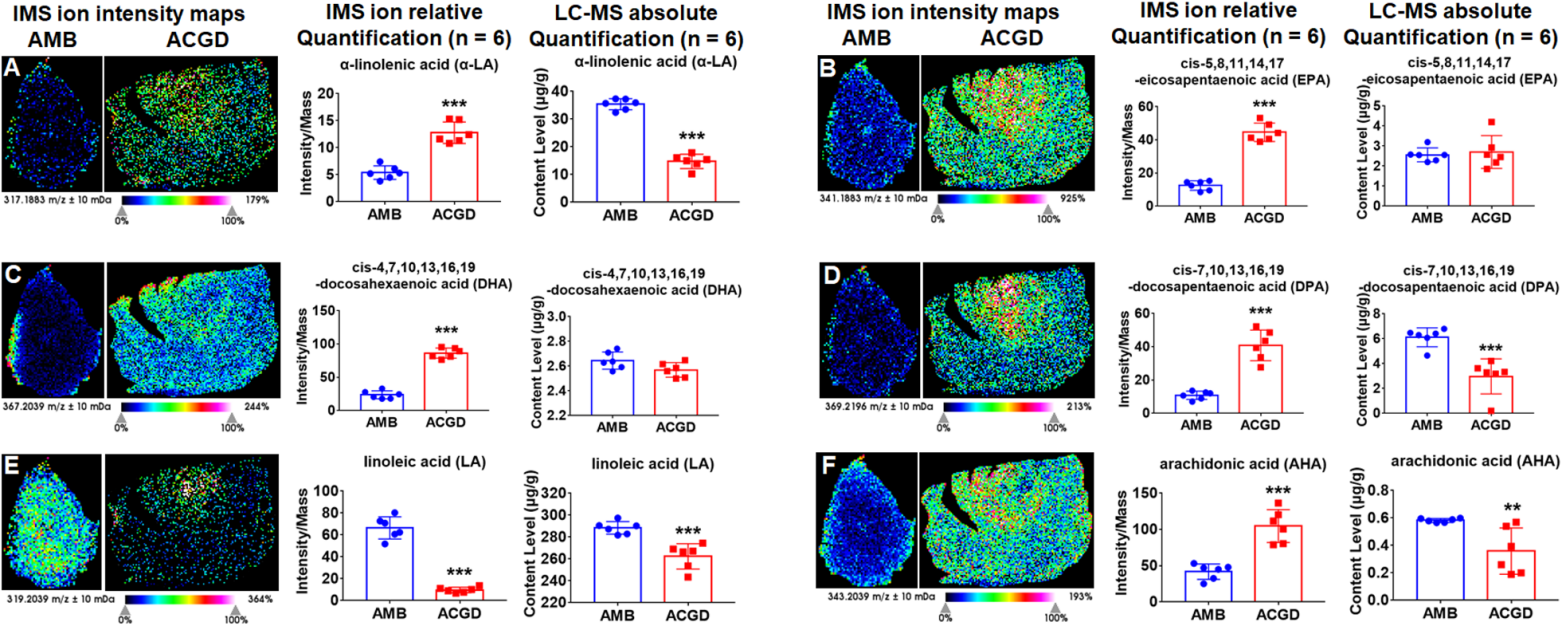
MALDI-TOF IMS and quantitative LC–MS analysis of several polyunsaturated fatty acids (PUFAs). **A** α-linolenic acid (α-LA) at *m/z* 317.1883 ([M+Na]^+^); **B** cis-5,8,11,14,17-eicosapentaenoic acid (EPA) at *m/z* 341.1883 ([M+K]^+^); **C** cis-4,7,10,13,16,19-docosahexaenoic acid (DHA) at *m/z* 367.2039 ([M+K]^+^); **D** cis-7,10,13,16,19-docosapentaenoic acid (DPA) at *m/z* 369.2196 ([M+K]^+^); **E** linoleic acid (LA) at *m/z* 319.2039 ([M+K]^+^); **F** arachidonic acid (AHA) at *m/z* 343.2039 ([M+K]^+^); Each independent row presents the visual IMS images, relative quantification (intensity) derived from the IMS analysis, and absolute quantification (μg/g) determined by LC–MS approaches. The scale is 1.0 mm. (* compared with AMB group, *p < 0.05, **p < 0.01, ***p < 0.001)

Omega-3 and omega-6 unsaturated fatty acids are two prominent categories of unsaturated fatty acids. In this study, omega-3 fatty acids mainly included α-linolenic acid (α-LA), cis-4,7,10,13,16,19-docosahexaenoic acid (DHA), cis-5,8,11,14,17-eicosapentaenoic acid (EPA), and cis-7,10,13,16,19-docosapentaenoic acid (DPA). α-LA ([M+Na]^+^, *m/z* 317.1883, Fig. 5A) and DPA ([M+K]^+^, *m/z* 369.2196, Fig. 5D) were more abundant in AMB than ACGD. They were mostly observed in AMB tunics and whole ACGD bulbs. DHA and DPA exhibited similar visual MSI images and close content levels in these two species (Fig. 5B, C). In addition, LA ([M+K]^+^, *m/z* 319.2039), a main omega-6 fatty acid (over 200 μg/g), was located in whole AMB bulbs, and sporadically distributed in ACGD bulbs. Its content levels supported this finding (Fig. 5E). AHA ([M+K]^+^, *m/z* 343.2039) presented a contrary trend between MSI intensity and content levels (Fig. 5F).

### Visualization location and quantification of carotenoids

**Fig. 6:**
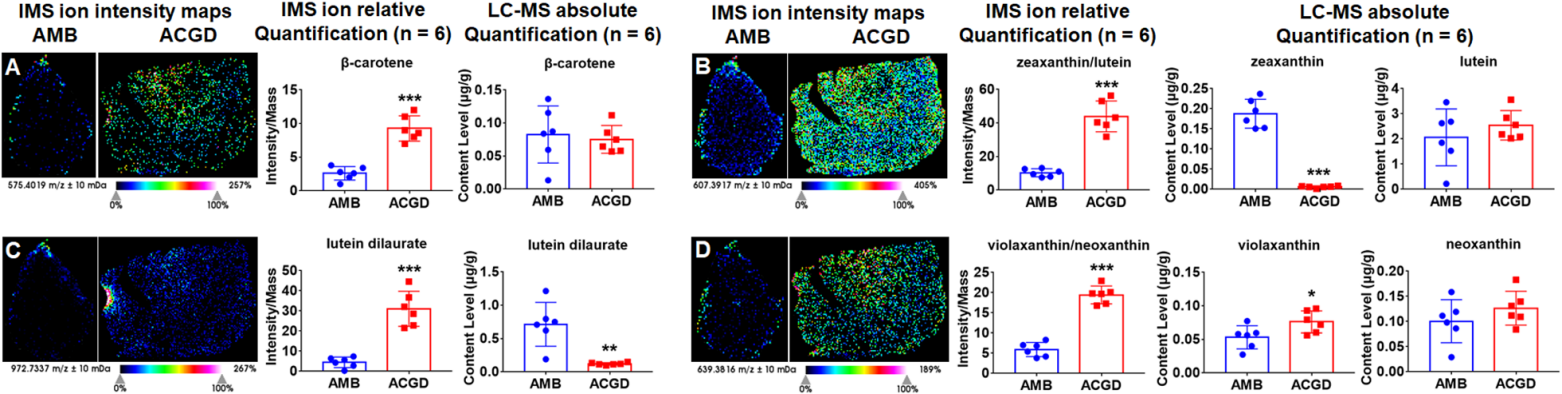
MALDI-TOF IMS and quantitative LC–MS analysis of four major carotenoids and their derivates. **A** β-carotene at *m/z* 575.4019 ([M+K]^+^); **B** zeaxanthin and lutein at *m/z* 607.3917 ([M+K]^+^); **C** Lutein dilaurate at *m/z* 972.7337 ([M+K]^+^); **D** violaxanthin and neoxanthin at *m/z* 639.3816 ([M+K]^+^); the visual IMS images, relative quantification (intensity) by MALDI-TOF IMS approach, and absolute quantification (μg/g) determined by LC–MS analysis are presented at each independent. The scale is 1.0 mm. (* compared with AMB group, *p < 0.05, **p < 0.01, ***p < 0.001)

The quantitative results showed that carotenoids were a category of trace components in AMB and ACGD samples (Additional file 1: Tables S5, S6). Except lutein over 2.0 μg/g, other carotenoids and their derivatives were all less than 1.0 μg/g. Actually, a total of 12 carotenoids was simultaneously quantified. The relative quantitative levels of other 20 derivatives were obtained based on the calibration curves of their aglycones (Additional file 1: Table S5). β-carotene ([M+K]^+^, *m/z* 575.4019) exhibited close absolute contents in AMB and ACGD (Fig. 6A). Lutein and zeaxanthin were a pair of isomers with same [M+K]^+^ ion at *m/z* 607.3917 and MSI images (Fig. 6B). Similar, the ionization characterization ([M+K]^+^, *m/z* 639.3816) and visual MSI images of violaxanthin and neoxanthin were same (Fig. 6D). These carotenoids were all mainly distributed in AMB outside tunics and whole ACGD bulbs.

Their derivatives shared the same visual spatial patterns as well as their aglycones (Additional file 1: Fig. S9). Meanwhile, their MSI images showed that lutein dilaurate ([M+K]^+^, *m/z* 972.7337, Fig. 6C), violaxanthin dilaurate ([M+H]^+^, *m/z* 966.7676, Additional file 1: Fig. S9A), violaxanthin–myristate–laurate ([M+K]^+^, *m/z* 1031.7470, Additional file 1: Fig. S9B), violaxanthin–myristate–caprate ([M+H]^+^, *m/z* 965.7598, Additional file 1: Fig. S9C), zeaxanthin dimyristate ([M+Na]^+^, *m/z* 1011.8145, Additional file 1: Fig. S9D) were richer in ACGD than AMB bulbs. However, relative quantification results indicated these components were more abundant in AMB than ACGD.

### Spatial distribution and quantification of vitamins

**Fig. 7:**
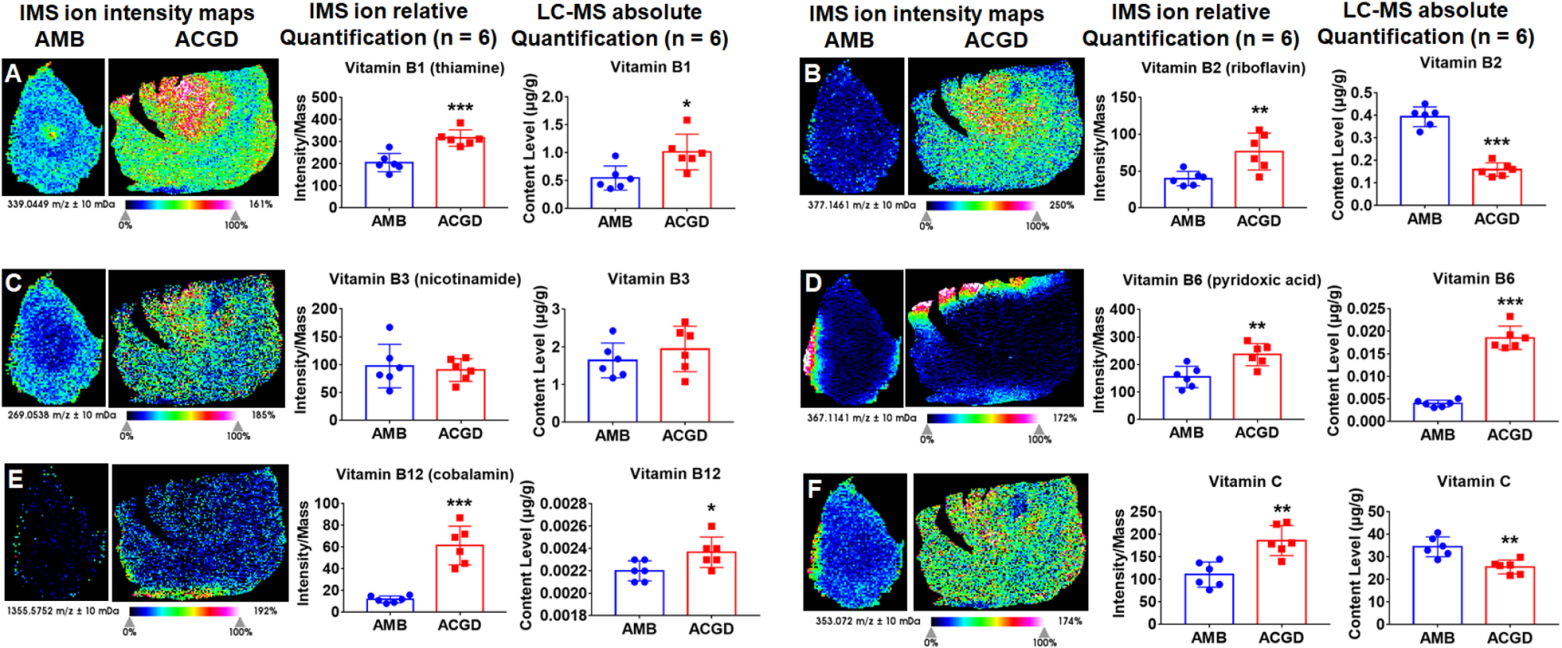
MALDI-TOF IMS and quantitative LC–MS analysis of several water-soluble vitamins. **A** vitamin B1 (thiamine) at *m/z* 339.0449 ([M+K]^+^); **B** vitamin B2 (riboflavin) at *m/z* 377.1461 ([M+H]^+^); **C** vitamin B3 (nicotinamide) at *m/z* 269.0538 ([2M+Na]^+^); **D** vitamin B6 (pyridoxic acid) at *m/z* 367.1141 ([2M+H]^+^); **E** vitamin B12 (cobalamin) at *m/z* 1355.5752 ([M+H]^+^); **F** vitamin C at *m/z* 353.0720 ([2M+H]^+^); Each row presents the visual IMS images, relative quantification (intensity) by IMS analysis, and absolute quantification (μg/g) by LC–MS approaches. The scale is 1.0 mm. (* compared with AMB group, *p < 0.05, **p < 0.01, ***p < 0.001)

The quantification of 11 water-soluble vitamins and 4 fat-soluble vitamins were performed by UHPLC/MS–MS (Additional file 1: Tables S7, S8). As shown in Fig. 7A, vitamin B1 ([M+K]^+^, *m/z* 339.0449) was mainly located in AMB developing flower buds and outside tunics, while it was also abundant in ACGD developing flower buds, and gradually decreased from inside leaf scales to outside leaf scales. MALDI TOF-based intensity and absolute quantification results both proved it was richer in ACGD than AMB. Vitamin B2 (M+H]^+^, *m/z* 377.1461, Fig. 7B), vitamin B3 ([2M+Na]^+^, *m/z* 269.0538, Fig. 7C) and vitamin C ([2M+H]^+^, *m/z* 353.0720, Fig. 7F) exhibited same or similar spatial distribution patterns with that of vitamin B1. The content levels of vitamin B2 and vitamin C in AMB were both more abundant than those in ACGD. On the contrary, vitamin B6 (Fig. 7D) and vitamin B12 (Fig. 7E) were lower in AMB than ACGD. Besides, the contents of vitamin B5, pyridoxine, and vitamin B7 were all lower in ACGD than those in AMB (Additional file 1: Figs. S10A–11C).

For fat-soluble vitamins, vitamin A (over 0.2 μg/g) was far higher than vitamins D2, D3 and E (Additional file 1: Fig. S11). Vitamin A ([M+K]^+^, *m/z* 325.1934, Additional file 1: Fig. S11A) was mainly visualized in AMB tunics and whole ACGD bulbs. However, vitamin D2 ([M+K]^+^, *m/z* 451.2978, Additional file 1: Fig. S11B), vitamin D3 ([M+K]^+^, *m/z* 439.2978, Additional file 1: Fig. S11C), vitamin E ([M+H]^+^, *m/z* 431.3889, Additional file 1: Fig. S11D) were sporadically located in AMB tunics, and ACGD bulbs. The absolute quantitative analysis suggested that there were no significant differences between these two *Allium* species.

### Comparative analysis of content levels of trace elements

A total of 21 trace elements were exactly quantified by ICP-MS (Additional file 1: Tables S9-S10). As shown in Additional file 1: Fig. S12, calcium (Ca) and magnesium (Mg) were both over 9724.46 μg/g. In addition, iron (Fe), zinc (Zn), manganese (Mn), copper, and strontium (Sr) were all over 47.46 μg/g. Other trace elements were almost less than 10.0 μg/g. Obviously, the contents of most trace elements including Mg, Mn, Ni, Cu, Zn, As, Mo, Cd, Ba, Tl and Pb were higher in ACGD than those in AMB (Additional file 1: Fig. S12).

**Fig. 8:**
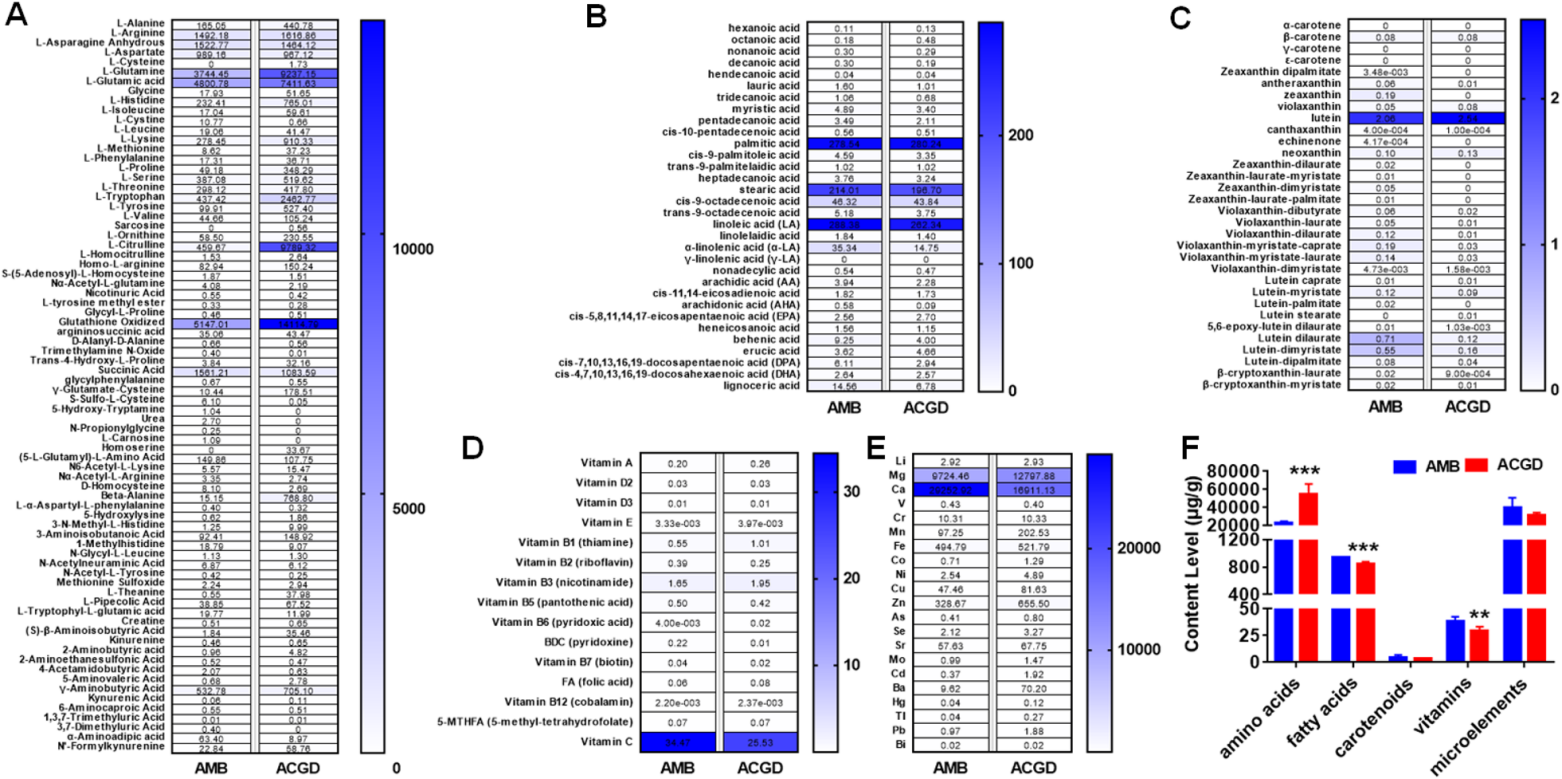
Comparison of absolute content levels (μg/g) of several main primary metabolites in fresh AMB and ACGD bulbs. **A** heatmaps of amino acids and their derivates; **B** heatmaps of fatty acids; **C** heatmaps of carotenoids and their derivates; **D** heatmaps of fat-soluble vitamins and water-soluble vitamins; **E** heatmaps of trace elements; **F** comparison of content levels of total amino acids, fatty acids, carotenoids, vitamins, and trace elements; The number in heatmaps present the mean content level of each primary metabolite in AMB and ACGD samples (n = 6). Content levels were normalized by row scale. (* compared with AMB group, *p < 0.05, **p < 0.01, ***p < 0.001)

Moreover, the heat-map directly presented the similarities and differences of content levels of these primary metabolites between AMB and ACGD bulbs (Fig. 8). As described above, l-glutamine, l-tryptophan, l-glutamic acid, l-citrulline, and oxidized glutathione were all over 2000 μg/g (Fig. 8A), and the contents of total amino acids were higher in ACGD than those in AMB (Fig. 8F). Palmitic acid, stearic acid and linolenic acid accounted for about 83.2% and 87.1% of total fatty acids in AMB and ACGD, respectively (Fig. 8B). Lutein, one of the most abundant carotenoids, occupied approximately 39.5% and 75.6% of total carotenoids (Fig. 8C). As shown in Fig. 8D, vitamin C accounted for about 90.2% and 86.1% of total vitamins in AMB and ACGD, respectively. Similarly, approximately 97.2% and 94.8% of total trace elements were Mg and Ca in AMB and ACGD, respectively (Fig. 8E). Taken together, the contents of total fatty acids and vitamins in ACGD were both lower compared with AMB. There were no remarkable differences among the total carotenoids and trace elements between these two *Allium* species (Fig. 8F).

## Discussion

Actually, AMB and ACGD herbs are both used for treating coronary heart disease in traditional medicinal use [4]. In the current study, one important finding was that comparative analysis of myocardial injury protection effects involving in apoptotic pathways of AMB and ACGD extracts were performed (Fig. 3). Previously, pretreatment with AMB extracts can lessen the acute myocardial ischemia injury, and further maintain the body metabolic balance. The mechanism may be that AMB extracts regulated the amino acid metabolism and limited the energy metabolism change [24]. Phenolic amides from ACGD bulbs could alleviate the damage of H9c2 cells induced by H_2_O_2_ in vitro [25]. In addition, some isolated compounds from AMB and ACGD extracts exerted pronounced anti-inflammatory and anti-proliferative activities [4, 14, 26]. Moreover, furostanol saponins from AMB can obviously inhibit ADP-induced platelet PI3K expression and Akt phosphorylation [27]. And A-24, a novel saponin from ACGD, could induce apoptosis and autophagy by PI3K/Akt/mTOR pathway [28]. Our findings enhanced the understanding of their benefits on myocardial injury protection.

Our results showed that AMB and ACGD herbs both demonstrated remarkable myocardial injury protection activity on H9c2 cells. These extracts contained primary and secondary metabolites. In our previous studies, volatile sulfur-containing compounds, nitrogen-containing compounds, and steroidal saponins are identified as the active compounds for these pharmacological activities in these two *Allium* herbs [4, 5]. This was because we had proved the volatile organic compounds and their extracts both demonstrated close anti-atherosclerosis effects and lipid lowering activity [20, 29]. Some steroidal saponins showed significant platelet inhibitory activity [6]. These results benefit closely link their modern pharmacological activity with traditional medicinal efficacy for the treatment of chest stuffiness and pains. Actually, these secondary metabolites were biosynthesized by a series of enzymatic reactions of primary metabolites. The chemical profiles, visual spatial distribution, and absolute quantification of these secondary metabolites were presented in previous studies [6, 12, 13, 17, 20, 29]. However, except amino acids, limited researches about their primary metabolites were reported. These brought an obstacle to the interpretation of their nutritional and medicinal values.

Another novelty was that UHPLC/MS–MS, GC–MS and ICP-MS were used to analyze the visual spatial distribution and content levels of some primary metabolites in these two *Allium* species for the first time. To a certain extent, the category similarity of amino acids provides obvious evidences about both AMB and ACGD bulbs considered as the botanical origins of Xiebai (Fig. 8A). Besides, the fact that total content levels of all amino acids in ACGD were nearly 2.4-fold of those in AMB (Fig. 8F). This implied that ACGD may have higher medicinal and nutritional values than AMB. This kept in line with previous study [3]. Among them, some amino acids or their metabolites could benefit the cardiovascular protection. For example, the arginine in human can be metabolized to nitric oxide, which further prevents the platelet aggregation and relaxes blood vessels [30]. In addition, it has potential benefits for the patients with hypercholesterolemia or cardiovascular disease by daily supplementation of citrulline and arginine [31]. γ-aminobutyric acid is a neurotransmission inhibitor [32]. Due to its ability to lower blood pressure, it has been developed as a product for people with hypertension [32].

Except these amino acids, omega-3 polyunsaturated fatty acids (PUFA) in AMB and ACGD could also benefit the prevention of cardiovascular diseases [33]. This was because higher concentrations of omega-3 PUFA taken in human body were associated with a significantly reduced risk of total cardiovascular diseases [33]. Obviously, the total contents of omega-3 PUFA in AMB and ACGD were 46.67 and 22.96 μg/g, respectively (Additional file 1: Table S4). Further, proper consumption of carotenoid-rich products may help to inhibit the development of cardiovascular disease [34]. In this study, the absolute content levels of β-carotene exhibited no significant differences (both 0.08 μg/g) in these two herbs (Fig. 8C). So far, it is still inconclusive that proper dietary supplementation of vitamins can decrease the risks of cardiovascular diseases [35, 36]. Dietary interventions aiming at optimizing the intake of important trace elements can play an critical role in treating dyslipidemia [37]. These two *Allium* herbs were both rich in trace elements, especially Mg and Ca (Fig. 8E). The clinical efficacy of AMB and ACGD herbs as traditional herbal agent in improving cardiovascular diseases probably were attributed to a synergy of all the above-mentioned primary and secondary metabolites.

In this study, MALDI-TOF MSI approach allowed us to visualize the main primary metabolites in these two *Allium* fresh bulbs. The visual spatial information benefited the understanding about nutrients accumulation during their growth and developing process [38]. A total of 61 main primary metabolites were detected and exactly identified, along with their visual information on the fresh bulb sections (Table 1). Most metabolites beard similar spatial distribution patterns. They were extensively located in the outer leaf scale and tunics regions of AMB bulbs. These main components were mostly distributed in ACGD whole leaf scales and tunics (Figs. 4, 5, 6, 7). However, there were some exceptions. l-Alanine (Fig. 4A), and vitamin B1 (Fig. 7A) in AMB, l-histidine (Fig. 4D), l-serine (Fig. 4F), l-tryptophan (Additional file 1: Fig. S5A), *N*-acetylneuraminic acid (Additional file 1: Fig. S6F), vitamin B1 (Fig. 7A), vitamin B2 (Fig. 7B), were all abundant in the regions of developing flower buds in ACGD. Notably, the isomers like zeaxanthin and lutein (Fig. 6B), and violaxanthin and neoxanthin (Fig. 6D), both cannot be efficiently separated for respective visual MSI image. Each MALDI-TOF MSI image is an sum overlap of visual image with the same *m/z* ions [6]. This is due to the limitations of in situ detection. This brought a major limitation during the MALDI MSI application.

Although MALDI-TOF MSI analysis can map spatial distribution of interested compounds in section, inefficient chromatographic separation of complex mixture still limited its application [39]. The major obstacle was that the adduct ions with same *m/z* values was hard to distinguish, which further brought ambiguity about the characterization of spatial location of each component [39]. When the MSI relative quantification and absolute quantification results of some analytes were different, absolute quantification results were more reliable. In this study, it was the case for zeaxanthin and lutein (Fig. 6B), and violaxanthin and neoxanthin (Fig. 6D). For this limitation, several solutions were employed to distinguish the isomers in visual MSI images. For instance, [M+Li]^+^ ions were usually used as the parent ions for MS/MS fragmentation. This was because it was difficult to obtain the fragmentation information of [M+Na]^+^ and [M+K]^+^ ions. Several minor differences in MS/MS spectra at *m/z* 253.13 and *m/z* 255.11 were observed and considered as the diagnostic basis for albiflorin and paeoniflorin, respectively [40]. Another solution was that authentic standards with serial different concentration was evenly sprayed on the sections for further MALDI MSI imaging. This approach was successfully used to explore the spatial distribution and absolute quantification (ng/mm^2^) of two trimethylammonium derivatives [41]. In addition, combination of MALDI-TOF MSI imaging analysis and conventional quantitative approaches were usually used to elucidate the connotation of interested compounds [38]. In this study, UHPLC/MS–MS, GC–MS and ICP-MS approaches-based absolute content levels supported the relative intensity results derived from MALDI-TOF MSI method (Figs. 4, 5, 6, 7).

## Conclusion

In summary, AMB extracts demonstrated better protection against myocardial injury than ACGD extracts. In addition, a total of 61 main primary metabolites were performed for visual spatial distribution by MALDI-TOF MSI approach. They were likely distributed in whole bulbs, but mainly in the regions of outside leaf scales and tunics of AMB, and the whole leaf scales and tunics of ACGD. And individual component can be detected mainly in developing flower buds. Further, the total contents of some primary metabolites showed prominent differences between these two herbs. Amino acids were richer in ACGD, while fatty acids and vitamins were more abundant in AMB. To a certain extent, these quantitative results supported the absolute intensity/mass results by MALDI-TOF MSI analysis. Taken together, this study provided scientific several data for the similarity and differences of myocardial injury protection, spatial distribution and content levels of main primary metabolites between AMB and ACGD bulbs.

### Supplementary Information

**Additional file 1: Table S1.** The retention times, multiple reaction monitoring parameters, calibration curves, correlation factors, linear ranges, LLOQs, and ULOQs of amino acids and their derivates from the fresh bulbs of AMB and ACGD by UHPLC/QTRAP-MS in positive ion mode. **Table S2.** The qualitative and quantitative results of amino acid and their derivates in AMB and ACGD by UHPLC/QTRAP-MS. **Table S3.** The retention times, multiple reaction monitoring parameters, calibration curves, correlation factors, linear ranges, LLOQs, and ULOQs of free fatty acids (FFAs) from the fresh bulbs of AMB and ACGD by GC–MS. **Table S4.** The qualitative and quantitative results of free fatty acids (FFAs) in AMB and ACGD by GC–MS. **Table S5.** The retention times, multiple reaction monitoring parameters, calibration curves, correlation factors, linear ranges, LLOQs, and ULOQs of carotenoids from the fresh bulbs of AMB and ACGD by UHPLC/QTRAP-MS in positive ion mode. **Table S6.** The qualitative and quantitative results of carotenoids in AMB and ACGD by UHPLC/QTRAP-MS. **Table S7.** The retention times, multiple reaction monitoring parameters, calibration curves, correlation factors, linear ranges, LLOQs, and ULOQs of vitamins from the fresh bulbs of AMB and ACGD by UHPLC/QTRAP-MS in positive ion mode. **Table S8.** The qualitative and quantitative results of vitamins in AMB and ACGD by UHPLC/QTRAP-MS. **Table S9.** The retention times, multiple reaction monitoring parameters, calibration curves, correlation factors, linear ranges, LLOQs, and ULOQs of trace elements from the fresh bulbs of AMB and ACGD by UHPLC/QTRAP-MS in positive ion mode. **Table S10.** The qualitative and quantitative results of trace elements in AMB and ACGD by UHPLC/QTRAP-MS. **Fig S1.** Multiple reaction monitoring (MRM) chromatograms of free amino acids and their derivatives from fresh AMB and ACGD bulbs by UHPLC/MS–MS in positive (A) and negative (B) ion modes. **Fig S2.** Total ion chromatograms of free fatty acids and their derivatives from fresh AMB and ACGD bulbs by GC–MS. **Fig S3.** MRM chromatograms of carotenoids and their derivatives from fresh AMB and ACGD bulbs by UHPLC/MS–MS in positive ion mode. **Fig S4.** Multiple reaction monitoring (MRM) chromatograms of fat-soluble vitamins (A) and water-soluble vitamins (B) from fresh AMB and ACGD bulbs by UHPLC/MS–MS in positive and negative ion modes. **Fig S5.** MALDI-TOF MSI and quantitative LC–MS analysis of six amino acids in fresh AMB and ACGD bulbs. (A) L-Tryptophan at *m/z* 447.1435 ([2M+K]^+^); (B) L-Arginine at *m/z* 349.2312 ([2M+H]^+^); (C) L-Threonine at *m/z* 277.0802 ([2M+K]^+^); (D) L-Valine at *m/z* 273.1217 ([2M+K]^+^); (E) L-Ornithine at *m/z* 303.1435 ([2M+K]^+^); (F) L-Citrulline at *m/z* 373.1812 ([2M+Na]^+^); Each row presents the respective ion images, as well as relative quantification (intensity) extracted from the IMS measurements and absolute quantification (μg/g) determined by LC–MS approaches. The scale is 1.0 mm. (* compared with AMB group, * *p* < 0.05, ** *p* < 0.01, *** *p* < 0.001). **Fig S6.** MALDI-TOF IMS and quantitative LC–MS analysis of several amino acids in fresh AMB and ACGD bulbs. (A) argininosuccinic acid at *m/z* 291.1305 ([M+H]^+^); (B) Glutathione Oxidized at *m/z* 651.1157 ([M+K]^+^); (C) γ-Glutamate-Cysteine at *m/z* 273.0521 ([M+Na]^+^); (D) L-Tryptophyl-L-glutamic acid at *m/z* 372.0962 ([M+K]^+^); (E) N'-Formylkynurenine at *m/z* 259.0695 ([M+Na]^+^); (F) N-Acetylneuraminic Acid at *m/z* 332.0958 ([M+Na]^+^); Each row presents the respective ion images, as well as relative quantification (intensity) extracted from the IMS measurements and absolute quantification (μg/g) determined by LC–MS approaches. The scale is 1.0 mm. (* compared with AMB group, * *p* < 0.05, ** *p* < 0.01, *** *p* < 0.001). **Fig S7.** MALDI-TOF IMS and quantitative LC–MS analysis of several amino acids in fresh AMB and ACGD bulbs. (A) L-Asparagine Anhydrous at *m/z* 303.0707 ([2M+K]^+^); (B) L-Glutamine at *m/z* 331.1020 ([2M+K]^+^); (C) L-Glutamic acid at *m/z* 295.1141 ([2M+H]^+^); (D) Succinic Acid at *m/z* 237.0610 ([2M+H]^+^); (E) γ-Aminobutyric Acid at *m/z* 348.1537 ([3M+K]^+^); (F) (5-L-Glutamyl)-L-Amino Acid at *m/z* 475.1443 ([2M+K]^+^); Each row presents the respective ion images, as well as relative quantification (intensity) extracted from the IMS measurements and absolute quantification (μg/g) determined by LC–MS approaches. The scale is 1.0 mm. (* compared with AMB group, * *p* < 0.05, ** *p* < 0.01, *** *p* < 0.001). **Fig S8.** MALDI-TOF IMS and quantitative LC–MS analysis of several fatty acids in fresh AMB and ACGD bulbs. (A) palmitic acid at *m/z* 295.2039 ([M+K]^+^); (B) stearic acid at *m/z* 323.2352 ([M+K]^+^); (C) arachidic acid (AA) at *m/z* 351.2665 ([M+K]^+^); (D) behenic acid at *m/z* 379.2978 ([M+K]^+^); (E) lignoceric acid at *m/z* 407.3291 ([M+K]^+^); (F) cis-9-octadecenoic acid at *m/z* 283.2637 ([M+H]^+^); Each row presents the respective ion images, as well as relative quantification (intensity) extracted from the IMS measurements and absolute quantification (μg/g) determined by LC–MS approaches. The scale is 1.0 mm. (* compared with AMB group, * *p* < 0.05, ** *p* < 0.01, *** *p* < 0.001). **Fig S9.** MALDI-TOF IMS and quantitative LC–MS analysis of several carotenoids and their derivatives in fresh AMB and ACGD bulbs. (A) violaxanthin dilaurate at *m/z* 966.7676 ([M+H]^+^); (B) violaxanthin-myristate-laurate at *m/z* 1031.7470 ([M+K]^+^); (C) violaxanthin-myristate-caprate at *m/z* 965.7598 ([M+H]^+^); (D) zeaxanthin dimyristate at *m/z* 1011.8145 ([M+Na]^+^); Each row presents the respective ion images, as well as relative quantification (intensity) extracted from the IMS measurements and absolute quantification (μg/g) determined by LC–MS approaches. The scale is 1.0 mm. (* compared with AMB group, * *p* < 0.05, ** *p* < 0.01, *** *p* < 0.001). **Fig S10.** MALDI-TOF IMS and quantitative LC–MS analysis of several water-soluble vitamins in fresh AMB and ACGD bulbs. (A) Vitamin B5 (pantothenic acid) at *m/z* 477.1851 ([2M+K]^+^); (B) BDC (pyridoxine) at *m/z* 377.1115 ([2M+K]^+^); (C) Vitamin B7 (biotin) at *m/z* 283.0519 ([M+K]^+^); (D) Vitamin B9 at *m/z* 464.1295 ([M+Na]^+^); (E) 5-MTHFA (5-Methyltetrahydrofolate) at *m/z* 498.1503 ([M+K]^+^); Each row presents the respective ion images, as well as relative quantification (intensity) extracted from the IMS measurements and absolute quantification (μg/g) determined by LC–MS approaches. The scale is 1.0 mm. (* compared with AMB group, * *p* < 0.05, ** *p* < 0.01, *** *p* < 0.001). **Fig S11.** MALDI-TOF IMS and quantitative LC–MS analysis of several fat-soluble vitamins in fresh AMB and ACGD bulbs. (A) Vitamin A at *m/z* 325.1934 ([M+K]^+^); (B) Vitamin D2 at *m/z* 451.2978 ([M+K]^+^); (C) Vitamin D3 at *m/z* 439.2978 ([M+K]^+^); (D) Vitamin E at *m/z* 431.3889 ([M+H]^+^); Each row presents the respective ion images, as well as relative quantification (intensity) extracted from the IMS measurements and absolute quantification (μg/g) determined by LC–MS approaches. The scale is 1.0 mm. (* compared with AMB group, * *p* < 0.05, ** *p* < 0.01, *** *p* < 0.001). **Fig S12.** Comparison of content levels (μg/g) of twenty-one trace elements in fresh AMB and ACGD samples. (* compared with AMB group, * *p* < 0.05, ** *p* < 0.01, *** *p* < 0.001).

## Data Availability

The data in the present study are included in this article and its additional information.

## Abbreviations

AMB: *Allium macrostemon* Bunge
ACGD: *Allium chinense* G. Don
α-LA: α-Linolenic acid
CK-MB: Creatine kinase muscle brain fraction
CMC-Na: Sodium carboxymethyl cellulose
Dex: Dexrazoxane
DHA: Cis-4,7,10,13,16,19-docosahexaenoic acid
DPA: Cis-7,10,13,16,19-docosapentaenoic acid
EPA: Cis-5,8,11,14,17-eicosapentaenoic acid
LC–MS: Liquid chromatography tandem mass spectrometry
GC–MS: Gas chromatography tandem mass spectrometry
H/R: Hypoxia-reoxygenation
ITO: Indium tin oxide
LDH: Lactate dehydrogenase
MALDI-TOF MSI: Matrix-assisted laser desorption/ionization time-of-flight imaging mass spectrometry
MRM: Multiple reaction monitoring
MTT: Methyl-thiazolyldiphenyl-tetrazolium bromide
NO: Nitric oxide
SIM: Selective ion monitoring
PUFA: Polyunsaturated fatty acids
