# Supplementary material for: Deciphering the benefits and intensity levels of primary metabolites from Allium macrostemon Bunge and Allium chinense G. Don
Source: Chin Med. 2024 Jul 15;19:99. doi: 10.1186/s13020-024-00957-3 (PMC11251333; doi:10.1186/s13020-024-00957-3)
Supplement: Supplementary file 1 — Additional file 1: Table S1. The retention times, multiple reaction monitoring parameters, calibration curves, correlation factors, linear ranges, LLOQs, and ULOQs of amino acids and their derivates from the fresh bulbs of AMB and ACGD by UHPLC/QTRAP-MS in positive ion mode. Table S2. The qualitative and quantitative results of amino acid and their derivates in AMB and ACGD by UHPLC/QTRAP-MS. Table S3. The retention times, multiple reaction monitoring parameters, calibration curves, correlation factors, linear ranges, LLOQs, and ULOQs of free fatty acids (FFAs) from the fresh bulbs of AMB and ACGD by GC–MS. Table S4. The qualitative and quantitative results of free fatty acids (FFAs) in AMB and ACGD by GC–MS. Table S5. The retention times, multiple reaction monitoring parameters, calibration curves, correlation factors, linear ranges, LLOQs, and ULOQs of carotenoids from the fresh bulbs of AMB and ACGD by UHPLC/QTRAP-MS in positive ion mode. Table S6. The qualitative and quantitative results of carotenoids in AMB and ACGD by UHPLC/QTRAP-MS. Table S7. The retention times, multiple reaction monitoring parameters, calibration curves, correlation factors, linear ranges, LLOQs, and ULOQs of vitamins from the fresh bulbs of AMB and ACGD by UHPLC/QTRAP-MS in positive ion mode. Table S8. The qualitative and quantitative results of vitamins in AMB and ACGD by UHPLC/QTRAP-MS. Table S9. The retention times, multiple reaction monitoring parameters, calibration curves, correlation factors, linear ranges, LLOQs, and ULOQs of trace elements from the fresh bulbs of AMB and ACGD by UHPLC/QTRAP-MS in positive ion mode. Table S10. The qualitative and quantitative results of trace elements in AMB and ACGD by UHPLC/QTRAP-MS. Fig S1. Multiple reaction monitoring (MRM) chromatograms of free amino acids and their derivatives from fresh AMB and ACGD bulbs by UHPLC/MS–MS in positive (A) and negative (B) ion modes. Fig S2. Total ion chromatograms of free fatty acids and their derivatives from [file 13020_2024_957_MOESM1_ESM.docx]

**Supporting information**

Deciphering the benefits and intensity levels of primary metabolites from *Allium macrostemon* Bunge and *Allium chinense* G. Don

Zifei Qin^1,#^, Yuan Li^1,#^, Dongmei Liu^1^, Yuzhuo Hua^2^, Yuandong Lv^3^, Xiaojian Zhang^1,4^, Cailian Fan^2^, Jing Yang^1,4^*

^1^ Department of Pharmacy, the First Affiliated Hospital of Zhengzhou University, Zhengzhou 450052, China;

^2^ College of Medicine, Henan Engineering Research Center of Funiu Mountain’s Medicinal Resources Utilization and Molecular Medicine, Pingdingshan University, Pingdingshan 467000, China;

^3^ Hangzhou EXPECLIN Medical Technology Co., Ltd., Hangzhou 311305, China;

^4^ Henan Engineering Research Center of Application & Translation of Precision Clinical Pharmacy, Zhengzhou 450052, China;

^#^ These authors contributed equally to this work.

***Corresponding Authors:**

Associate Prof Jing Yang, jingyang_0101@163.com;

**Table lists**

**Table S1** The retention times, multiple reaction monitoring parameters, calibration curves, correlation factors, linear ranges, LLOQs, and ULOQs of amino acids and their derivates from the fresh bulbs of AMB and ACGD by UHPLC/QTRAP-MS in positive ion mode.

**Table S2** The qualitative and quantitative results of amino acid and their derivates in AMB and ACGD by UHPLC/QTRAP-MS.

**Table S3** The retention times, multiple reaction monitoring parameters, calibration curves, correlation factors, linear ranges, LLOQs, and ULOQs of free fatty acids (FFAs) from the fresh bulbs of AMB and ACGD by GC-MS.

**Table S4** The qualitative and quantitative results of free fatty acids (FFAs) in AMB and ACGD by GC-MS.

**Table S5** The retention times, multiple reaction monitoring parameters, calibration curves, correlation factors, linear ranges, LLOQs, and ULOQs of carotenoids from the fresh bulbs of AMB and ACGD by UHPLC/QTRAP-MS in positive ion mode.

**Table S6** The qualitative and quantitative results of carotenoids in AMB and ACGD by UHPLC/QTRAP-MS.

**Table S7** The retention times, multiple reaction monitoring parameters, calibration curves, correlation factors, linear ranges, LLOQs, and ULOQs of vitamins from the fresh bulbs of AMB and ACGD by UHPLC/QTRAP-MS in positive ion mode.

**Table S8** The qualitative and quantitative results of vitamins in AMB and ACGD by UHPLC/QTRAP-MS.

**Table S9** The retention times, multiple reaction monitoring parameters, calibration curves, correlation factors, linear ranges, LLOQs, and ULOQs of trace elements from the fresh bulbs of AMB and ACGD by UHPLC/QTRAP-MS in positive ion mode.

**Table S10** The qualitative and quantitative results of trace elements in AMB and ACGD by UHPLC/QTRAP-MS.

**Figure captions**

**Figure S1** Multiple reaction monitoring (MRM) chromatograms of free amino acids and their derivatives from fresh AMB and ACGD bulbs by UHPLC/MS-MS in positive (A) and negative (B) ion modes.

**Figure S2** Total ion chromatograms (TIC) of free fatty acids and their derivatives from fresh AMB and ACGD bulbs by GC-MS.

**Figure S3** MRM chromatograms of carotenoids and their derivatives from fresh AMB and ACGD bulbs by UHPLC/MS-MS in positive ion mode.

**Figure S4** Multiple reaction monitoring (MRM) chromatograms of fat-soluble vitamins (A) and water-soluble vitamins (B) from fresh AMB and ACGD bulbs by UHPLC/MS-MS in positive and negative ion modes.

**Figure S5** MALDI-TOF IMS and quantitative LC-MS analysis of six amino acids in fresh AMB and ACGD bulbs. (A) L-Tryptophan at *m/z* 447.1435 ([2M+K]^+^); (B) L-Arginine at *m/z* 349.2312 ([2M+H]^+^); (C) L-Threonine at *m/z* 277.0802 ([2M+K]^+^); (D) L-Valine at *m/z* 273.1217 ([2M+K]^+^); (E) L-Ornithine at *m/z* 303.1435 ([2M+K]^+^); (F) L-Citrulline at *m/z* 373.1812 ([2M+Na]^+^); Each row presents the respective ion images, as well as relative quantification (intensity) extracted from the IMS measurements and absolute quantification (μg/g) determined by LC-MS approaches. The scale is 1.0 mm. (* compared with AMB group, * *p* < 0.05, ** *p* < 0.01, *** *p* < 0.001)

**Figure S6** MALDI-TOF IMS and quantitative LC-MS analysis of several amino acids in fresh AMB and ACGD bulbs. (A) argininosuccinic acid at *m/z* 291.1305 ([M+H]^+^); (B) Glutathione Oxidized at *m/z* 651.1157 ([M+K]^+^); (C) γ-Glutamate-Cysteine at *m/z* 273.0521 ([M+Na]^+^); (D) L-Tryptophyl-L-glutamic acid at *m/z* 372.0962 ([M+K]^+^); (E) N'-Formylkynurenine at *m/z* 259.0695 ([M+Na]^+^); (F) N-Acetylneuraminic Acid at *m/z* 332.0958 ([M+Na]^+^); Each row presents the respective ion images, as well as relative quantification (intensity) extracted from the IMS measurements and absolute quantification (μg/g) determined by LC-MS approaches. The scale is 1.0 mm. (* compared with AMB group, * *p* < 0.05, ** *p* < 0.01, *** *p* < 0.001)

**Figure S7** MALDI-TOF IMS and quantitative LC-MS analysis of several amino acids in fresh AMB and ACGD bulbs. (A) L-Asparagine Anhydrous at *m/z* 303.0707 ([2M+K]^+^); (B) L-Glutamine at *m/z* 331.1020 ([2M+K]^+^); (C) L-Glutamic acid at *m/z* 295.1141 ([2M+H]^+^); (D) Succinic Acid at *m/z* 237.0610 ([2M+H]^+^); (E) γ-Aminobutyric Acid at *m/z* 348.1537 ([3M+K]^+^); (F) (5-L-Glutamyl)-L-Amino Acid at *m/z* 475.1443 ([2M+K]^+^); Each row presents the respective ion images, as well as relative quantification (intensity) extracted from the IMS measurements and absolute quantification (μg/g) determined by LC-MS approaches. The scale is 1.0 mm. (* compared with AMB group, * *p* < 0.05, ** *p* < 0.01, *** *p* < 0.001)

**Figure S8** MALDI-TOF IMS and quantitative LC-MS analysis of several fatty acids in fresh AMB and ACGD bulbs. (A) palmitic acid at *m/z* 295.2039 ([M+K]^+^); (B) stearic acid at *m/z* 323.2352 ([M+K]^+^); (C) arachidic acid (AA) at *m/z* 351.2665 ([M+K]^+^); (D) behenic acid at *m/z* 379.2978 ([M+K]^+^); (E) lignoceric acid at *m/z* 407.3291 ([M+K]^+^); (F) cis-9-octadecenoic acid at *m/z* 283.2637 ([M+H]^+^); Each row presents the respective ion images, as well as relative quantification (intensity) extracted from the IMS measurements and absolute quantification (μg/g) determined by LC-MS approaches. The scale is 1.0 mm. (* compared with AMB group, * *p* < 0.05, ** *p* < 0.01, *** *p* < 0.001)

**Figure S9** MALDI-TOF IMS and quantitative LC-MS analysis of several carotenoids and their derivatives in fresh AMB and ACGD bulbs. (A) violaxanthin dilaurate at *m/z* 966.7676 ([M+H]^+^); (B) violaxanthin-myristate-laurate at *m/z* 1031.7470 ([M+K]^+^); (C) violaxanthin-myristate-caprate at *m/z* 965.7598 ([M+H]^+^); (D) zeaxanthin dimyristate at *m/z* 1011.8145 ([M+Na]^+^); Each row presents the respective ion images, as well as relative quantification (intensity) extracted from the IMS measurements and absolute quantification (μg/g) determined by LC-MS approaches. The scale is 1.0 mm. (* compared with AMB group, * *p* < 0.05, ** *p* < 0.01, *** *p* < 0.001)

**Figure S10** MALDI-TOF IMS and quantitative LC-MS analysis of several water-soluble vitamins in fresh AMB and ACGD bulbs. (A) Vitamin B5 (pantothenic acid) at *m/z* 477.1851 ([2M+K]^+^); (B) BDC (pyridoxine) at *m/z* 377.1115 ([2M+K]^+^); (C) Vitamin B7 (biotin) at *m/z* 283.0519 ([M+K]^+^); (D) Vitamin B9 at *m/z* 464.1295 ([M+Na]^+^); (E) 5-MTHFA (5-Methyltetrahydrofolate) at *m/z* 498.1503 ([M+K]^+^); Each row presents the respective ion images, as well as relative quantification (intensity) extracted from the IMS measurements and absolute quantification (μg/g) determined by LC-MS approaches. The scale is 1.0 mm. (* compared with AMB group, * *p* < 0.05, ** *p* < 0.01, *** *p* < 0.001)

**Figure S11** MALDI-TOF IMS and quantitative LC-MS analysis of several fat-soluble vitamins in fresh AMB and ACGD bulbs. (A) Vitamin A at *m/z* 325.1934 ([M+K]^+^); (B) Vitamin D2 at *m/z* 451.2978 ([M+K]^+^); (C) Vitamin D3 at *m/z* 439.2978 ([M+K]^+^); (D) Vitamin E at *m/z* 431.3889 ([M+H]^+^); Each row presents the respective ion images, as well as relative quantification (intensity) extracted from the IMS measurements and absolute quantification (μg/g) determined by LC-MS approaches. The scale is 1.0 mm. (* compared with AMB group, * *p* < 0.05, ** *p* < 0.01, *** *p* < 0.001)

**Figure S12** Comparison of content levels (μg/g) of twenty-one trace elements in fresh AMB and ACGD samples. (* compared with AMB group, * *p* < 0.05, ** *p* < 0.01, *** *p* < 0.001)

**Table S1** The retention times, multiple reaction monitoring parameters, calibration curves, correlation factors, linear ranges, LLOQs, and ULOQs of amino acids and their derivates from the fresh bulbs of AMB and ACGD by UHPLC/QTRAP-MS in positive ion mode.

| **No.** | **Compounds** | **Class** | **Formula** | **RT**  **(min)** | **Ion** | **Q1**  **(Da)** | **Q3**  **(Da)** | **Calibration curves** | ***R*^2^** | **Linear range**  **(μg/mL)** | **LLOQ**  **(μg/mL)** | **ULOQ**  **(μg/mL)** |
| --- | --- | --- | --- | --- | --- | --- | --- | --- | --- | --- | --- | --- |
| A1 | L-Alanine | Amino Acid | C3H7NO2 | 8.29 | [M+H]+ | 90.1 | 44.1 | Y = 7.0097e-4 X + 0.0376 | 0.9990 | 0.1-20 | 0.1 | 20 |
| A2 | L-Arginine | Amino Acid | C6H14N4O2 | 10.27 | [M+H]+ | 175.1 | 70.1 | Y = 0.0062 X - 0.3431 | 0.9968 | 0.01-20 | 0.01 | 20 |
| A3 | L-Asparagine Anhydrous | Amino Acid | C4H8N2O3 | 9.19 | [M+H]+ | 133.1 | 74.0 | Y = 6.9335e-4 X - 0.0247 | 0.9999 | 0.05-20 | 0.05 | 20 |
| A4 | L-Aspartate | Amino Acid | C4H7NO4 | 9.97 | [M+H]+ | 134.0 | 74.0 | Y = 3.4191e-4 X + 0.0101 | 0.9990 | 0.1-20 | 0.1 | 20 |
| A5 | L-Cysteine | Amino Acid | C3H7NO2S | 7.62 | [M+H]+ | 122.0 | 59.0 | Y = 2.0659e-4 X - 0.0326 | 0.9909 | 1-20 | 1 | 20 |
| A6 | L-Glutamine | Amino Acid | C5H10N2O3 | 9.05 | [M+H]+ | 147.1 | 84.0 | Y = 5.1564e-4 X - 0.0226 | 0.9994 | 0.05-20 | 0.05 | 20 |
| A7 | L-Glutamic acid | Amino Acid | C5H9NO4 | 9.48 | [M+H]+ | 148.1 | 84.0 | Y = 3.8522e-4 X + 0.0161 | 0.9946 | 0.01-20 | 0.01 | 20 |
| A8 | Glycine | Amino Acid | C2H5NO2 | 8.73 | [M+H]+ | 76.0 | 30.0 | Y = 4.2154e-5 X + 0.0023 | 0.9983 | 0.2-20 | 0.2 | 20 |
| A9 | L-Histidine | Amino Acid | C6H9N3O2 | 10.05 | [M+H]+ | 156.1 | 110.1 | Y = 0.0014 X - 0.0959 | 0.9979 | 0.5-20 | 0.5 | 20 |
| A10 | L-Isoleucine | Amino Acid | C6H13NO2 | 5.59 | [M+H]+ | 132.1 | 86.1 | Y = 0.0083 X - 0.1802 | 0.9988 | 0.05-20 | 0.05 | 20 |
| A11 | L-Cystine | Amino Acid | C6H12N2O4S2 | 10.78 | [M-H]- | 239.0 | 120.0 | Y = 2.7543e-5 X + 0.0008 | 0.9997 | 0.01-10 | 0.01 | 10 |
| A12 | L-Leucine | Amino Acid | C6H13NO2 | 5.26 | [M+H]+ | 132.1 | 86.1 | Y = 0.0081 X - 0.0240 | 0.9996 | 0.1-20 | 0.1 | 20 |
| A13 | L-Lysine | Amino Acid | C6H14N2O2 | 10.40 | [M+H]+ | 147.1 | 84.0 | Y = 0.0041 X - 0.0505 | 0.9986 | 0.02-20 | 0.02 | 20 |
| A14 | L-Methionine | Amino Acid | C5H11NO2S | 6.36 | [M+H]+ | 150.1 | 104.0 | Y = 0.0021 X + 0.1077 | 0.9972 | 1-20 | 1 | 20 |
| A15 | L-Phenylalanine | Amino Acid | C9H11NO2 | 5.14 | [M+H]+ | 166.1 | 120.1 | Y = 0.0095 X - 0.3837 | 0.9990 | 0.01-20 | 0.01 | 20 |
| A16 | L-Proline | Amino Acid | C5H9NO2 | 7.02 | [M+H]+ | 116.1 | 70.1 | Y = 0.0032 X - 0.0994 | 0.9996 | 0.02-20 | 0.02 | 20 |
| A17 | L-Serine | Amino Acid | C3H7NO3 | 9.10 | [M+H]+ | 106.0 | 60.0 | Y = 7.0144e-5 X - 0.0014 | 0.9989 | 0.2-20 | 0.2 | 20 |
| A18 | L-Threonine | Amino Acid | C4H9NO3 | 8.54 | [M+H]+ | 120.1 | 74.0 | Y = 8.0734e-4 X - 0.0204 | 0.9988 | 0.1-20 | 0.1 | 20 |
| A19 | L-Tryptophan | Amino Acid | C11H12N2O2 | 5.28 | [M+H]+ | 205.1 | 118.1 | Y = 2.7179e-5 X + 0.00002 | 0.9976 | 0.05-20 | 0.05 | 20 |
| A20 | L-Tyrosine | Amino Acid | C9H11NO3 | 7.27 | [M+H]+ | 182.1 | 136.1 | Y = 0.0049 X - 0.1660 | 0.9994 | 0.05-20 | 0.05 | 20 |
| A21 | L-Valine | Amino Acid | C5H11NO2 | 7.00 | [M+H]+ | 118.1 | 72.1 | Y = 0.0074 X - 0.1936 | 0.9991 | 0.1-20 | 0.1 | 20 |
| A22 | Sarcosine | Amino Acid | C3H7NO2 | 7.87 | [M+H]+ | 90.2 | 44.1 | Y = 0.0014 X - 0.0396 | 0.9974 | 0.1-20 | 0.1 | 20 |
| A23 | L-Ornithine | Amino Acid | C5H13ClN2O2 | 10.44 | [M+H]+ | 133.1 | 70.0 | Y = 5.6552e-4 X + 0.0182 | 0.9991 | 0.05-20 | 0.05 | 20 |
| A24 | L-Citrulline | Amino Acid | C6H13N3O3 | 9.40 | [M+H]+ | 176.1 | 113.0 | Y = 3.2647e-4 X - 0.0128 | 0.9921 | 0.02-20 | 0.02 | 20 |
| A25 | L-Homocitrulline | Amino Acid | C7H15N3O3 | 9.16 | [M+H]+ | 190.1 | 127.0 | Y = 0.0034 X - 0.1676 | 0.9948 | 0.01-20 | 0.01 | 20 |
| A26 | Homo-L-arginine | Amino Acid | C7H16N4O2 | 10.12 | [M+H]+ | 189.0 | 144.0 | Y = 0.0083 X - 0.3893 | 0.9999 | 0.01-10 | 0.01 | 10 |
| A27 | S-(5-Adenosyl)-L-Homocysteine | Amino Acid | C14H20N6O5S | 9.36 | [M+H]+ | 385.0 | 136.1 | Y = 0.0013 X - 0.0387 | 0.9964 | 0.01-20 | 0.01 | 20 |
| A28 | N-α-Acetyl-L-glutamine | Amino Acid | C7H12N2O4 | 7.46 | [M+H]+ | 189.1 | 130.1 | Y = 2.4712e-4 X - 0.0043 | 0.9990 | 0.02-5 | 0.02 | 5 |
| A29 | Nicotinuric Acid | Amino Acid | C8H8N2O3 | 4.72 | [M+H]+ | 181.1 | 135.0 | Y = 0.0077 X - 0.2464 | 0.9989 | 0.01-20 | 0.01 | 20 |
| A30 | L-tyrosine methyl ester | Amino Acid | C10H13NO3 | 1.63 | [M+H]+ | 196.2 | 136.3 | Y = 0.0223 X | 0.9958 | 0.01-20 | 0.01 | 20 |
| A31 | Glycyl-L-Proline | Amino Acid | C7H12N2O3 | 9.15 | [M+H]+ | 173.1 | 116.1 | Y = 0.0149 X - 0.3603 | 0.9985 | 0.01-20 | 0.01 | 20 |
| A32 | Glutathione Oxidized | Amino Acid | C20H32N6O12S2 | 11.09 | [M+H]+ | 613.2 | 484.0 | Y = 3.1084e-6 X + 0.0244 | 0.9992 | 0.02-20 | 0.02 | 20 |
| A33 | argininosuccinic acid | Amino Acid | C10H18N4O6 | 10.84 | [M+H]+ | 291.5 | 176.1 | Y = 2.3384e-5 X - 0.0130 | 0.9992 | 0.02-20 | 0.02 | 20 |
| A34 | D-Alanyl-D-Alanine | Amino Acid | C6H12N2O3 | 8.89 | [M+H]+ | 161.1 | 44.0 | Y = 9.4458e-4 X - 0.0137 | 0.9998 | 0.01-20 | 0.01 | 20 |
| A35 | Trimethylamine N-Oxide | Amino Acid | C3H9NO | 3.43 | [M+H]+ | 76.0 | 58.1 | Y = 0.1688 X | 0.9991 | 0.01-20 | 0.01 | 20 |
| A36 | Trans-4-Hydroxy-L-Proline | Amino Acid | C5H9NO3 | 8.28 | [M+H]+ | 132.1 | 86.0 | Y = 0.0069 X - 0.2735 | 0.9987 | 0.1-20 | 0.1 | 20 |
| A37 | Succinic Acid | Amino Acid | C4H6O4 | 1.71 | [M-H]- | 117.0 | 99.0 | Y = 3.3862e-6 X + 0.0052 | 0.9994 | 0.1-20 | 0.1 | 20 |
| A38 | glycylphenylalanine | Amino Acid | C11H14N2O3 | 7.55 | [M+H]+ | 223.2 | 120.2 | Y = 0.0037 X - 0.1020 | 0.9980 | 0.01-20 | 0.01 | 20 |
| A39 | γ-Glutamate-Cysteine | Amino Acid | C8H14N2O5S | 9.86 | [M+H]+ | 251.1 | 130.1 | Y = 5.0528e-5 X - 0.0020 | 0.9973 | 5-20 | 5 | 20 |
| A40 | S-Sulfo-L-Cysteine | Amino Acid | C3H7NO5S2 | 8.13 | [M+H]+ | 202.0 | 120.0 | Y = 1.6099e-4 X + 0.0033 | 0.9976 | 0.01-20 | 0.01 | 20 |
| A41 | 5-Hydroxy-Tryptamine | Amino Acid | C10H12N20 | 3.04 | [M+H]+ | 177.1 | 160.0 | Y = 5.2898e-5 X + 0.0496 | 0.9969 | 0.05-20 | 0.05 | 20 |
| A42 | Urea | Amino Acid | CH4N2O | 1.62 | [M+H]+ | 61.0 | 44.0 | Y = 6.1776e-4 X + 0.0436 | 0.9993 | 0.5-20 | 0.5 | 20 |
| A43 | N-Propionylglycine | Amino Acid | C5H9NO3 | 2.88 | [M-H]- | 130.1 | 74.0 | Y = 6.7267e-4 X - 0.0161 | 0.9967 | 0.01-20 | 0.01 | 20 |
| A44 | L-Carnosine | Amino Acid | C9H14N4O3 | 10.44 | [M+H]+ | 227.2 | 156.0 | Y = 9.6672e-4 X - 0.0418 | 0.9986 | 0.05-20 | 0.05 | 20 |
| A45 | Homoserine | Amino Acid | C4H9NO3 | 8.69 | [M+H]+ | 120.1 | 74.1 | Y = 7.0250e-4 X - 0.0193 | 0.9991 | 0.05-20 | 0.05 | 20 |
| A46 | (5-L-Glutamyl)-L-Amino Acid | Amino Acid | C8H14N2O5 | 9.89 | [M+H]+ | 219.1 | 202.0 | Y = 2.6062e-4 X + 0.0021 | 0.9975 | 0.1-20 | 0.1 | 20 |
| A47 | N6-Acetyl-L-Lysine | Amino Acid | C8H16N2O3 | 8.42 | [M+H]+ | 189.1 | 126.0 | Y = 0.0146 X - 0.0597 | 0.9996 | 0.01-20 | 0.01 | 20 |
| A48 | Nα-Acetyl-L-Arginine | Amino Acid | C8H16N4O3 | 8.54 | [M+H]+ | 217.1 | 158.0 | Y = 0.0053 X - 0.1490 | 0.9988 | 0.01-20 | 0.01 | 20 |
| A49 | D-Homocysteine | Amino Acid | C4H9NO2S | 7.00 | [M+H]+ | 136.0 | 90.0 | Y = 5.9873e-4 X - 0.0145 | 0.9996 | 0.5-20 | 0.5 | 20 |
| A50 | Beta-Alanine | Amino Acid | C3H7NO2 | 8.33 | [M+H]+ | 90.2 | 30.0 | Y = 4.7977e-5 X - 0.0002 | 0.9986 | 0.1-20 | 0.1 | 20 |
| A51 | L-α-Aspartyl-L-phenylalanine | Amino Acid | C13H16N2O5 | 8.79 | [M+H]+ | 281.3 | 166.0 | Y = 0.0012 X + 0.0261 | 0.9993 | 0.01-20 | 0.01 | 20 |
| A52 | 5-Hydroxylysine | Amino Acid | C6H14N2O3 | 10.64 | [M+H]+ | 163.1 | 128.1 | Y = 5.3117e-4 X - 0.0156 | 0.9981 | 0.02-20 | 0.02 | 20 |
| A53 | 3-N-Methyl-L-Histidine | Amino Acid | C7H11N3O2 | 10.17 | [M+H]+ | 170.0 | 96.0 | Y = 0.0031 X - 0.1413 | 0.9990 | 0.05-20 | 0.05 | 20 |
| A54 | 3-Aminoisobutanoic Acid | Amino Acid | C4H9NO2 | 7.55 | [M+H]+ | 104.1 | 86.1 | Y = 1.6346e-4 X + 0.0038 | 0.9949 | 0.2-10 | 0.2 | 10 |
| A55 | 1-Methylhistidine | Amino Acid | C7H11N3O2 | 9.37 | [M+H]+ | 170.1 | 124.0 | Y = 0.0209 X - 1.6653 | 0.9926 | 0.05-20 | 0.05 | 20 |
| A56 | N-Glycyl-L-Leucine | Amino Acid | C8H16N2O3 | 7.59 | [M+H]+ | 189.1 | 86.0 | Y = 8.7688e-4 X - 0.0220 | 0.9979 | 0.01-10 | 0.01 | 10 |
| A57 | N-Acetylneuraminic Acid | Amino Acid | C11H19NO9 | 9.61 | [M+H]+ | 310.1 | 274.1 | Y = 1.1942e-4 X - 0.0052 | 0.9952 | 0.2-20 | 0.2 | 20 |
| A58 | N-Acetyl-L-Tyrosine | Amino Acid | C11H13NO4 | 2.29 | [M+H]+ | 224.0 | 136.0 | Y = 0.0030 X - 0.0664 | 0.9953 | 0.01-20 | 0.01 | 20 |
| A59 | Methionine Sulfoxide | Amino Acid | C5H11NO3S | 9.00 | [M+H]+ | 166.0 | 74.0 | Y = 0.0036 X - 0.1609 | 0.9961 | 0.02-20 | 0.02 | 20 |
| A60 | L-Theanine | Amino Acid | C7H14N2O3 | 7.57 | [M+H]+ | 175.0 | 158.0 | Y = 0.0074 X - 0.2933 | 0.9983 | 0.01-20 | 0.01 | 20 |
| A61 | L-Pipecolic Acid | Amino Acid | C6H11NO2 | 7.20 | [M+H]+ | 130.0 | 84.0 | Y = 0.0397 X - 0.9850 | 0.9970 | 0.01-5 | 0.01 | 5 |
| A62 | L-Tryptophyl-L-glutamic acid | Amino Acid | C16H19N3O5 | 8.39 | [M+H]+ | 334.0 | 159.0 | Y = 1.7462e-5 X + 0.00008 | 0.9967 | 0.01-20 | 0.01 | 20 |
| A63 | Creatine | Organic Acid | C4H9N3O2 | 8.28 | [M+H]+ | 132.1 | 90.0 | Y = 0.0090 X - 0.2786 | 0.9982 | 0.01-20 | 0.01 | 20 |
| A64 | (S)-β-Aminoisobutyric Acid | Organic Acid | C4H9NO2 | 7.56 | [M+H]+ | 104.1 | 57.0 | Y = 1.5854e-4 X - 0.0059 | 0.9997 | 0.02-20 | 0.02 | 20 |
| A65 | Kinurenine | Organic Acid | C10H12N2O3 | 5.31 | [M+H]+ | 209.1 | 146.0 | Y = 0.0052 X - 0.2069 | 0.9992 | 0.01-20 | 0.01 | 20 |
| A66 | 2-Aminobutyric acid | Organic Acid | C4H9NO2 | 7.74 | [M+H]+ | 104.1 | 58.1 | Y = 0.0036 X - 0.0772 | 0.9983 | 0.1-20 | 0.1 | 20 |
| A67 | 2-Aminoethanesulfonic Acid | Organic Acid | C2H7NO3S | 7.32 | [M-H]- | 124.0 | 80.0 | Y = 0.0054 X - 0.1365 | 0.9978 | 0.01-20 | 0.01 | 20 |
| A68 | 4-Acetamidobutyric Acid | Organic Acid | C6H11NO3 | 1.34 | [M+H]+ | 146.1 | 86.0 | Y = 0.0041 X - 0.0151 | 0.9980 | 0.01-20 | 0.01 | 20 |
| A69 | 5-Aminovaleric Acid | Organic Acid | C5H11NO2 | 5.79 | [M+H]+ | 118.2 | 55.0 | Y = 7.1342e-4 X - 0.0239 | 0.9992 | 0.01-20 | 0.01 | 20 |
| A70 | γ-Aminobutyric Acid | Organic Acid | C4H9NO2 | 7.45 | [M+H]+ | 104.1 | 68.8 | Y = 4.1138e-5 X + 0.0038 | 0.9977 | 0.1-10 | 0.1 | 10 |
| A71 | Kynurenic Acid | Organic Acid | C10H7NO3 | 3.70 | [M+H]+ | 190.0 | 144.0 | Y = 0.0513 X | 0.9967 | 0.01-5 | 0.01 | 5 |
| A72 | 6-Aminocaproic Acid | Organic Acid | C6H13NO2 | 4.77 | [M+H]+ | 132.1 | 69.0 | Y = 0.0010 X - 0.0283 | 0.9980 | 0.01-20 | 0.01 | 20 |
| A73 | 1,3,7-Trimethyluric Acid | Organic Acid | C8H10N4O3 | 1.20 | [M+H]+ | 211.1 | 154.1 | Y = 0.0033 X | 0.9991 | 0.01-10 | 0.01 | 10 |
| A74 | 3,7-Dimethyluric Acid | Organic Acid | C7H8N4O3 | 1.94 | [M-H]- | 195.0 | 180.0 | Y = 0.0018 X | 0.9981 | 0.01-20 | 0.01 | 20 |
| A75 | α-Aminoadipic acid | Organic Acid | C6H11NO4 | 9.08 | [M+H]+ | 162.1 | 98.1 | Y = 2.1446e-4 X - 0.0131 | 0.9982 | 0.01-20 | 0.01 | 20 |
| A76 | N'-Formylkynurenine | Organic Acid | C11H12N2O4 | 7.27 | [M+H]+ | 237.1 | 146.1 | Y = 3.5651e-4 X + 0.0021 | 0.9978 | 0.1-20 | 0.1 | 20 |

**Table S2** The qualitative and quantitative results of amino acids and their derivates in AMB and ACGD by UHPLC/QTRAP-MS.

| **No.** | **Compounds** | **Content levels (μg/g)** | | | | | | | | | | | |
| --- | --- | --- | --- | --- | --- | --- | --- | --- | --- | --- | --- | --- | --- |
|  |  | **AMB_1** | **AMB_2** | **AMB_3** | **AMB_4** | **AMB_5** | **AMB_6** | **ACGD_1** | **ACGD_2** | **ACGD_3** | **ACGD_4** | **ACGD_5** | **ACGD_6** |
| A1 | L-Alanine | 131.0625 | 192.4565 | 166.9104 | 177.4429 | 172.6716 | 149.7301 | 500.3881 | 317.4720 | 576.4940 | 419.2905 | 401.5104 | 429.5480 |
| A2 | L-Arginine | 1151.2570 | 1567.8246 | 1519.7795 | 1535.8093 | 1502.8882 | 1675.5298 | 1707.4815 | 1626.7300 | 1750.5289 | 1468.5712 | 1573.7859 | 1574.0664 |
| A3 | L-Asparagine Anhydrous | 988.6393 | 1732.8682 | 1686.4892 | 1661.2314 | 1586.0930 | 1481.2875 | 1602.8306 | 882.1770 | 2070.6687 | 1252.2992 | 1440.3172 | 1536.4190 |
| A4 | L-Aspartate | 606.2310 | 1004.5131 | 1120.1470 | 1156.5442 | 1004.3620 | 1043.1727 | 1096.6893 | 808.7140 | 1191.7166 | 812.0939 | 884.1615 | 1009.3160 |
| A5 | L-Cysteine | N/A | N/A | N/A | N/A | N/A | N/A | 2.2203 | 1.9447 | N/A | 2.2272 | 1.9673 | 2.0466 |
| A6 | L-Glutamine | 4401.5563 | 3801.3163 | 4155.8315 | 2826.1668 | 4081.0781 | 3200.7397 | 10705.9412 | 8469.7000 | 10085.4291 | 8749.7434 | 8726.4817 | 8685.5936 |
| A7 | L-Glutamic acid | 3706.5044 | 5078.8302 | 5206.7554 | 5035.4320 | 5046.7260 | 4730.4278 | 8560.9522 | 6278.6800 | 9709.7106 | 6547.5923 | 6452.8940 | 6919.9587 |
| A8 | Glycine | 16.8501 | 19.4320 | 17.4179 | 17.8922 | 20.0430 | 15.9723 | 54.2033 | 41.4549 | 63.6931 | 50.2104 | 48.6847 | 51.6707 |
| A9 | L-Histidine | 201.1512 | 261.2451 | 264.5589 | 240.3803 | 209.6014 | 217.5190 | 958.4227 | 561.1340 | 970.8413 | 694.6477 | 682.0575 | 722.9697 |
| A10 | L-Isoleucine | 16.7978 | 17.2082 | 16.7035 | 15.0740 | 17.8925 | 18.5409 | 64.6294 | 38.2773 | 78.8986 | 60.0674 | 58.5226 | 57.2641 |
| A11 | L-Cystine | 2.9622 | 10.8339 | 12.1707 | 14.7063 | 12.2256 | 11.7225 | 0.7219 | 0.4890 | 1.2806 | 0.5186 | 0.2821 | 0.6658 |
| A12 | L-Leucine | 19.4747 | 19.0257 | 16.5567 | 17.4008 | 22.5621 | 19.3541 | 37.0854 | 36.9528 | 52.7184 | 38.3448 | 40.4188 | 43.2716 |
| A13 | L-Lysine | 220.3980 | 337.0467 | 307.8065 | 291.8441 | 262.5935 | 251.0336 | 1087.3475 | 776.9810 | 1080.2395 | 821.6242 | 838.1546 | 857.6219 |
| A14 | L-Methionine | 6.3677 | 11.6440 | 9.0978 | 7.2694 | 9.6285 | 7.6988 | 44.3864 | 20.6712 | 53.7672 | 41.6253 | 30.7713 | 32.1857 |
| A15 | L-Phenylalanine | 17.4628 | 13.0302 | 10.9075 | 16.4981 | 24.7823 | 21.1847 | 35.6369 | 33.2053 | 63.0477 | 33.6388 | 27.1583 | 27.5966 |
| A16 | L-Proline | 41.9171 | 46.1434 | 55.4110 | 48.7888 | 42.8189 | 60.0032 | 366.4523 | 285.8890 | 401.8643 | 331.4673 | 341.6373 | 362.4342 |
| A17 | L-Serine | 274.3984 | 446.7053 | 394.5321 | 430.4091 | 397.1701 | 379.2513 | 576.6683 | 412.9500 | 649.5060 | 506.0223 | 465.5659 | 507.0126 |
| A18 | L-Threonine | 218.2402 | 325.3414 | 321.7773 | 328.4677 | 299.7062 | 295.1779 | 473.5417 | 295.8860 | 542.4701 | 413.6560 | 389.5629 | 391.7109 |
| A19 | L-Tryptophan | 332.7943 | 470.9194 | 395.9716 | 562.2661 | 417.4273 | 445.1639 | 2901.4103 | 1645.2600 | 3593.8024 | 2312.4334 | 2121.8038 | 2201.9359 |
| A20 | L-Tyrosine | 78.1454 | 113.8044 | 105.6358 | 80.0767 | 111.2957 | 110.4938 | 682.7185 | 342.8760 | 716.2355 | 477.5725 | 467.1586 | 477.8518 |
| A21 | L-Valine | 43.0141 | 46.5771 | 44.8714 | 41.1187 | 47.5647 | 44.8313 | 129.6149 | 70.1732 | 140.8234 | 101.8285 | 93.9115 | 95.0934 |
| A22 | Sarcosine | N/A | N/A | N/A | N/A | N/A | N/A | 0.5938 | 0.4336 | 0.5405 | 0.4604 | 0.7891 | 0.5690 |
| A23 | L-Ornithine | 51.9747 | 61.1162 | 53.7919 | 64.0483 | 59.8542 | 60.2290 | 231.1402 | 163.8050 | 237.9341 | 223.5544 | 254.1249 | 272.7693 |
| A24 | L-Citrulline | 90.5565 | 558.0424 | 544.5996 | 563.0725 | 570.8793 | 430.8876 | 10210.2420 | 7317.0500 | 11423.7525 | 9489.9447 | 9851.3677 | 10443.5928 |
| A25 | L-Homocitrulline | 0.9603 | 1.8047 | 1.6323 | 1.7147 | 1.5464 | 1.5109 | 3.0904 | 2.0634 | 3.3845 | 2.4290 | 2.4272 | 2.4413 |
| A26 | Homo-L-arginine | 70.4145 | 92.9082 | 94.2663 | 84.6635 | 78.9974 | 76.4014 | 177.3515 | 127.8430 | 174.8134 | 140.8723 | 136.4480 | 144.1382 |
| A27 | S-(5-Adenosyl)-L-Homocysteine | 2.4579 | 1.8351 | 1.8288 | 1.3986 | 2.1018 | 1.5892 | 1.6578 | 1.0258 | 2.1416 | 1.1946 | 1.3762 | 1.6820 |
| A28 | Nα-Acetyl-L-glutamine | 2.1366 | 3.9812 | 4.0633 | 4.8056 | 4.4800 | 5.0244 | 2.1093 | 2.3275 | 2.4322 | 2.1058 | 1.8610 | 2.2772 |
| A29 | Nicotinuric Acid | 0.4418 | 0.6275 | 0.5599 | 0.5354 | 0.5230 | 0.6423 | 0.4586 | 0.4088 | 0.4313 | 0.4073 | 0.4135 | 0.4207 |
| A30 | L-tyrosine methyl ester | 0.3751 | 0.4448 | 0.3802 | 0.2648 | 0.2565 | 0.2545 | 0.4175 | 0.1472 | 0.4697 | 0.1864 | 0.1951 | 0.2750 |
| A31 | Glycyl-L-Proline | 0.3897 | 0.4636 | 0.4584 | 0.4766 | 0.4684 | 0.4742 | 0.5691 | 0.4290 | 0.5715 | 0.5124 | 0.4890 | 0.4753 |
| A32 | Glutathione Oxidized | 4553.0726 | 4545.4375 | 6198.0330 | 5552.4727 | 5302.5816 | 4730.4878 | 15194.0388 | 9095.2500 | 23521.8563 | 11268.5021 | 11766.1051 | 13842.9638 |
| A33 | argininosuccinic acid | 13.5383 | 44.7295 | 31.7769 | 34.0429 | 55.4970 | 30.7879 | 32.7637 | 35.9022 | 64.6443 | 35.8430 | 43.6479 | 48.0440 |
| A34 | D-Alanyl-D-Alanine | 0.6150 | 0.8392 | 0.5596 | 0.4967 | 0.7829 | 0.6370 | 0.7947 | 0.3834 | 0.6865 | 0.5066 | 0.4958 | 0.5137 |
| A35 | Trimethylamine N-Oxide | 0.0153 | 0.5359 | 0.2839 | 0.6001 | 0.3709 | 0.5852 | 0.0122 | 0.0089 | 0.0238 | 0.0095 | 0.0121 | 0.0108 |
| A36 | Trans-4-Hydroxy-L-Proline | 3.1887 | 4.1729 | 4.5471 | 3.8991 | 3.7522 | 3.4723 | 49.2898 | 19.9303 | 40.7426 | 27.1605 | 29.8881 | 25.9574 |
| A37 | Succinic Acid | 1578.0926 | 2001.9101 | 1447.6257 | 1336.6236 | 1580.5836 | 1422.4310 | 1080.1360 | 791.6640 | 1467.2156 | 930.7253 | 916.3211 | 1315.4481 |
| A38 | glycylphenylalanine | 0.5768 | 0.7259 | 0.6958 | 0.6711 | 0.6762 | 0.6845 | 0.6026 | 0.4746 | 0.6262 | 0.5151 | 0.5404 | 0.5580 |
| A39 | γ-Glutamate-Cysteine | 36.2309 | N/A | N/A | 12.7890 | N/A | 13.6062 | 175.1230 | 190.7960 | 141.1816 | 191.0144 | 175.3806 | 197.5757 |
| A40 | S-Sulfo-L-Cysteine | 3.1623 | 8.6376 | 5.9319 | 6.9580 | 6.6150 | 5.3032 | N/A | N/A | 0.3298 | N/A | N/A | N/A |
| A41 | 5-Hydroxy-Tryptamine | N/A | 0.9594 | 0.3303 | 1.8007 | 0.6240 | 2.5424 | N/A | N/A | N/A | N/A | N/A | N/A |
| A42 | Urea | N/A | 2.3888 | 4.3234 | 5.2276 | 1.4930 | 2.7638 | N/A | N/A | N/A | N/A | N/A | N/A |
| A43 | N-Propionylglycine | N/A | 0.2917 | 0.2944 | 0.3110 | 0.2848 | 0.2908 | N/A | N/A | N/A | N/A | N/A | N/A |
| A44 | L-Carnosine | 0.9822 | 1.1718 | 1.0639 | 1.0320 | 1.1715 | 1.1053 | N/A | N/A | N/A | N/A | N/A | N/A |
| A45 | Homoserine | N/A | N/A | N/A | N/A | N/A | N/A | 44.5883 | 30.8080 | 42.5759 | 27.1550 | 28.6430 | 28.2207 |
| A46 | (5-L-Glutamyl)-L-Amino Acid | 61.5194 | 172.0734 | 169.6443 | 209.8064 | 122.5381 | 163.5676 | 113.1586 | 109.2820 | 149.4212 | 82.6683 | 100.5292 | 91.4133 |
| A47 | N6-Acetyl-L-Lysine | 5.7107 | 6.0168 | 5.8566 | 5.2458 | 5.5178 | 5.0722 | 20.5002 | 9.1138 | 24.2895 | 13.2769 | 13.1725 | 12.4534 |
| A48 | Nα-Acetyl-L-Arginine | 1.6506 | 3.1876 | 3.6774 | 3.7762 | 2.4901 | 5.2896 | 2.5656 | 1.9488 | 2.9350 | 2.6038 | 3.5750 | 2.7998 |
| A49 | D-Homocysteine | 7.6005 | 13.3918 | 5.4607 | 5.7658 | 9.4425 | 6.9191 | 2.6540 | 2.3395 | 2.9713 | 2.6928 | 2.6168 | 2.8404 |
| A50 | Beta-Alanine | 12.1690 | 16.5401 | 17.0912 | 16.0713 | 15.3661 | 13.6810 | 1048.5997 | 433.2040 | 1153.8224 | 615.6197 | 665.9485 | 695.6063 |
| A51 | L-α-Aspartyl-L-phenylalanine | 0.2113 | 0.4647 | 0.3359 | 0.3737 | 0.4951 | 0.5290 | 0.4105 | 0.1751 | 0.4746 | 0.3201 | 0.2439 | 0.2958 |
| A52 | 5-Hydroxylysine | 0.4964 | 0.6377 | 0.7489 | 0.6074 | 0.6582 | 0.5691 | 1.8612 | 1.4306 | 2.3813 | 1.6109 | 1.9156 | 1.9444 |
| A53 | 3-N-Methyl-L-Histidine | 1.3432 | 1.3789 | 1.2180 | 1.1446 | 1.2281 | 1.1988 | 11.1178 | 9.2596 | 12.7008 | 8.7663 | 8.8714 | 9.2469 |
| A54 | 3-Aminoisobutanoic Acid | 112.5160 | 97.6855 | 64.0549 | 87.5673 | 114.2483 | 78.3895 | 186.0702 | 107.9500 | 184.2465 | 145.4292 | 129.9752 | 139.8634 |
| A55 | 1-Methylhistidine | 17.1940 | 21.0914 | 20.1852 | 18.7163 | 18.9310 | 16.5997 | 10.3747 | 7.4795 | 10.7106 | 9.1075 | 8.7028 | 8.0590 |
| A56 | N-Glycyl-L-Leucine | 0.7787 | 1.3673 | 1.0432 | 1.2320 | 1.1423 | 1.2358 | 1.6780 | 0.8476 | 1.7065 | 1.2202 | 1.2961 | 1.0612 |
| A57 | N-Acetylneuraminic Acid | 8.7779 | 6.4247 | 5.7389 | 5.6530 | 7.5717 | 7.0249 | 5.6063 | 6.6509 | 8.4557 | 4.9855 | 5.9428 | 5.0661 |
| A58 | N-Acetyl-L-Tyrosine | 0.2735 | 0.5051 | 0.4363 | 0.4031 | 0.4114 | 0.4723 | 0.2750 | 0.2527 | 0.2545 | 0.2475 | 0.2416 | 0.2538 |
| A59 | Methionine Sulfoxide | 1.5073 | 2.4551 | 2.4605 | 2.6502 | 2.1366 | 2.2342 | 3.3268 | 2.2977 | 3.6530 | 2.9919 | 2.8427 | 2.5182 |
| A60 | L-Theanine | 0.4633 | 0.5940 | 0.5561 | 0.5961 | 0.5565 | 0.5155 | 61.1946 | 15.7050 | 65.4071 | 27.0335 | 27.3005 | 31.2208 |
| A61 | L-Pipecolic Acid | 30.6588 | 49.0887 | 36.0024 | 49.2505 | 35.1028 | 32.9890 | 86.2756 | 39.9976 | 95.0738 | 70.4829 | 54.5008 | 58.8179 |
| A62 | L-Tryptophyl-L-glutamic acid | 15.3229 | 19.6769 | 22.4210 | 17.6191 | 27.2684 | 16.3082 | 12.1898 | 8.5494 | 20.3405 | 11.1017 | 9.3983 | 10.3723 |
| A63 | Creatine | 0.3809 | 0.5056 | 0.5058 | 0.6140 | 0.5830 | 0.4864 | 0.6124 | 0.9703 | 0.4324 | 0.5600 | 0.7074 | 0.6326 |
| A64 | (S)-β-Aminoisobutyric Acid | 0.8679 | 1.9969 | 1.9388 | 2.3064 | 2.0953 | 1.8188 | 47.5449 | 22.2727 | 48.6278 | 31.4411 | 30.1104 | 32.7598 |
| A65 | Kinurenine | 0.4322 | 0.4582 | 0.4567 | 0.4859 | 0.4476 | 0.4692 | 0.6859 | 0.6115 | 0.6686 | 0.6598 | 0.6284 | 0.6742 |
| A66 | 2-Aminobutyric acid | 0.7455 | 1.3560 | 0.9780 | 0.8679 | 0.8508 | 0.9882 | 5.6197 | 3.6381 | 6.1152 | 4.4139 | 4.6077 | 4.5102 |
| A67 | 2-Aminoethanesulfonic Acid | 0.4219 | 0.4778 | 0.5776 | 0.5229 | 0.5080 | 0.5958 | 0.5128 | 0.4393 | 0.4788 | 0.3745 | 0.5645 | 0.4432 |
| A68 | 4-Acetamidobutyric Acid | 1.1061 | 3.2731 | 1.8428 | 2.1016 | 1.8229 | 2.2681 | 0.6829 | 0.5220 | 0.7965 | 0.5563 | 0.5727 | 0.6244 |
| A69 | 5-Aminovaleric Acid | 0.5695 | 0.7944 | 0.6674 | 0.6696 | 0.6861 | 0.7042 | 2.7248 | 2.5147 | 3.4668 | 1.5105 | 2.7394 | 3.7013 |
| A70 | γ-Aminobutyric Acid | 658.7370 | 609.5408 | 540.6130 | 348.3843 | 564.1998 | 475.2269 | 688.3597 | 553.5830 | 843.4581 | 700.8368 | 694.1556 | 750.2211 |
| A71 | Kynurenic Acid | 0.0253 | 0.0753 | 0.0723 | 0.0812 | 0.0528 | 0.0805 | 0.1294 | 0.0685 | 0.1536 | 0.0804 | 0.0933 | 0.1176 |
| A72 | 6-Aminocaproic Acid | 0.4854 | 0.6421 | 0.6710 | 0.4381 | 0.5047 | 0.5443 | 0.5017 | 0.4233 | 0.5984 | 0.4643 | 0.5169 | 0.5496 |
| A73 | 1,3,7-Trimethyluric Acid | 0.0024 | 0.0124 | 0.0081 | 0.0083 | 0.0099 | 0.0113 | 0.0069 | 0.0036 | 0.0081 | 0.0033 | 0.0046 | 0.0056 |
| A74 | 3,7-Dimethyluric Acid | 0.0394 | 0.2403 | 0.8236 | 0.5545 | 0.2881 | 0.4253 | N/A | N/A | N/A | N/A | N/A | N/A |
| A75 | α-Aminoadipic acid | 41.5777 | 72.5999 | 62.6073 | 75.0362 | 55.6412 | 72.9553 | 10.4404 | 5.2477 | 11.1822 | 8.3836 | 8.5649 | 9.9766 |
| A76 | N'-Formylkynurenine | 18.3389 | 24.8636 | 23.9489 | 23.9477 | 21.9391 | 23.9720 | 77.9824 | 38.6891 | 78.4975 | 46.2871 | 51.0644 | 60.0439 |

**Note:** N/A means not detected or under limit of detection.

**Table S3** The retention times, multiple reaction monitoring parameters, calibration curves, correlation factors, linear ranges, LLOQs, and ULOQs of free fatty acids (FFAs) from the fresh bulbs of AMB and ACGD by GC-MS in positive ion mode.

| **No.** | **Compounds** | **Class** | **Formula** | **RT**  **(min)** | **Q1**  **(Da)** | **Calibration curves** | ***R*^2^** | **Linear range**  **(μg/mL)** | **LLOQ**  **(μg/mL)** | **ULOQ**  **(μg/mL)** |
| --- | --- | --- | --- | --- | --- | --- | --- | --- | --- | --- |
| C6-0 | hexanoic acid | lipid | C6H12O2 | 4.66 | 130.2 | Y = 1.1057e6 X - 3699.9892 | 0.9975 | 0.01-5.0 | 0.01 | 5.0 |
| C8-0 | octanoic acid | lipid | C8H16O2 | 5.88 | 158.4 | Y = 1.1094e4 X - 31.5696 | 0.9977 | 0.01-5.0 | 0.01 | 5.0 |
| C9-0 | nonanoic acid | lipid | C9H18O2 | 6.41 | 172.9 | Y = 1.9950e4 X - 90.1819 | 0.9987 | 0.01-5.0 | 0.01 | 5.0 |
| C10-0 | decanoic acid | lipid | C10H20O2 | 6.90 | 186.3 | Y = 3.2701e4 X - 54.2679 | 0.9975 | 0.01-5.0 | 0.01 | 5.0 |
| C11-0 | hendecanoic acid | lipid | C11H22O2 | 7.36 | 200.1 | Y = 1.1857e6 X + 553.7971 | 0.9974 | 0.01-1.0 | 0.01 | 1.0 |
| C12-0 | lauric acid | lipid | C12H24O2 | 7.82 | 214.7 | Y = 7.0002e4 X - 367.2356 | 0.9992 | 0.01-5.0 | 0.01 | 5.0 |
| C13-0 | tridecanoic acid | lipid | C13H26O2 | 8.35 | 228.4 | Y = 8.5370e4 X - 609.7076 | 0.9982 | 0.01-5.0 | 0.01 | 5.0 |
| C14-0 | myristic acid | lipid | C14H28O2 | 8.96 | 242.8 | Y = 1.0229e5 X - 719.9592 | 0.9988 | 0.01-5.0 | 0.01 | 5.0 |
| C15-0 | pentadecanoic acid | lipid | C15H30O2 | 9.64 | 256.5 | Y = 1.1603e5 X - 1012.1258 | 0.9983 | 0.01-5.0 | 0.01 | 5.0 |
| C15-1 | cis-10-pentadecenoic acid | lipid | C15H28O2 | 9.56 | 254.6 | Y = 1.9084e4 X - 225.9079 | 0.9962 | 0.01-2.0 | 0.01 | 2.0 |
| C16-0 | palmitic acid | lipid | C16H32O2 | 10.39 | 270.5 | Y = 1.1679e5 X - 930.9433 | 0.9995 | 0.02-10.0 | 0.02 | 10.0 |
| C16-1 | cis-9-palmitoleic acid | lipid | C16H30O2 | 10.24 | 268.7 | Y = 1.4111e4 X - 55.1942 | 0.9992 | 0.01-0.5 | 0.01 | 0.5 |
| C16-1T | trans-9-palmitelaidic acid | lipid | C17H32O2 | 10.27 | 268.9 | Y = 2.2282e4 X - 232.5478 | 0.9982 | 0.01-5.0 | 0.01 | 5.0 |
| C17-0 | heptadecanoic acid | lipid | C17H34O2 | 11.17 | 284.1 | Y = 1.2370e5 X - 1167.0049 | 0.9994 | 0.01-10.0 | 0.01 | 10.0 |
| C18-0 | stearic acid | lipid | C18H36O2 | 11.99 | 298.3 | Y = 1.3589e5 X - 1254.6983 | 0.9976 | 0.01-2.0 | 0.01 | 2.0 |
| C18-1n9c | cis-9-octadecenoic acid | lipid | C18H34O2 | 11.79 | 296.6 | Y = 1.6371e4 X - 170.4149 | 0.9995 | 0.01-5.0 | 0.01 | 5.0 |
| C18-1n9t | trans-9-octadecenoic acid | lipid | C18H34O2 | 11.83 | 296.2 | Y = 1.7172e4 X - 169.7636 | 0.9952 | 0.01-2.0 | 0.01 | 2.0 |
| C18-2n6c | linoleic acid (LA) | lipid | C18H32O2 | 11.73 | 294.8 | Y = 4.7013e4 X - 2275.7447 | 0.9996 | 0.05-10.0 | 0.05 | 10.0 |
| C18-2n6t | linolelaidic acid | lipid | C18H32O2 | 11.79 | 294.4 | Y = 5.6662e4 X - 652.4345 | 0.9938 | 0.01-5.0 | 0.01 | 5.0 |
| C18-3n3 | α-linolenic acid (α-LA) | lipid | C18H30O2 | 11.79 | 292.2 | Y = 2.1465e4 X - 254.4921 | 0.9993 | 0.01-5.0 | 0.01 | 5.0 |
| C18-3n6 | γ-linolenic acid (γ-LA) | lipid | C18H30O2 | 11.61 | 292.6 | Y = 2.2125e4 X - 1257.4628 | 0.9942 | 0.05-10.0 | 0.05 | 10.0 |
| C19-0 | nonadecylic acid | lipid | C19H38O2 | 12.83 | 312.1 | Y = 1.3149e5 X - 1537.0685 | 0.9967 | 0.01-2.0 | 0.01 | 2.0 |
| C20-0 | arachidic acid (AA) | lipid | C20H40O2 | 13.82 | 326.9 | Y = 1.3443e5 X - 1615.7594 | 0.9954 | 0.01-2.0 | 0.01 | 2.0 |
| C20-2 | cis-11,14-eicosadienoic acid | lipid | C20H36O2 | 13.51 | 322.1 | Y = 4.3470e4 X - 2545.5121 | 0.9965 | 0.05-10.0 | 0.05 | 10.0 |
| C20-4n6 | arachidonic acid (AHA) | lipid | C20H32O2 | 13.14 | 318.4 | Y = 2.5585e3 X - 104.3489 | 0.9995 | 0.05-10.0 | 0.05 | 10.0 |
| C20-5n3 | cis-5,8,11,14,17-eicosapentaenoic acid (EPA) | lipid | C20H30O2 | 13.20 | 316.3 | Y = 7.6925e2 X - 10.7263 | 0.9956 | 0.10-2.0 | 0.10 | 2.0 |
| C21-0 | heneicosanoic acid | lipid | C21H42O2 | 14.90 | 340.7 | Y = 1.3582e5 X - 6063.1213 | 0.9963 | 0.05-10.0 | 0.05 | 10.0 |
| C22-0 | behenic acid | lipid | C22H44O2 | 16.06 | 354.2 | Y = 1.2537e5 X - 5733.6360 | 0.9951 | 0.05-10.0 | 0.05 | 10.0 |
| C22-1n9 | erucic acid | lipid | C22H42O2 | 15.76 | 352.7 | Y = 8.3336e3 X - 413.1914 | 0.9963 | 0.05-10.0 | 0.05 | 10.0 |
| C22-5 (cis-7,10,13,16,19) | cis-7,10,13,16,19-docosapentaenoic acid (DPA) | lipid | C22H34O2 | 15.33 | 344 | Y = 1.0417e3 X - 67.0259 | 0.9994 | 0.10-5.0 | 0.10 | 5.0 |
| C22-6n3 | cis-4,7,10,13,16,19-docosahexaenoic acid (DHA) | lipid | C22H32O2 | 15.16 | 79.6 | Y = 4.0600e5 X - 49575.4658 | 0.9993 | 0.20-10.0 | 0.20 | 10.0 |
| C24-0 | lignoceric acid | lipid | C24H48O2 | 18.50 | 382.5 | Y = 9.6171e4 X - 5866.3637 | 0.9954 | 0.05-10.0 | 0.05 | 10.0 |

**Table S4** The qualitative and quantitative results of free fatty acids (FFAs) in AMB and ACGD by GC-MS.

| **No.** | **Compounds** | **Content levels (μg/g)** | | | | | | | | | | | |
| --- | --- | --- | --- | --- | --- | --- | --- | --- | --- | --- | --- | --- | --- |
|  |  | **AMB_1** | **AMB_2** | **AMB_3** | **AMB_4** | **AMB_5** | **AMB_6** | **ACGD_1** | **ACGD_2** | **ACGD_3** | **ACGD_4** | **ACGD_5** | **ACGD_6** |
| C6-0 | hexanoic acid | 0.0944 | 0.1213 | 0.1163 | 0.1007 | 0.1230 | 0.1039 | 0.1509 | 0.1097 | 0.1332 | 0.1233 | 0.1246 | 0.1113 |
| C8-0 | octanoic acid | 0.2302 | 0.1869 | 0.1717 | 0.1566 | 0.1893 | 0.1702 | 0.5512 | 0.4156 | 0.3319 | 0.3716 | 0.6987 | 0.5103 |
| C9-0 | nonanoic acid | 0.2202 | 0.3263 | 0.3921 | 0.2695 | 0.3156 | 0.2663 | 0.3159 | 0.3357 | 0.2624 | 0.2706 | 0.3047 | 0.2723 |
| C10-0 | decanoic acid | 0.1852 | 0.3902 | 0.3538 | 0.2901 | 0.3548 | 0.2126 | 0.2208 | 0.1671 | 0.1745 | 0.1670 | 0.2071 | 0.2130 |
| C11-0 | hendecanoic acid | 0.0281 | 0.0646 | 0.0531 | 0.0393 | 0.0505 | 0.0304 | 0.0605 | 0.0354 | 0.0357 | 0.0355 | 0.0471 | 0.0417 |
| C12-0 | lauric acid | 1.2020 | 1.6355 | 1.7861 | 1.6146 | 1.5398 | 1.8158 | 1.2055 | 0.8775 | 0.9525 | 0.9477 | 0.9909 | 1.1101 |
| C13-0 | tridecanoic acid | 0.2140 | 2.0380 | 1.0295 | 0.4945 | 2.3131 | 0.2835 | 0.9366 | 0.4296 | 0.7012 | 0.9143 | 0.9115 | 0.1791 |
| C14-0 | myristic acid | 4.2185 | 5.0072 | 5.3964 | 5.1060 | 4.4312 | 5.1763 | 3.6504 | 3.3544 | 2.8830 | 3.0505 | 3.7095 | 3.7561 |
| C15-0 | pentadecanoic acid | 2.8088 | 3.7269 | 3.6581 | 3.5942 | 3.4641 | 3.6686 | 2.5441 | 1.8527 | 2.2379 | 1.8553 | 1.9839 | 2.2017 |
| C15-1 | cis-10-pentadecenoic acid | 0.5221 | 0.5897 | 0.5330 | 0.5933 | 0.5741 | 0.5383 | 0.5501 | 0.5924 | 0.4040 | 0.5064 | 0.4483 | 0.5505 |
| C16-0 | palmitic acid | 272.0660 | 282.1103 | 273.6107 | 289.2736 | 276.1914 | 278.0174 | 286.7024 | 283.2086 | 262.5234 | 280.5211 | 276.9955 | 291.4784 |
| C16-1 | cis-9-palmitoleic acid | 3.4457 | 5.2124 | 5.1351 | 3.8250 | 4.8509 | 5.0978 | 3.8461 | 2.9049 | 2.6884 | 3.9451 | 3.4161 | 3.2893 |
| C16-1T | trans-9-palmitelaidic acid | 0.9696 | 1.1510 | 1.1371 | 1.1350 | 0.9388 | 0.7999 | 1.1120 | 0.8962 | 1.1207 | 0.9403 | 0.9590 | 1.0886 |
| C17-0 | heptadecanoic acid | 2.5800 | 3.9731 | 4.1802 | 4.1598 | 3.5089 | 4.1858 | 4.2002 | 2.5959 | 3.5385 | 2.9079 | 2.9317 | 3.2720 |
| C18-0 | stearic acid | 208.3035 | 217.4739 | 218.9850 | 219.6286 | 204.1922 | 215.4749 | 206.6270 | 206.2693 | 174.2352 | 173.4180 | 208.9998 | 210.6648 |
| C18-1n9c | cis-9-octadecenoic acid | 57.0725 | 49.2778 | 44.9341 | 37.2662 | 47.1131 | 42.2583 | 52.5649 | 34.2795 | 50.2002 | 55.6467 | 36.2512 | 34.0818 |
| C18-1n9t | trans-9-octadecenoic acid | 4.5889 | 6.1342 | 5.5493 | 4.6408 | 5.0636 | 5.1184 | 4.5172 | 3.2353 | 3.9572 | 4.2913 | 3.2819 | 3.2451 |
| C18-2n6c | linoleic acid (LA) | 282.8665 | 290.5793 | 290.2547 | 281.7974 | 297.4798 | 287.2896 | 274.3625 | 243.4368 | 266.3805 | 269.0764 | 253.6301 | 267.1289 |
| C18-2n6t | linolelaidic acid | 1.7870 | 1.9796 | 1.6661 | 1.7894 | 1.8874 | 1.9407 | 1.6422 | 1.2575 | 1.4446 | 1.4398 | 1.2672 | 1.3657 |
| C18-3n3 | α-linolenic acid (α-LA) | 32.4106 | 35.9196 | 37.2329 | 33.5000 | 35.6963 | 37.3039 | 17.8947 | 10.2474 | 15.4115 | 14.9841 | 13.9232 | 16.0483 |
| C18-3n6 | γ-linolenic acid (γ-LA) | N/A | N/A | N/A | N/A | N/A | N/A | N/A | N/A | N/A | N/A | N/A | N/A |
| C19-0 | nonadecylic acid | 0.4133 | 0.5511 | 0.5694 | 0.5872 | 0.5451 | 0.5952 | 0.5345 | 0.4445 | 0.4852 | 0.4146 | 0.4671 | 0.4475 |
| C20-0 | arachidic acid (AA) | 2.8653 | 4.1505 | 4.3410 | 4.0731 | 3.5867 | 4.6213 | 2.4932 | 2.2445 | 2.0548 | 2.0410 | 2.4012 | 2.4405 |
| C20-2 | cis-11,14-eicosadienoic acid | 1.7778 | 1.8569 | 1.8251 | 1.8287 | 1.8612 | 1.7902 | 1.8453 | 1.6920 | 1.8341 | 1.7300 | 1.6212 | 1.6534 |
| C20-4n6 | arachidonic acid (AHA) | 0.5668 | 0.5881 | 0.5781 | 0.5955 | 0.5990 | 0.5656 | N/A | N/A | 0.5390 | N/A | N/A | N/A |
| C20-5n3 | cis-5,8,11,14,17-eicosapentaenoic  acid (EPA) | 2.2875 | 2.5573 | 2.5507 | 2.2020 | 3.1959 | 2.5770 | 4.2016 | 1.8624 | 2.4613 | 2.5714 | 2.1872 | 2.9286 |
| C21-0 | heneicosanoic acid | 1.3524 | 1.5830 | 1.5717 | 1.5821 | 1.6373 | 1.6203 | 1.2419 | 1.1301 | 1.1731 | 1.1283 | 1.1100 | 1.1335 |
| C22-0 | behenic acid | 8.3748 | 9.8746 | 9.1690 | 8.2843 | 9.4350 | 10.3336 | 4.7448 | 3.5207 | 4.1969 | 4.0803 | 3.6740 | 3.7615 |
| C22-1n9 | erucic acid | 2.9968 | 4.6627 | 3.7878 | 3.6922 | 3.2564 | 3.3200 | 3.4812 | 10.1187 | 3.2889 | 2.3618 | 5.5905 | 3.0994 |
| C22-5  (cis-7,10,13,16,19) | cis-7,10,13,16,19-docosapentaenoic  acid (DPA) | 4.6502 | 6.4097 | 6.2881 | 6.0786 | 6.4679 | 6.7949 | 4.2092 | 3.1802 | 3.6213 | 3.2990 | 3.3315 | N/A |
| C22-6n3 | cis-4,7,10,13,16,19-docosahexaenoic  acid (DHA) | 2.5579 | 2.6368 | 2.6282 | 2.7420 | 2.7099 | 2.5923 | 2.5860 | 2.6473 | 2.5078 | 2.4990 | 2.5599 | 2.6165 |
| C24-0 | lignoceric acid | 12.9152 | 15.4848 | 14.5206 | 13.4626 | 14.4588 | 16.5459 | 8.1001 | 5.8688 | 6.9283 | 6.4547 | 6.6173 | 6.7342 |

**Note:** N/A means not detected or under limit of detection.

**Table S5** The retention times, multiple reaction monitoring parameters, calibration curves, correlation factors, linear ranges, LLOQs, and ULOQs of carotenoids from the fresh bulbs of AMB and ACGD by UHPLC/QTRAP-MS in positive ion mode.

| **No.** | **Compounds** | **Class** | **Formula** | **RT**  **(min)** | **Q1**  **(Da)** | **Q3**  **(Da)** | **Calibration curves** | ***R*^2^** | **Linear range**  **(μg/mL)** | **LLOQ**  **(μg/mL)** | **ULOQ**  **(μg/mL)** |
| --- | --- | --- | --- | --- | --- | --- | --- | --- | --- | --- | --- |
| Carotenoid_01 | α-carotene | carotenes | C40H56 | 5.92 | 537.5 | 123.2 | Y = 3.4628e5 X + 1083.9978 | 0.9946 | 0.05-250.0 | 0.05 | 250.0 |
| Carotenoid_02 | β-carotene | carotenes | C40H56 | 6.28 | 537.6 | 177.1 | Y = 6.9139e5 X - 1853.6023 | 0.9995 | 2.0-250.0 | 2.0 | 250.0 |
| Carotenoid_03 | γ-carotene | carotenes | C40H56 | 7.39 | 537.4 | 177.3 | Y = 5.6544e5 X - 18140.0533 | 0.9913 | 0.5-250.0 | 0.5 | 250.0 |
| Carotenoid_04 | ε-carotene | carotenes | C40H56 | 5.53 | 537.6 | 123.2 | Y = 1.2110e6 X - 1467.9099 | 0.9941 | 0.01-25.0 | 0.01 | 25.0 |
| Carotenoid_05 | Zeaxanthin  dipalmitate | xanthophylls | C72H116O4 | 7.95 | 789.5 | 533.5 | Y = 2.7975e6 X + 4137.5392 | 0.9963 | 0.5-5.0 | 0.5 | 5.0 |
| Carotenoid_06 | antheraxanthin | xanthophylls | C40H56O3 | 2.86 | 585.5 | 175.4 | Y = 5.7920e5 X + 313.4970 | 0.9994 | 0.05-125.0 | 0.05 | 125.0 |
| Carotenoid_07 | zeaxanthin | xanthophylls | C40H56O2 | 4.65 | 569.4 | 477.5 | Y = 2.9915e5 X - 731.1238 | 0.9992 | 0.1-50.0 | 0.1 | 50.0 |
| Carotenoid_08 | violaxanthin | xanthophylls | C40H56O4 | 1.56 | 601.4 | 221.0 | Y = 3.2417e6 X - 980.4862 | 0.9994 | 0.1-100.0 | 0.1 | 100.0 |
| Carotenoid_09 | lutein | xanthophylls | C40H56O2 | 4.07 | 551.5 | 175.4 | Y = 6.9935e5 X + 287.4061 | 0.9996 | 2.0-250.0 | 2.0 | 250.0 |
| Carotenoid_10 | canthaxanthin | xanthophylls | C40H52O2 | 4.76 | 565.5 | 203.3 | Y = 3.7730e7 X - 10.8917 | 0.9970 | 0.005-100.0 | 0.005 | 100.0 |
| Carotenoid_11 | echinenone | xanthophylls | C40H54O | 5.55 | 551.6 | 203.1 | Y = 1.6903e7 X - 1226.1334 | 0.9955 | 0.005-50.0 | 0.005 | 50.0 |
| Carotenoid_12 | neoxanthin | xanthophylls | C40H56O4 | 1.94 | 601.4 | 565.5 | Y = 1.6280e6 X - 189.2586 | 0.9994 | 0.01-50.0 | 0.01 | 50.0 |

**Table S6** The qualitative and quantitative results of carotenoids in AMB and ACGD by UHPLC/QTRAP-MS.

| **No.** | **Compounds** | **Class** | **RT (min)** | **Formula** | **Q1**  **(Da)** | **Q3**  **(Da)** | **Content levels (μg/g)** | | | | | | | | | | | |
| --- | --- | --- | --- | --- | --- | --- | --- | --- | --- | --- | --- | --- | --- | --- | --- | --- | --- | --- |
|  |  |  |  |  |  |  | **AMB**  **_1** | **AMB**  **_2** | **AMB**  **_3** | **AMB**  **_4** | **AMB**  **_5** | **AMB**  **_6** | **ACGD**  **_1** | **ACGD**  **_2** | **ACGD**  **_3** | **ACGD**  **_4** | **ACGD**  **_5** | **ACGD**  **_6** |
| Carotenoid_01 | α-carotene | carotenes | 5.92 | C40H56 | 537.5 | 123.2 | N/A | N/A | N/A | N/A | N/A | N/A | N/A | N/A | N/A | N/A | N/A | N/A |
| Carotenoid_02 | β-carotene | carotenes | 6.28 | C40H56 | 537.6 | 177.1 | 0.0133 | 0.0595 | 0.0839 | 0.1364 | 0.1155 | 0.0873 | 0.0570 | 0.0643 | 0.0580 | 0.1118 | 0.0853 | 0.0749 |
| Carotenoid_03 | γ-carotene | carotenes | 7.39 | C40H56 | 537.4 | 177.3 | N/A | N/A | N/A | N/A | N/A | N/A | N/A | N/A | N/A | N/A | N/A | N/A |
| Carotenoid_04 | ε-carotene | carotenes | 5.53 | C40H56 | 537.6 | 123.2 | N/A | N/A | N/A | N/A | N/A | N/A | N/A | N/A | N/A | N/A | N/A | N/A |
| Carotenoid_05 | Zeaxanthin  dipalmitate | xanthophylls | 7.95 | C72H116O4 | 789.5 | 533.5 | 0.0077 | N/A | 0.0132 | N/A | N/A | N/A | N/A | N/A | N/A | N/A | N/A | N/A |
| Carotenoid_06 | antheraxanthin | xanthophylls | 2.86 | C40H56O3 | 585.5 | 175.4 | 0.0349 | 0.0527 | 0.0533 | 0.0681 | 0.0649 | 0.0625 | 0.0075 | 0.0040 | 0.0112 | 0.0079 | 0.0070 | 0.0094 |
| Carotenoid_07 | zeaxanthin | xanthophylls | 4.65 | C40H56O2 | 569.4 | 477.5 | 0.1523 | 0.1953 | 0.1505 | 0.1709 | 0.2194 | 0.2369 | N/A | N/A | N/A | N/A | N/A | N/A |
| Carotenoid_08 | violaxanthin | xanthophylls | 1.56 | C40H56O4 | 601.4 | 221.0 | 0.0275 | 0.0402 | 0.0596 | 0.0773 | 0.0562 | 0.0580 | 0.0604 | 0.0561 | 0.0958 | 0.0931 | 0.0795 | 0.0721 |
| Carotenoid_09 | lutein | xanthophylls | 4.07 | C40H56O2 | 551.5 | 175.4 | 0.2181 | 1.5274 | 1.8495 | 3.4596 | 2.6266 | 2.6845 | 2.0253 | 2.0897 | 2.1819 | 3.5549 | 2.8361 | 2.5550 |
| Carotenoid_10 | canthaxanthin | xanthophylls | 4.76 | C40H52O2 | 565.5 | 203.3 | 0.0001 | 0.0002 | 0.0012 | 0.0002 | 0.0005 | 0.0002 | 0.0003 | 0.0001 | 0.0001 | N/A | N/A | 0.0001 |
| Carotenoid_11 | echinenone | xanthophylls | 5.55 | C40H54O | 551.6 | 203.1 | N/A | N/A | 0.0012 | 0.0005 | 0.0008 | N/A | N/A | N/A | N/A | N/A | N/A | N/A |
| Carotenoid_12 | neoxanthin | xanthophylls | 1.94 | C40H56O4 | 601.4 | 565.5 | 0.0291 | 0.0863 | 0.0981 | 0.1586 | 0.1193 | 0.1114 | 0.1119 | 0.1095 | 0.0842 | 0.1828 | 0.1396 | 0.1309 |
| Carotenoid_13 | Zeaxanthin  -dilaurate | xanthophylls | 7.33 | C64H100O4 | 933.9 | 533.2 | 0.0095 | 0.0243 | 0.0128 | 0.0245 | 0.0266 | 0.0247 | N/A | N/A | N/A | N/A | N/A | N/A |
| Carotenoid_14 | Zeaxanthin  -laurate  -myristate | xanthophylls | 7.47 | C66H105O4 | 962.7 | 733.5 | 0.0063 | 0.0105 | 0.0089 | 0.0161 | 0.0071 | 0.0100 | N/A | N/A | N/A | N/A | N/A | N/A |
| Carotenoid_15 | Zeaxanthin  -dimyristate | xanthophylls | 7.62 | C68H108O4 | 990 | 761.8 | 0.0410 | 0.0427 | 0.0472 | 0.0647 | 0.0588 | 0.0703 | N/A | N/A | N/A | N/A | N/A | N/A |
| Carotenoid_16 | Zeaxanthin  -laurate  -palmitate | xanthophylls | 7.64 | C68H108O4 | 989.9 | 533.4 | N/A | 0.0098 | 0.0081 | 0.0108 | 0.0078 | 0.0101 | N/A | N/A | N/A | N/A | N/A | N/A |
| Carotenoid_17 | Violaxanthin  -dibutyrate | xanthophylls | 6.23 | C48H68O6 | 741.6 | 653.5 | 0.0273 | 0.0469 | 0.0504 | 0.0828 | 0.0704 | 0.0856 | 0.0197 | 0.0140 | 0.0138 | 0.0213 | 0.0141 | 0.0162 |
| Carotenoid_18 | Violaxanthin  -laurate | xanthophylls | 5.67 | C52H80O6 | 783.7 | 583.4 | 0.0440 | 0.0393 | 0.0433 | 0.0497 | 0.0497 | 0.0739 | 0.0110 | 0.0092 | 0.0106 | 0.0108 | 0.0111 | 0.0116 |
| Carotenoid_19 | Violaxanthin  -dilaurate | xanthophylls | 6.62 | C64H101O6 | 966.7 | 948.8 | 0.0845 | 0.0982 | 0.1474 | 0.1283 | 0.1242 | 0.1321 | N/A | 0.0243 | N/A | 0.0256 | N/A | N/A |
| Carotenoid_20 | Violaxanthin  -myristate  -caprate | xanthophylls | 6.84 | C64H100O6 | 965.7 | 947.8 | 0.1446 | 0.1636 | 0.1806 | 0.1929 | 0.1894 | 0.2590 | N/A | 0.0685 | 0.0332 | 0.0375 | N/A | 0.0469 |
| Carotenoid_21 | Violaxanthin  -myristate  -laurate | xanthophylls | 6.85 | C66H104O6 | 993.8 | 975.7 | 0.1375 | 0.1152 | 0.1336 | 0.1681 | 0.1382 | 0.1647 | 0.0416 | 0.0409 | 0.0322 | 0.0322 | 0.0253 | N/A |
| Carotenoid_22 | Violaxanthin  -dimyristate | xanthophylls | 7.04 | C68H108O6 | 1021.8 | 793.7 | 0.0149 | N/A | N/A | 0.0135 | N/A | N/A | N/A | 0.0095 | N/A | N/A | N/A | N/A |
| Carotenoid_23 | Lutein caprate | xanthophylls | 5.99 | C50H74O3 | 705.7 | 533.5 | N/A | N/A | 0.0100 | 0.0161 | 0.0103 | 0.0092 | N/A | N/A | 0.0057 | 0.0090 | 0.0123 | 0.0103 |
| Carotenoid_24 | Lutein  -myristate | xanthophylls | 6.63 | C54H82O3 | 761.8 | 533.5 | 0.0062 | 0.0835 | 0.1232 | 0.2262 | 0.1273 | 0.1796 | 0.0766 | 0.0785 | 0.0736 | 0.0955 | 0.0819 | 0.1042 |
| Carotenoid_25 | Lutein  -palmitate | xanthophylls | 6.93 | C56H86O3 | 789.8 | 533.5 | N/A | N/A | 0.0185 | 0.0403 | 0.0228 | 0.0327 | N/A | N/A | N/A | N/A | N/A | N/A |
| Carotenoid_26 | Lutein stearate | xanthophylls | 6.9 | C58H90O3 | 817.8 | 533.5 | N/A | N/A | N/A | N/A | N/A | N/A | 0.0160 | 0.0074 | 0.0087 | 0.0068 | 0.0046 | 0.0072 |
| Carotenoid_27 | 5,6-epoxy  -lutein dilaurate | xanthophylls | 7.16 | C64H100O5 | 749.6 | 549.5 | N/A | N/A | 0.0126 | 0.0238 | 0.0090 | 0.0194 | N/A | N/A | N/A | N/A | N/A | 0.0062 |
| Carotenoid_28 | Lutein dilaurate | xanthophylls | 7.18 | C64H101O4 | 733.5 | 533.3 | 0.1902 | 0.6223 | 0.7510 | 1.2139 | 0.7098 | 0.7953 | 0.1208 | 0.1492 | 0.1138 | 0.1160 | 0.0978 | 0.1332 |
| Carotenoid_29 | Lutein  -dimyristate | xanthophylls | 7.34 | C68H108O4 | 761.8 | 533.5 | 0.0957 | 0.4439 | 0.6026 | 0.9810 | 0.5041 | 0.6972 | 0.1118 | 0.2089 | 0.1424 | 0.1681 | 0.1411 | 0.1832 |
| Carotenoid_30 | Lutein  -dipalmitate | xanthophylls | 7.59 | C72H116O4 | 789.8 | 533.5 | 0.0206 | 0.0572 | 0.0717 | 0.1234 | 0.0881 | 0.1138 | 0.0359 | 0.0352 | 0.0423 | 0.0357 | 0.0271 | 0.0358 |
| Carotenoid_31 | Β cryptoxanthin  -laurate | xanthophylls | 6.98 | C52H78O2 | 735.8 | 535.5 | 0.0153 | 0.0323 | 0.0218 | 0.0363 | N/A | 0.0292 | N/A | 0.0054 | N/A | N/A | N/A | N/A |
| Carotenoid_32 | β cryptoxanthin  -myristate | xanthophylls | 7.22 | C54H82O2 | 763.9 | 535.5 | 0.0104 | 0.0171 | 0.0323 | 0.0365 | 0.0175 | 0.0207 | N/A | 0.0082 | 0.0129 | N/A | N/A | 0.0095 |

**Note:** N/A means not detected or under limit of detection.

**Table S7** The retention times, multiple reaction monitoring parameters, calibration curves, correlation factors, linear ranges, LLOQs, and ULOQs of vitamins from the fresh bulbs of AMB and ACGD by UHPLC/TQS-MS in positive and negative ion mode.

| **No.** | **Compounds** | **Class** | **Formula** | **RT**  **(min)** | **Q1**  **(Da)** | **Q3**  **(Da)** | **Calibration curves** | **R2** | **Linear range**  **(μg/mL)** | **LLOQ**  **(μg/mL)** | **ULOQ**  **(μg/mL)** |
| --- | --- | --- | --- | --- | --- | --- | --- | --- | --- | --- | --- |
| V1 | Vitamin A | fat-soluble vitamin | C20H30O | 1.83 | 269.3 | 93.0 | Y = 2.5254e-3 X + 0.0206 | 0.9963 | 0.05-2.5 | 0.05 | 2.5 |
| V2 | Vitamin D2 | fat-soluble vitamin | C28H44O2 | 1.98 | 413.4 | 112.9 | Y = 8.3727e-2 X + 0.0050 | 0.9949 | 0.0025-0.5 | 0.0025 | 0.5 |
| V3 | Vitamin D3 | fat-soluble vitamin | C27H44O2 | 1.94 | 401.4 | 365.2 | Y = 7.6148e-3 X + 0.0101 | 0.9921 | 0.0025-0.5 | 0.0025 | 0.5 |
| V4 | Vitamin E | fat-soluble vitamin | C29H50O2 | 3.02 | 431.3 | 165.2 | Y = 0.1796 X + 0.0078 | 0.9953 | 0.001-0.1 | 0.001 | 0.1 |
| V5 | Vitamin B1 (thiamine) | water-soluble vitamin | C12H17ClN4OS | 0.75 | 265.1 | 122.1 | Y = 3.0431 X + 0.3542 | 0.9954 | 0.001-0.1 | 0.001 | 0.1 |
| V6 | Vitamin B2 (riboflavin) | water-soluble vitamin | C17H20N4O6 | 2.96 | 377.3 | 243.1 | Y = 0.4785 X - 0.0904 | 0.9962 | 0.0005-0.05 | 0.0005 | 0.05 |
| V7 | Vitamin B3 (nicotinamide) | water-soluble vitamin | C6H5NO2 | 1.72 | 123.2 | 80.2 | Y = 0.3852 X + 0.9436 | 0.9941 | 0.005-0.5 | 0.005 | 0.5 |
| V8 | Vitamin B5 (pantothenic acid) | water-soluble vitamin | C9H17NO5 | 1.96 | 220.1 | 90.1 | Y = 0.1417 X - 0.0279 | 0.9996 | 0.002-0.2 | 0.002 | 0.2 |
| V9 | Vitamin B6 (pyridoxic acid) | water-soluble vitamin | C8H9NO4 | 1.95 | 183.9 | 147.9 | Y = 0.4001 X - 0.0335 | 0.9990 | 0.001-0.05 | 0.001 | 0.05 |
| V10 | BDC (pyridoxine) | water-soluble vitamin | C8H11O3N | 1.40 | 184.0 | 148.0 | Y = 2.4308 X - 0.0689 | 0.9995 | 0.0001-0.02 | 0.0001 | 0.02 |
| V11 | Vitamin B7 (biotin) | water-soluble vitamin | C10H16N2O3S | 2.78 | 244.9 | 227.0 | Y = 2.1564 X + 0.1395 | 0.9924 | 0.0005-0.05 | 0.0005 | 0.05 |
| V12 | Vitamin B12 (cobalamin) | water-soluble vitamin | C63H88CoN14O14P | 2.32 | 117.0 | 73.0 | Y = 1.0589 X - 0.4316 | 0.9954 | 0.001-0.1 | 0.001 | 0.1 |
| V13 | FA (folic acid) | water-soluble vitamin | C19H19N7O6 | 2.75 | 442.0 | 295.1 | Y = 1.0065 X - 0.0703 | 0.9921 | 0.0002-0.04 | 0.0002 | 0.04 |
| V14 | 5-MTHFA (5-methyl-tetrahydrofolate) | water-soluble vitamin | C20H25N7O6 | 2.25 | 460.2 | 313.1 | Y = 0.0578 X - 0.0052 | 0.9979 | 0.0005-0.05 | 0.0005 | 0.05 |
| V15 | Vitamin C | water-soluble vitamin | C6H8O6 | 0.61 | 175.0 |  | Y = 2.296e7 X -1023.5181 | 0.9989 | 0.05-6.0 | 0.05 | 6.0 |

**Table S8** The qualitative and quantitative results of vitamins in AMB and ACGD by UHPLC/QTRAP-MS.

| **NO.** | **Compounds** | **Content levels (μg/g)** | | | | | | | | | | | |
| --- | --- | --- | --- | --- | --- | --- | --- | --- | --- | --- | --- | --- | --- |
|  |  | **AMB_1** | **AMB_2** | **AMB_3** | **AMB_4** | **AMB_5** | **AMB_6** | **ACGD_1** | **ACGD_2** | **ACGD_3** | **ACGD_4** | **ACGD_5** | **ACGD_6** |
| V1 | Vitamin A | 0.1246 | 0.1726 | 0.1930 | 0.0910 | 0.2730 | 0.3570 | 0.4704 | 0.3306 | 0.1712 | 0.2490 | 0.1472 | 0.1762 |
| V2 | Vitamin D2 | 0.0246 | 0.0312 | 0.0344 | 0.0330 | 0.0246 | 0.0370 | 0.0424 | 0.0284 | 0.0326 | 0.0370 | 0.0344 | 0.0226 |
| V3 | Vitamin D3 | 0.0046 | 0.0064 | 0.0070 | 0.0092 | 0.0106 | 0.0058 | 0.0112 | 0.0066 | 0.0096 | 0.0204 | 0.0190 | 0.0168 |
| V4 | Vitamin E | 0.0026 | 0.0036 | 0.0048 | 0.0032 | 0.0024 | 0.0034 | 0.0064 | 0.0032 | 0.0030 | 0.0028 | 0.0038 | 0.0046 |
| V5 | Vitamin B1 (thiamine) | 0.4012 | 0.5300 | 0.3572 | 0.6128 | 0.9448 | 0.4340 | 0.9052 | 0.9928 | 0.6248 | 1.5860 | 1.0744 | 0.8980 |
| V6 | Vitamin B2 (riboflavin) | 0.3604 | 0.4101 | 0.3267 | 0.4114 | 0.4032 | 0.4517 | 0.1498 | 0.1332 | 0.1267 | 0.2094 | 0.1603 | 0.7503 |
| V7 | Vitamin B3 (nicotinamide) | 1.1760 | 1.4315 | 1.6840 | 1.8795 | 2.4260 | 1.2730 | 1.0790 | 2.2930 | 2.3765 | 2.6605 | 1.4825 | 1.7825 |
| V8 | Vitamin B5 (pantothenic acid) | 0.5876 | 0.4767 | 0.4321 | 0.4668 | 0.4701 | 0.5452 | 0.4026 | 0.3992 | 0.4388 | 0.4015 | 0.4290 | 0.4326 |
| V9 | Vitamin B6 (pyridoxic acid) | 0.0045 | 0.0041 | 0.0033 | 0.0039 | 0.0031 | 0.0051 | 0.0164 | 0.0169 | 0.0170 | 0.0186 | 0.0192 | 0.0233 |
| V10 | BDC (pyridoxine) | 0.1908 | 0.2178 | 0.2103 | 0.2128 | 0.2004 | 0.2840 | 0.0123 | 0.0135 | 0.0118 | 0.0158 | 0.0142 | 0.0198 |
| V11 | Vitamin B7 (biotin) | 0.0401 | 0.0409 | 0.0447 | 0.0400 | 0.0410 | 0.0372 | 0.0178 | 0.0176 | 0.0237 | 0.0230 | 0.0163 | 0.0158 |
| V12 | Vitamin B12 (cobalamin) | 0.0021 | 0.0021 | 0.0023 | 0.0022 | 0.0022 | 0.0023 | 0.0022 | 0.0023 | 0.0023 | 0.0026 | 0.0024 | 0.0024 |
| V13 | FA (folic acid) | 0.0704 | 0.0730 | 0.0890 | 0.0510 | 0.0253 | 0.0550 | 0.0672 | 0.0778 | 0.0970 | 0.0470 | 0.0546 | 0.1106 |
| V14 | 5-MTHFA (5-methyltetrahydrofolate) | 0.0546 | 0.1132 | 0.0508 | 0.0272 | 0.1084 | 0.0452 | 0.0646 | 0.0716 | 0.0790 | 0.0890 | 0.0592 | 0.0512 |
| V15 | Vitamin C | 31.5486 | 40.7813 | 28.7181 | 32.9232 | 34.8156 | 38.0201 | 21.7964 | 22.2424 | 26.3881 | 29.8655 | 26.7140 | 26.1596 |

**Note:** N/A means not detected or under limit of detection.

**Table S9** The retention times, multiple reaction monitoring parameters, calibration curves, correlation factors, linear ranges, LLOQs, and ULOQs of trace elements from the fresh bulbs of AMB and ACGD by ICP-MS.

| **No.** | **Compounds** | **Calibration curves** | ***R*^2^** | **Linear range**  **(μg/mL)** | **LLOQ**  **(μg/mL)** | **ULOQ**  **(μg/mL)** | **DL**  **(ppt)** | **BEC**  **(ppt)** |
| --- | --- | --- | --- | --- | --- | --- | --- | --- |
| E1 | Li | Y = 0.0012 X + 0.0006 | 0.9998 | 0.005-0.1 | 0.005 | 0.1 | 0.1698 | 0.4590 |
| E2 | Mg | Y = 0.0814 X + 0.0224 | 0.9996 | 5.0-100.0 | 5.0 | 100.0 | 0.0070 | 0.2753 |
| E3 | Ca | Y = 0.0014 X + 0.0188 | 0.9986 | 6.0-120.0 | 6.0 | 120.0 | 1.3789 | 13.2048 |
| E4 | V | Y = 0.0032 X + 0.0006 | 0.9990 | 0.0015-0.03 | 0.0015 | 0.03 | 0.1157 | 0.1806 |
| E5 | Cr | Y = 0.0006 X + 0.0008 | 0.9989 | 0.003-0.06 | 0.003 | 0.06 | 1.0356 | 1.2922 |
| E6 | Mn | Y = 0.0039 X + 0.0120 | 0.9983 | 0.00225-0.045 | 0.00225 | 0.045 | 0.9363 | 3.0724 |
| E7 | Fe | Y = 0.2331 X + 0.0514 | 0.9992 | 40.0-800.0 | 40.0 | 800.0 | 0.0739 | 0.2207 |
| E8 | Co | Y = 0.0066 X + 0.0016 | 0.9998 | 0.0025-0.02 | 0.0025 | 0.02 | 0.0719 | 0.2497 |
| E9 | Ni | Y = 0.0052 X + 0.0106 | 0.9997 | 0.005-0.04 | 0.005 | 0.04 | 0.2348 | 2.0370 |
| E10 | Cu | Y = 4.1190 X + 0.0324 | 0.9977 | 0.3-6.0 | 0.3 | 6.0 | 0.0012 | 0.0079 |
| E11 | Zn | Y = 1.9750 X + 3.4740 | 0.9970 | 0.6-12.0 | 0.6 | 12.0 | 0.0445 | 1.7591 |
| E12 | As | Y = 0.0010 X + 0.0002 | 0.9992 | 0.002-0.04 | 0.002 | 0.04 | 0.4087 | 0.2039 |
| E13 | Se | Y = 0.00009 X + 0.00014 | 0.9982 | 0.025-0.5 | 0.025 | 0.5 | 4.0714 | 1.5632 |
| E14 | Sr | Y = 0.0137 X + 0.0577 | 0.9996 | 0.005-0.1 | 0.005 | 0.1 | 0.3632 | 4.2268 |
| E15 | Mo | Y = 0.0140 X + 0.0009 | 0.9994 | 0.001-0.02 | 0.001 | 0.02 | 0.0514 | 0.0652 |
| E16 | Cd | Y = 0.00028 X + 0.00026 | 0.9988 | 0.001-0.02 | 0.001 | 0.02 | 0.3404 | 0.9069 |
| E17 | Ba | Y = 0.0005 X + 0.0026 | 0.9990 | 0.001-0.02 | 0.001 | 0.02 | 0.6379 | 5.3700 |
| E18 | Hg | Y = 0.00065 X + 0.00007 | 0.9997 | 0.0005-0.04 | 0.0005 | 0.04 | 0.0920 | 0.1106 |
| E19 | Tl | Y = 0.0060 X + 0.00009 | 0.9970 | 0.0006-0.012 | 0.0006 | 0.012 | 0.0034 | 0.0149 |
| E20 | Pb | Y = 0.0038 X + 0.0126 | 0.9998 | 0.005-0.4 | 0.005 | 0.4 | 0.1920 | 3.3288 |
| E21 | Bi | Y = 0.0061 X + 0.0012 | 0.9996 | 0.001-0.02 | 0.001 | 0.02 | 0.0025 | 0.2013 |

**Table S10** The qualitative and quantitative results of trace elements in AMB and ACGD by ICP-MS.

| **No.** | **Compounds** | **Content levels (μg/g)** | | | | | | | | | | | |
| --- | --- | --- | --- | --- | --- | --- | --- | --- | --- | --- | --- | --- | --- |
|  |  | **AMB_1** | **AMB_2** | **AMB_3** | **AMB_4** | **AMB_5** | **AMB_6** | **ACGD_1** | **ACGD_2** | **ACGD_3** | **ACGD_4** | **ACGD_5** | **ACGD_6** |
| E1 | Li | 3.6673 | 3.7480 | 3.0895 | 2.3513 | 2.3188 | 2.3680 | 3.0058 | 2.5738 | 3.0855 | 3.0910 | 2.7395 | 3.0885 |
| E2 | Mg | 10327.5000 | 9930.2500 | 6759.0000 | 10856.7500 | 10512.7500 | 9960.5000 | 11613.5000 | 14521.0000 | 13278.0000 | 12121.7500 | 13757.2500 | 11495.7500 |
| E3 | Ca | 46583.2500 | 27036.2500 | 16663.5000 | 26781.0000 | 31445.2500 | 27008.2500 | 16191.7500 | 18477.7500 | 17634.5000 | 15810.5000 | 18777.2500 | 14575.0000 |
| E4 | V | 0.5485 | 0.5428 | 0.2948 | 0.4185 | 0.3420 | 0.4113 | 0.3723 | 0.3215 | 0.4338 | 0.3725 | 0.4845 | 0.4400 |
| E5 | Cr | 10.5320 | 9.7883 | 6.4855 | 11.4115 | 11.6855 | 11.9375 | 9.3383 | 11.4473 | 10.3803 | 9.3238 | 11.1723 | 10.3055 |
| E6 | Mn | 98.9025 | 101.3550 | 68.7425 | 113.3050 | 106.0850 | 95.0925 | 180.8800 | 231.0175 | 211.5400 | 199.7250 | 208.4100 | 183.6300 |
| E7 | Fe | 434.0000 | 573.7500 | 699.5000 | 87.2500 | 600.7500 | 573.5000 | 545.2500 | 483.5000 | 497.5000 | 557.5000 | 497.5000 | 549.5000 |
| E8 | Co | 0.6598 | 0.7555 | 0.5600 | 0.8940 | 0.6460 | 0.7495 | 4.3943 | 0.7223 | 0.7758 | 0.5740 | 0.7205 | 0.5585 |
| E9 | Ni | 2.9560 | 2.7085 | 0.3445 | 3.6680 | 2.8825 | 2.6573 | 3.5258 | 5.1168 | 5.6603 | 3.4493 | 7.0453 | 4.5600 |
| E10 | Cu | 71.2500 | 67.5000 | 24.5000 | 45.0000 | 47.5000 | 29.0000 | 76.0000 | 90.0000 | 97.2500 | 70.7500 | 94.0000 | 61.7500 |
| E11 | Zn | 371.5000 | 324.2500 | 223.7500 | 332.0000 | 381.2500 | 339.2500 | 630.2500 | 682.5000 | 715.7500 | 614.2500 | 706.5000 | 583.7500 |
| E12 | As | 0.3748 | 0.6033 | 0.2418 | 0.3080 | 0.6160 | 0.3073 | 0.4958 | 1.1138 | 0.9245 | 0.9825 | 0.6715 | 0.6030 |
| E13 | Se | 6.2218 | 3.1805 | 0.4290 | 1.1918 | 1.2050 | 0.4710 | 3.3413 | 5.4375 | 1.1943 | 6.0755 | 2.5145 | 1.0818 |
| E14 | Sr | 76.6563 | 59.1188 | 41.2188 | 60.2388 | 57.6963 | 50.8688 | 60.4788 | 73.5763 | 74.9288 | 63.8263 | 73.0538 | 60.6213 |
| E15 | Mo | 1.2415 | 0.9283 | 0.5693 | 0.9553 | 1.2118 | 1.0610 | 1.4718 | 1.6113 | 1.5663 | 1.3623 | 1.4800 | 1.3035 |
| E16 | Cd | 0.4738 | 0.4110 | 0.2748 | 0.6323 | 0.2293 | 0.2143 | 1.7268 | 1.7340 | 2.2773 | 1.6758 | 2.3808 | 1.7445 |
| E17 | Ba | 11.6193 | 10.1115 | 5.7590 | 10.2060 | 10.9148 | 9.1258 | 60.2148 | 83.6248 | 73.2323 | 64.2898 | 78.4398 | 61.4023 |
| E18 | Hg | 0.1323 | 0.0628 | 0.0098 | 0.0095 | 0.0068 | 0.0353 | 0.2325 | 0.1823 | 0.1608 | 0.0798 | 0.0453 | 0.0243 |
| E19 | Tl | 0.0403 | 0.0380 | 0.0155 | 0.0438 | 0.0555 | 0.0545 | 0.2618 | 0.3013 | 0.2698 | 0.2528 | 0.3120 | 0.2400 |
| E20 | Pb | 1.4698 | 0.9390 | 0.3270 | 0.6083 | 1.7410 | 0.7470 | 1.6963 | 1.8825 | 2.2218 | 1.9450 | 2.0103 | 1.5488 |
| E21 | Bi | 0.0045 | 0.0185 | 0.0305 | 0.0115 | 0.0160 | 0.0218 | 0.0053 | 0.0038 | 0.0292 | 0.0110 | 0.0190 | 0.0223 |

**Note:** N/A means not detected or under limit of detection.


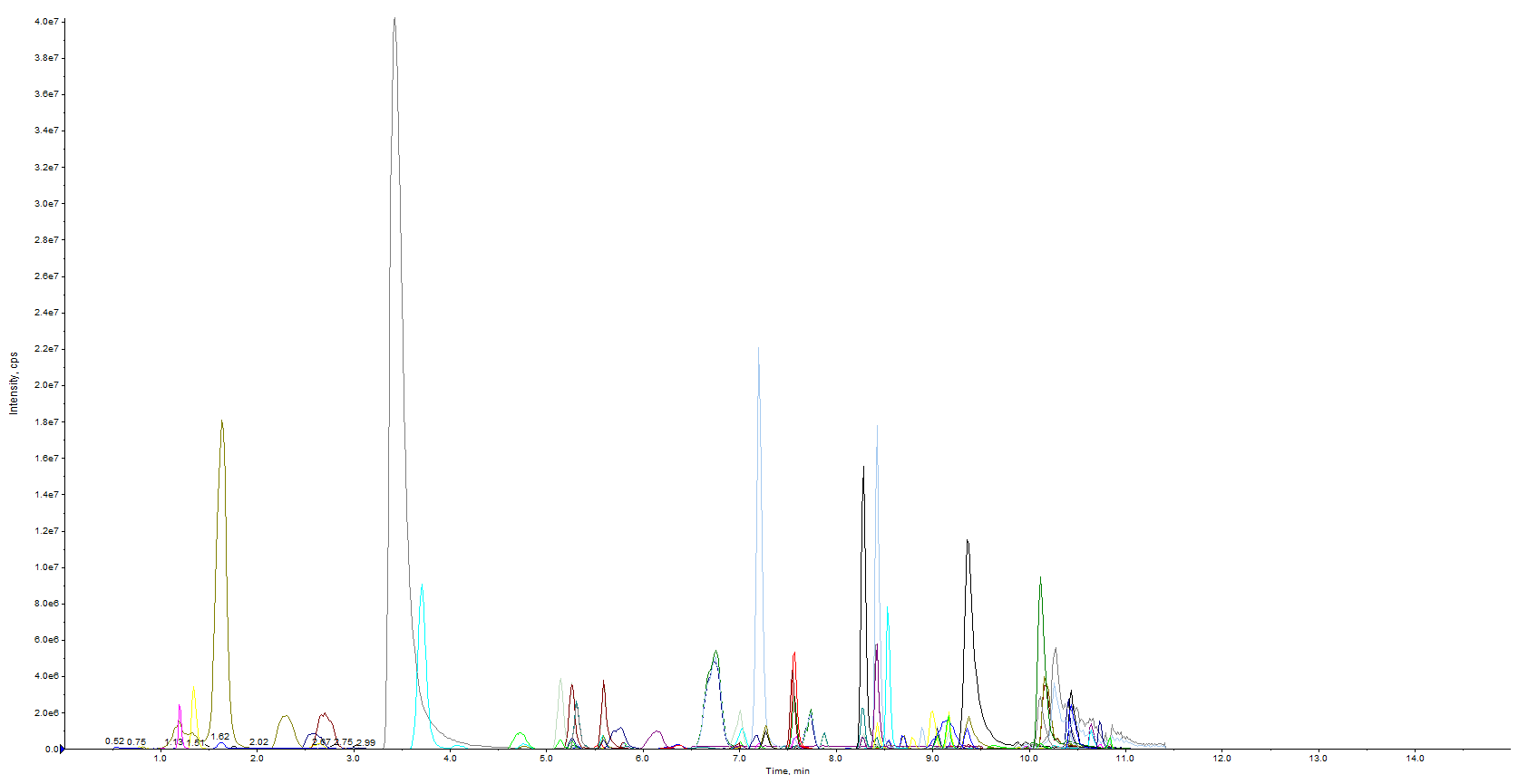


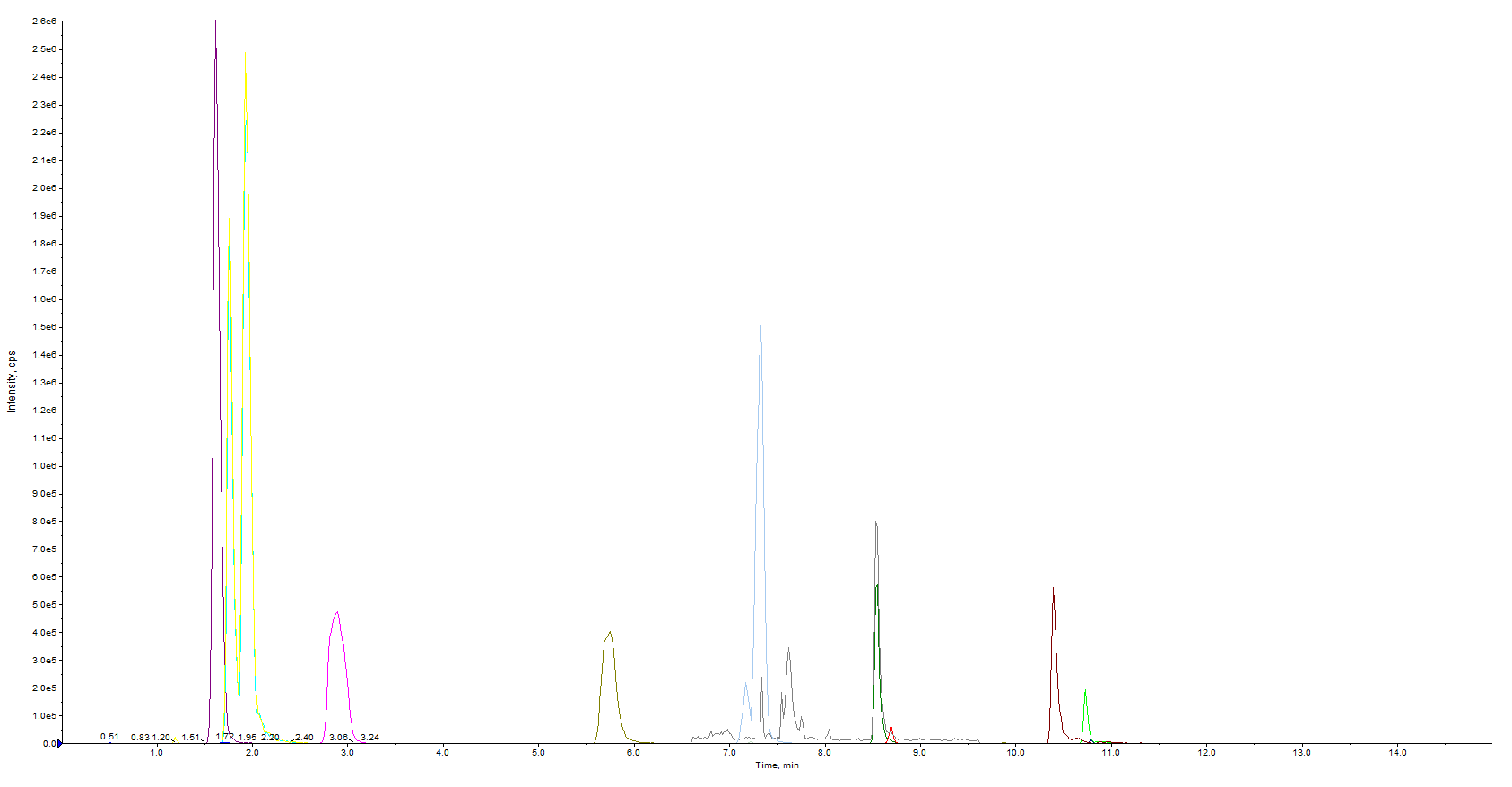


**Figure S1**


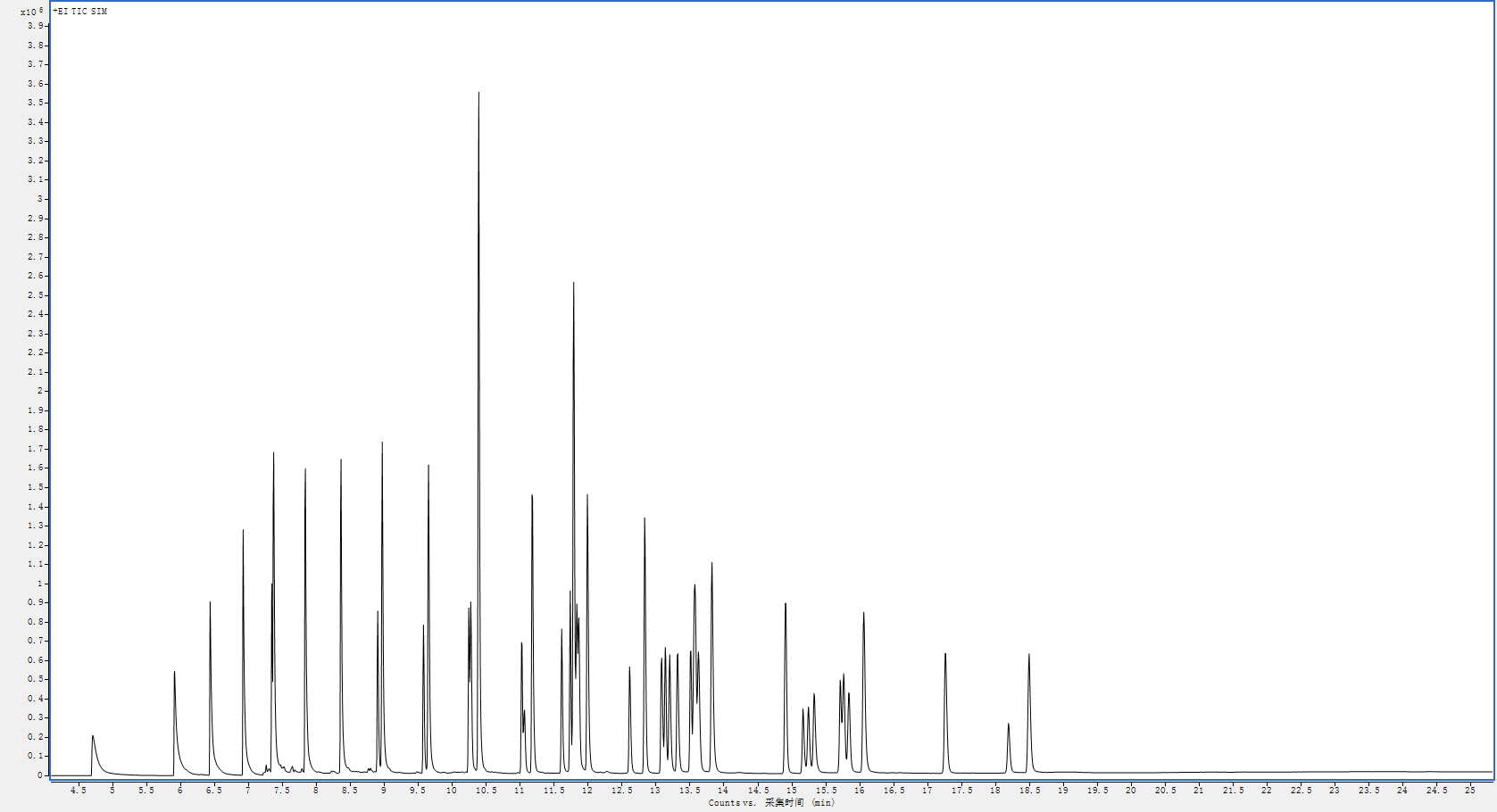


**Figure S2**


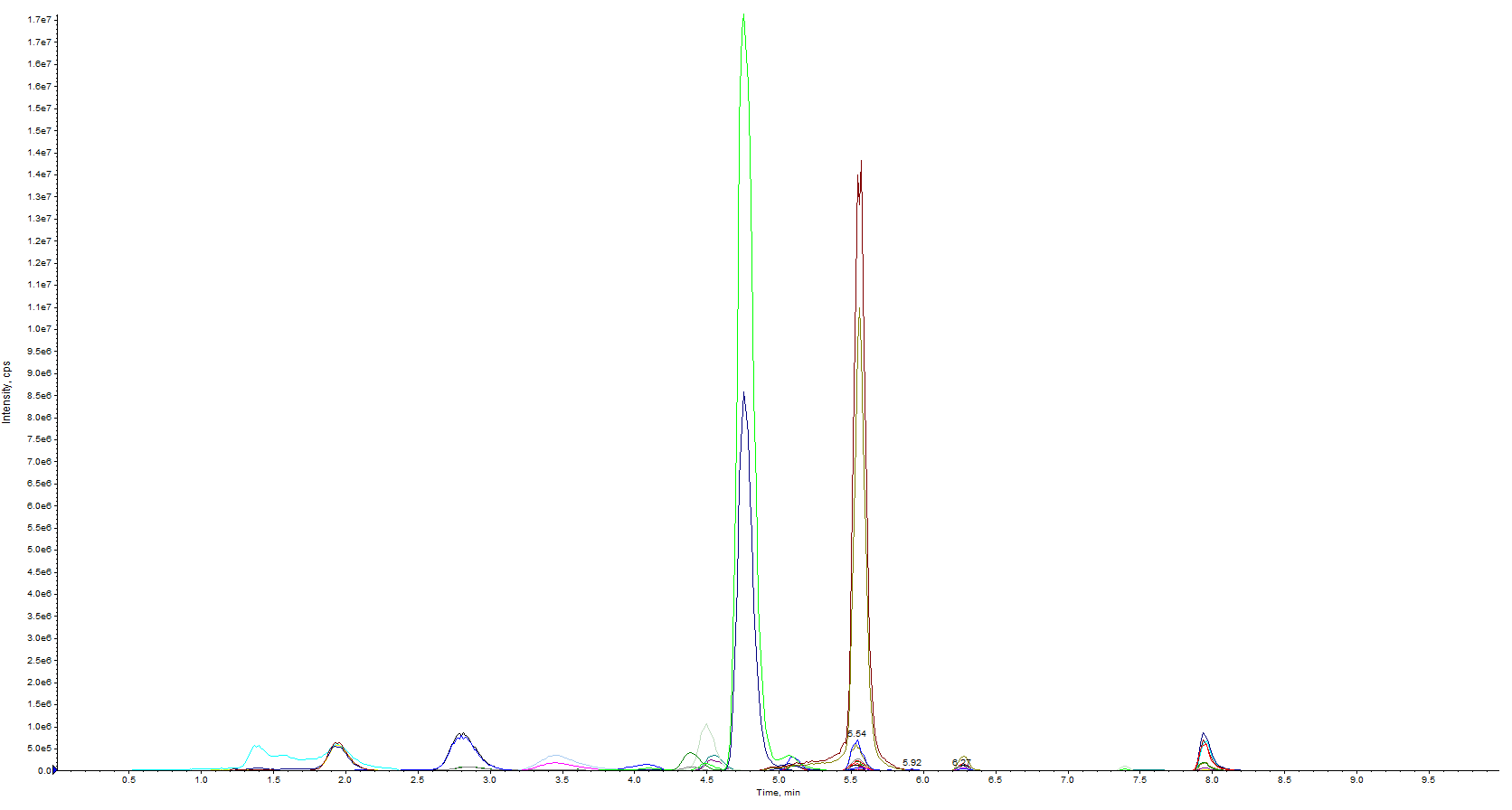


**Figure S3**


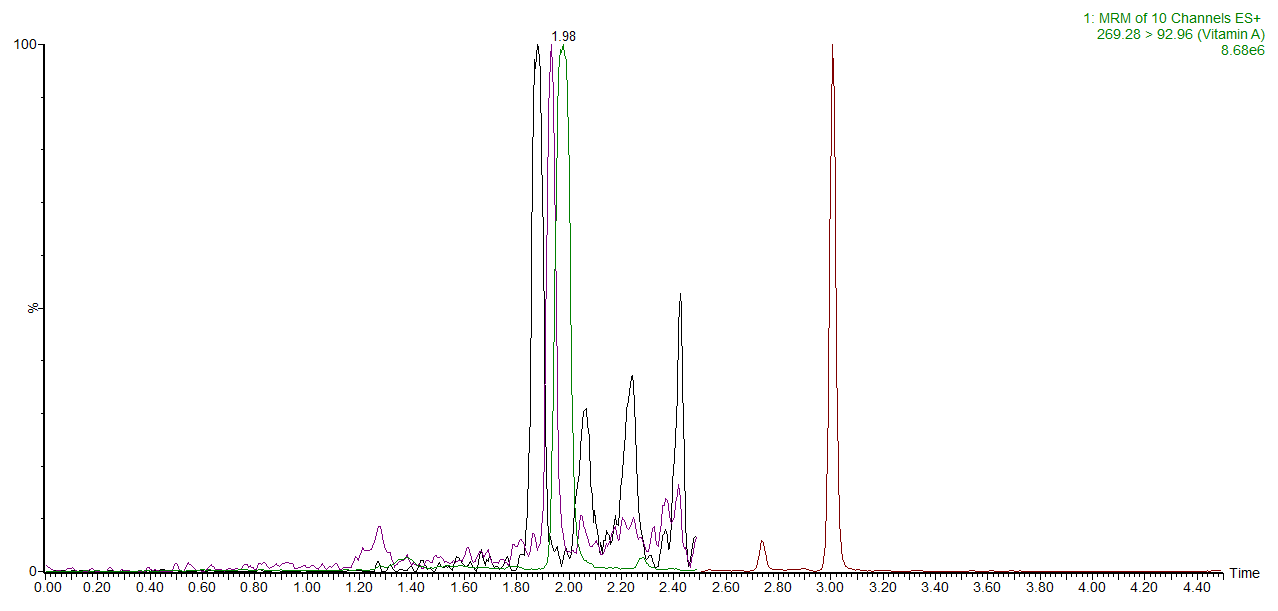


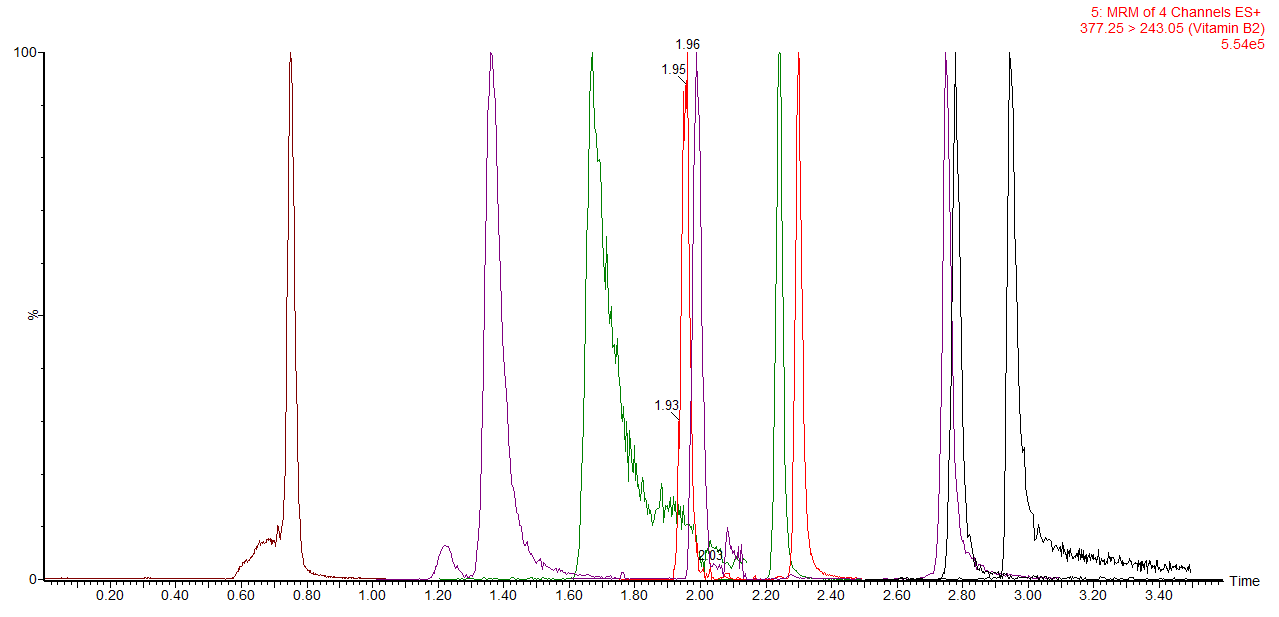


**Figure S4**


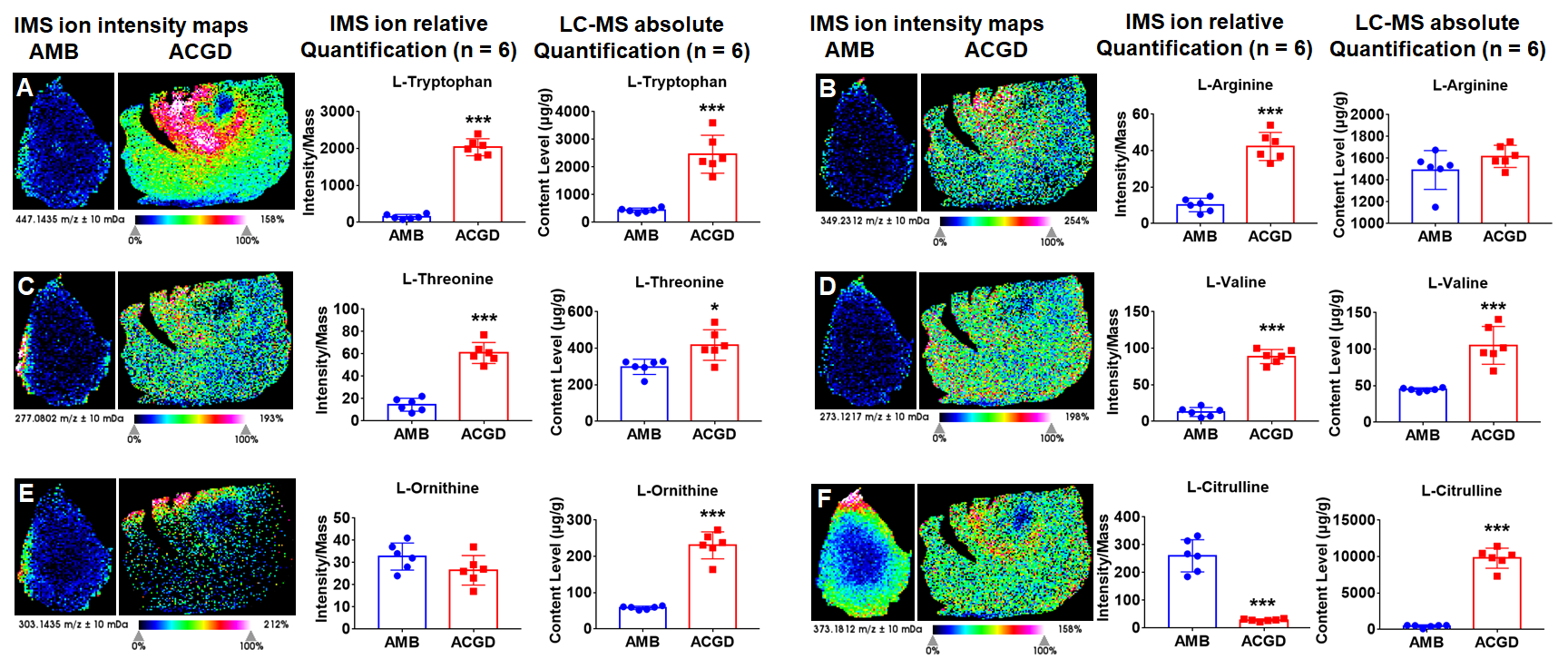


**Figure S5**


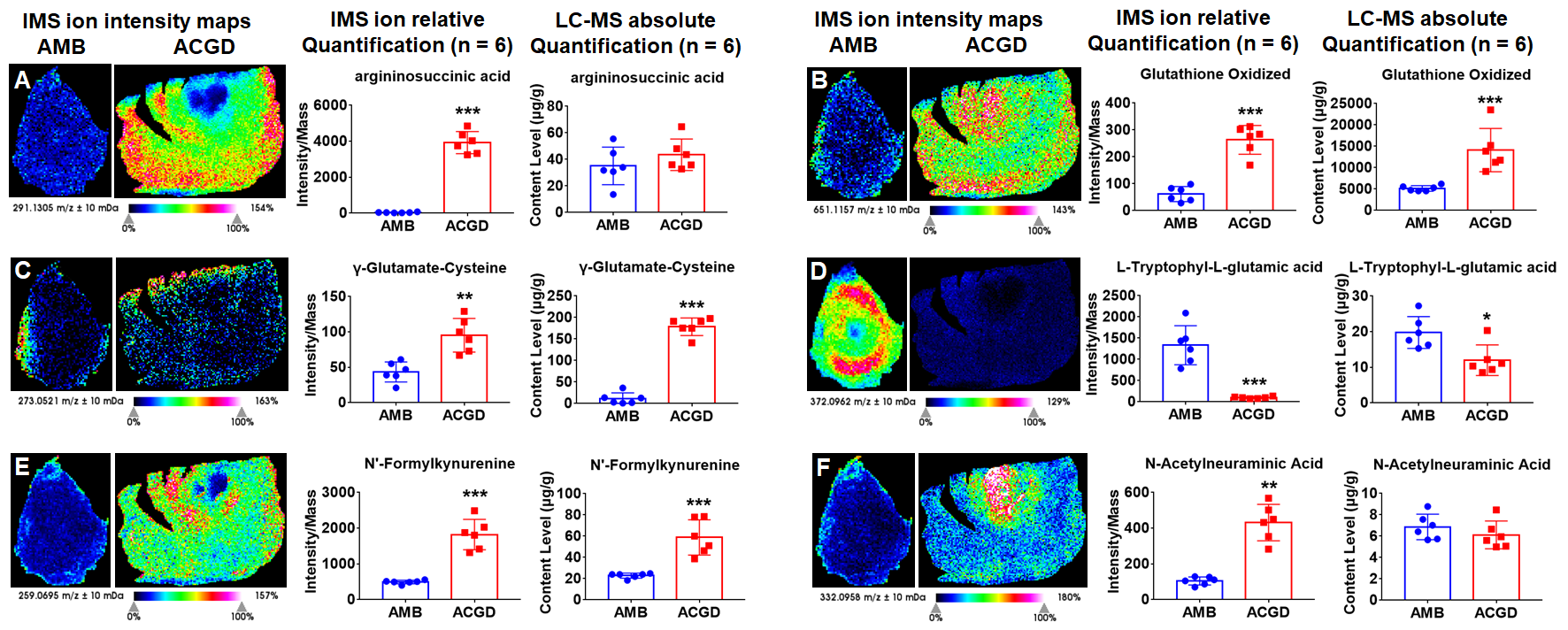


**Figure S6**


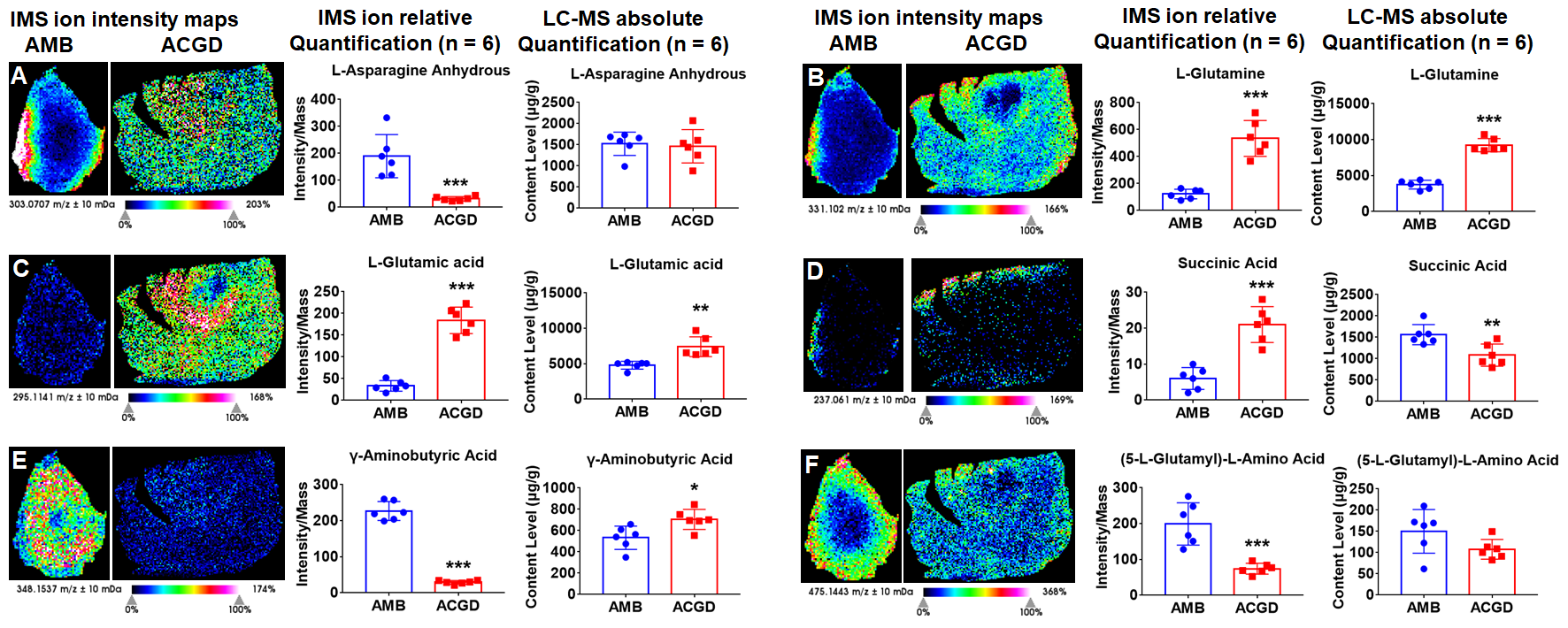


**Figure S7**


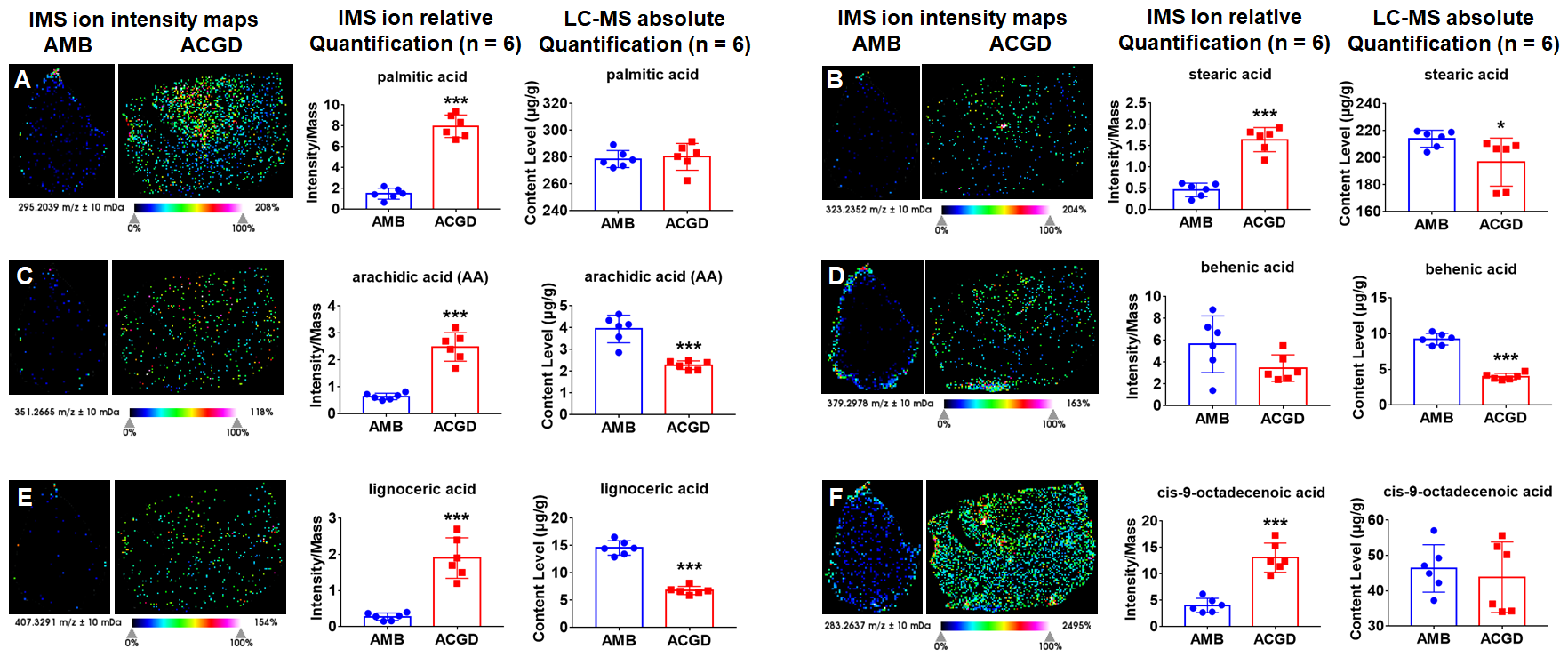


**Figure S8**


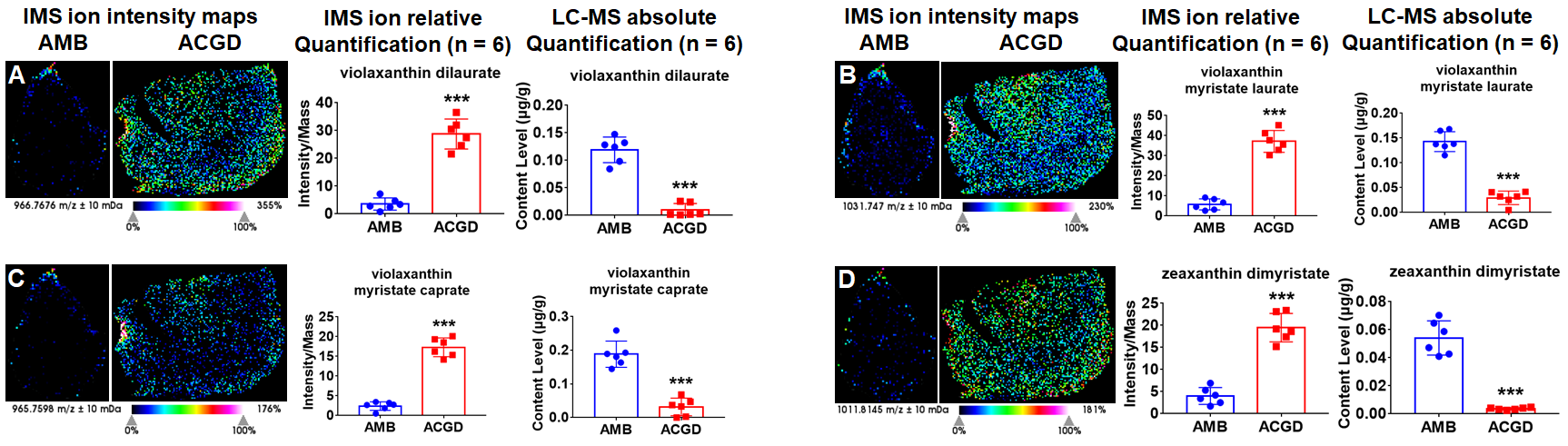


**Figure S9**


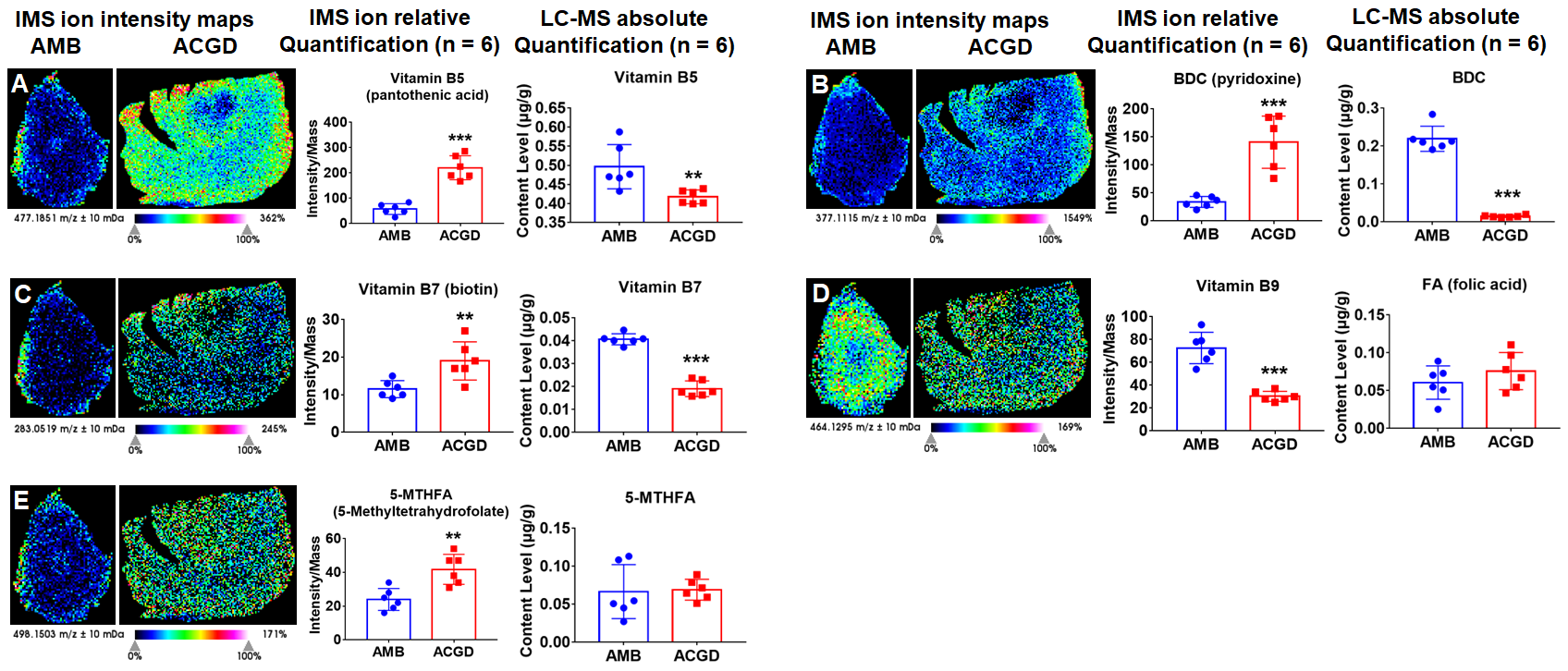


**Figure S10**


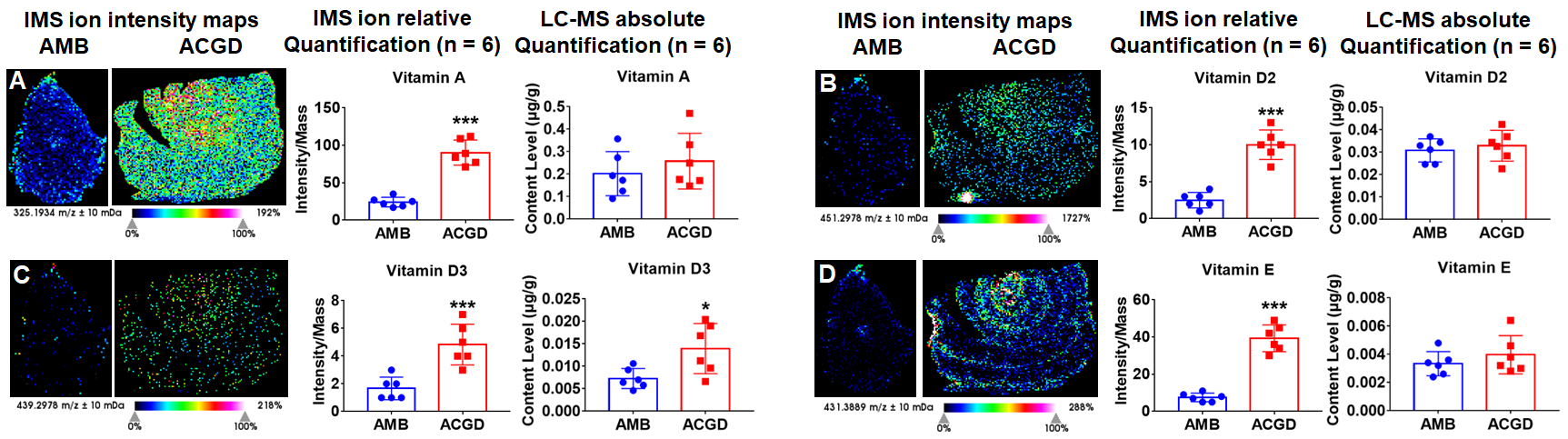


**Figure S11**


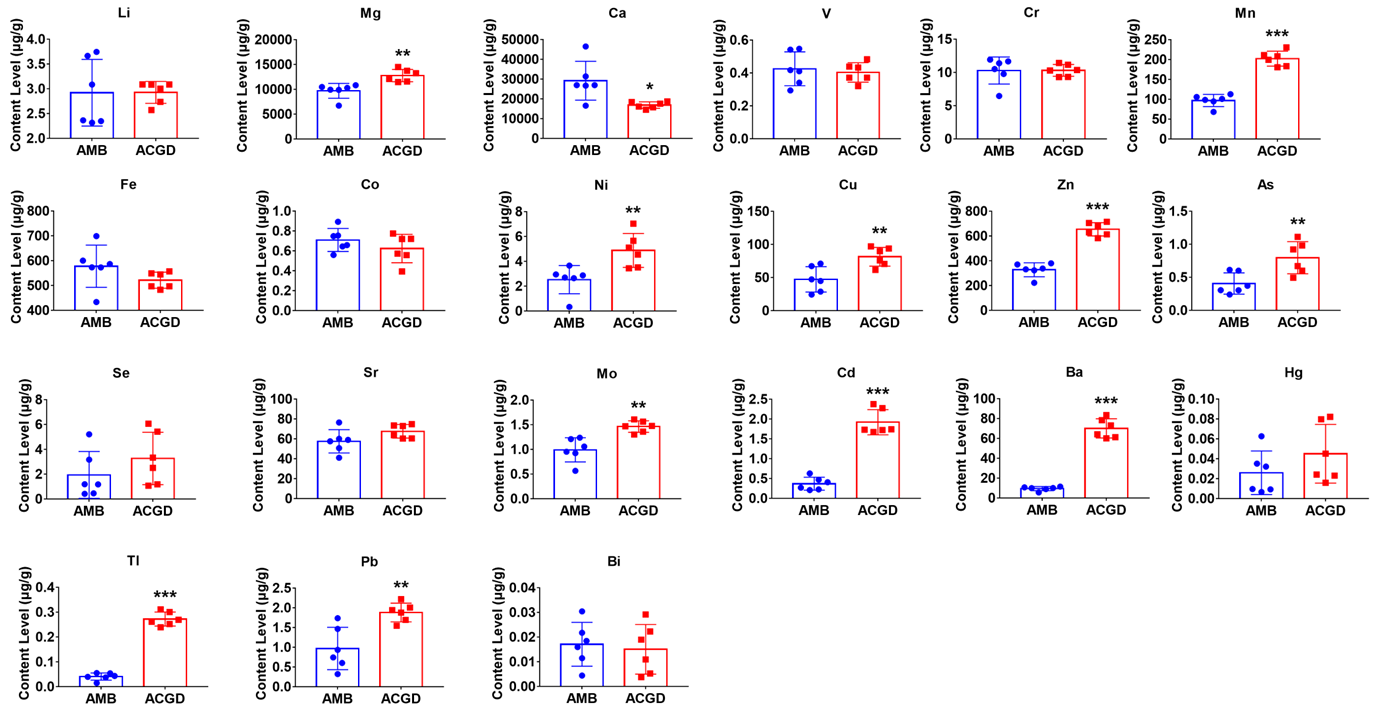


**Figure S12**
